# Supplementary material for: Pilot study on the effect of a Meditation–Mindfulness–Positive Psychology Training program on perceived stress and mental well-being in Korean nursing students: A mixed methods analysis
Source: PLoS One. 2026 Apr 21;21(4):e0345139. doi: 10.1371/journal.pone.0345139 (PMC13098892; doi:10.1371/journal.pone.0345139)
Supplement: S2 Data — Includes de-identified qualitative excerpts used in the inductive content analysis, organized by category (A–C). (DOCX) [file pone.0345139.s002.docx]

**Participant: P01**

**Date: 2024-04-01**

**Duration: 58 min 20 sec**

----------------------------------------------------------------------------------------------------------------

**R:** Just in case, I’m going to record this with a voice recorder as well. I can’t let this precious data fly away, so I’m being very thorough. Some people record multiple copies... surely the heavens wouldn't interfere that much, but if they do, I’ll just have to accept defeat. You look a bit tired.

**P01:** It hasn’t been long since I woke up, so...

**R:** Yes.

**P01:** I'm fine, though.

**R:** It’s been about three weeks, going into the fourth week since our research ended. What year are you in now?

**P01:** I’m a senior now.

**R:** So you’re a senior... and you’ve just finished your first month of classes in March.

**P01:** Yes.

**R:** No wonder you're tired. Good. I didn’t send you the questionnaire beforehand. You can see this as an exploration process—regarding your overall experience with the program and how it influenced your life. The first thing I want to ask is: Was there a specific reason or purpose for you to join this group/program?

**P01:** Well, I’ve never really had psychological counseling before, other than the simple consulting sessions provided by the school. But this program had a very clear purpose—to take care of the mental health of nursing students. I thought that if I did this alongside my studies, the path to preparing for employment wouldn't feel so bleak. That’s why I joined.

**R:** I see. So your purpose was that you hoped it would help with your studies and job hunting.

**P01:** Yes.

**R:** What kind of help specifically were you hoping for?

**P01:** I felt like I wouldn't be able to take care of myself properly while being so busy. My goal was to take care of myself while also handling my studies—I didn't want to miss out on anything. Most of all, I wanted to learn how to care for myself first while managing my schoolwork.

**R:** Right. Nursing students have a very heavy load—clinical practice, academics, and job prep—especially in the 3rd and 4th years. I've observed that the stress levels are particularly high. You said you wanted to "take care of yourself." Could you tell me more specifically what that meant to you back then? What were your expectations?

**P01:** Learning how to think healthily... not comparing myself to others, not blaming myself for the thoughts I have, but just being able to accept them. I wanted to become mentally stronger.

**R:** I see. That makes sense as a reason for participating. Now, the program lasted 6 weeks. It could be long or short depending on how you look at it. You participated in the group, and unfortunately, there was a lot of homework, too. It could have been burdensome, but after following through for the sake of your goals, what changes did you experience?

**P01:** During those 6 weeks, because the program was ongoing, I felt like I had to do it. I think I put in more effort. Because I was doing it consciously, I said many kind things to myself and was able to become a calmer person. Since I had to meditate a lot—while walking or washing my hands—I learned how to be calm. Or should I say I enjoyed "daydreaming"? I had more of that time than before.

**R:** What do you mean by "daydreaming"?

**P01:** It’s more like... not thinking about anything. That’s more accurate. Just being still and tranquil without any thoughts.

**R:** I suppose that could be seen as the result of meditation.

**P01:** Yes.

**R:** How does that affect you or your life?

**P01:** When a negative situation arises... instead of acting too emotionally, I feel like I can detach myself. It’s like leaving my body there but my soul steps out, so I can judge the situation more objectively. Instead of getting angry immediately.

**R:** You look at it objectively... so while you used to react habitually—like getting angry—now you step back.

**P01:** Yes.

**R:** Then, if you don't get angry right away, what do you do instead?

**P01:** Instead of getting angry, I think about what’s happening... and what I’m feeling... what I want to do... I didn’t just act on it immediately. I accepted the situation. If it was a negative situation between people, I’d think, "Oh, they could think that way," and I didn't respond emotionally right away.

**R:** It sounds like you observed your own state, your needs, and your thoughts. And then you practiced "putting yourself in their shoes" (empathy). Instead of reacting, you chose—or tried to choose—an action appropriate for the situation. Is that correct?

**P01:** Part of it is that, and also, by doing that, the negative emotions like anger subside. That’s why I was able to respond more calmly.

**R:** Wow, that’s impressive. Hearing you talk, it sounds like the effect of meditation, but I also hear "Mindfulness." Looking at the situation and your own state objectively is mindfulness. It’s not easy to be mindful in difficult moments, so it’s amazing that you practiced that. Did you experience any other changes? I think you mentioned saying kind words to yourself earlier.

**P01:** Kind words... I did say kind words to myself during the Compassion part.

**R:** During Compassion?

**P01:** Self-compassion.

**R:** Yes, yes.

**P01:** While doing that, I’d pick a phrase. It wasn't a fixed phrase; it varied by situation. It did help to boost my courage in the moment, but I don't think it had a huge overall impact on everything.

**R:** So while it didn't have a massive overall effect, it boosted your energy in that specific moment?

**P01:** Yes.

**R:** Could you give me an example?

**P01:** Like, if I have clinical practice next week and I have to wake up early every single day. Instead of just ending it with "This is so hard," I’d say, "I can do it," or "Everything will be fine once it passes." If I do it like self-suggestion, the anxiety feels a bit less in that moment.

**R:** Do you still utilize that now?

**P01:** To be honest, once the program ended... I’m the type of person who needs someone to give me homework. Since no one is telling me "do this, do that," I’m not doing it as much as I did during the program.

**R:** Right, it’s hard to keep it up alone. Even so, though you do it less than before, is some of it still maintained?

**P01:** I think I do "Action Meditation" (Mindful Activity) occasionally.

**R:** Yes.

**P01:** And I think I’m still doing "Stress Perception" (Mindfulness of Stress).

**R:** What kind of action meditation do you usually do?

**P01:** I usually do it when walking home after clinical practice, or when washing my hands.

**R:** Walking and handwashing—those are things you do every day. It seems you’re doing them as meditation since you’re doing them anyway. Is there a difference between how you walk or wash your hands now compared to before?

**P01:** When I do action meditation while walking, I definitely notice things I would have missed before, like when I was just looking at my phone. I look around more, see the details of the surroundings, and I feel like I have more "mental room" (leisure). When I do it while walking back from work, it feels more like a "commute." I don't think about what I have to do once I get home; I just enjoy that specific moment.

**R:** It sounds like there’s a difference in your state of mind when you arrive home after meditating while walking versus when you don’t.

**P01:** Yes.

**R:** What kind of difference is it, comparing the two?

**P01:** On the side where I don't meditate, I always feel rushed. I feel like I'm being chased by a schedule of things I must do. But on the side where I do meditate, I only focus on that situation, so it feels like my life is flowing leisurely. I’d divide it into "hurried" vs. "relaxed."

**R:** So your state of mind changes like that. How does that affect your performance? Your actual tasks.

**P01:** In terms of actual tasks... I do the tasks the same way, but... the difference is just the "mental room" I have. Whether I perform the task while feeling rushed or with a lighter heart. I don't think the efficiency changes much, just the level of ease in my heart.

**R:** So having more "mental room" doesn't mean you become lazier.

**P01:** No.

**R:** Good. When do you usually do the handwashing meditation?

**P01:** I wash my hands very often, but I usually do the meditation at home rather than outside. When washing hands at home.

**R:** So you don't do it because of a specific event, but just whenever you wash your hands. Does it happen automatically now—washing hands like a meditation?

**P01:** Since I do it so often, it’s almost automatic now. I empty my mind and focus only on the act of washing. In that moment, I can focus entirely on that.

**P01:** That's how it becomes.

**R:** Yes, me too. I found that it happens naturally over time. Even for a short moment, the brain switches to that mode automatically. That’s why I asked. Regarding "Stress Perception"—Mindfulness of Stress is essentially the same thing—you mentioned you’re still doing it. After experiencing the program, is there a change in how you perceive and react to stressful events? You mentioned something similar earlier, but let me ask again.

**P01:** Before doing mindfulness of stress, I mostly felt stressed when I lacked time, usually when I missed a bus. In the past, if I missed a bus, I’d just stay in that anxious state, stomping my feet and waiting anxiously. I’d worry about the person waiting for me or the appointment time getting closer. I’d feel anxious, annoyed, angry, and resentful—like the world was intentionally trying to screw me over. I’d become very sensitive. But when I try to do the mindfulness, I think, "The world isn't trying to screw me over," and I look at what emotions I’m feeling and how my body is reacting. That helps me cool down my anger. By not ruminating on the fact that I’m late...

**P01:** I can dampen my emotional reaction and look at it more objectively. So I get less stressed if I do that.

**R:** So before you "looked" at it, you felt the world was trying to screw you over—you must have felt so resentful. It was just a missed bus, just bad timing, but you felt like the whole world had conspired against you. But now you look at it objectively, feel your body, and notice your emotions. What does that mean to you? To me, it sounds like you aren't "falling into" the emotion but observing it quietly. In a way, it sounds like you are being understanding toward yourself. Is that right?

**P01:** Yes, that’s correct.

**R:** Could you elaborate on that a bit?

**P01:** I think the expression "understanding myself" is correct. I didn't miss the bus because I wanted to, and the bus didn't pass me because it wanted to. So...

**P01:** I just think, "That can happen," and I move on.

**R:** What about the emotions? It was a stroke of bad luck. It wasn't because you were dawdling; you tried your best, but things went wrong. You might feel sorry toward the other person, annoyed, or angry—it’s a stressful event. Many emotional reactions will arise. How do you respond to those emotional reactions? What do you think or feel about them?

**P01:** My reaction to what?

**R:** Your reaction to your own emotional state.

**P01:** My emotional state...

**R:** Yes, the anger, annoyance, or guilt... complex emotions that rise up when a stressful event occurs. What is your reaction to those?

**P01:** At first, of course, I feel resentful, angry, and annoyed—all those complex feelings rush in. But if I carry that with me, I’ll be in a bad mood all day. It’s not polite to the person I’m meeting to have fun with. So I try not to drag those emotions further. I try not to feel those negative emotions anymore, so I "toss them aside" and look at myself from a distance. It’s like emptying my emotions. I empty the emotions and look objectively.

**R:** Mm-hmm.

**P01:** So I become calmer, the annoyance fades, and I become a more "understanding" version of myself.

**R:** I see. That’s great. You have to do clinical practice and training. You joined with the goal of getting help for those things. You probably did practice recently. Could you tell me what kind of impact it had?

**P01:** Usually, for practice, I have to leave very early. At some sites, I have to walk 10 to 15 minutes after getting off the subway. Doing action meditation on that walk makes me feel like the day is truly starting. I slowly look around and enjoy the surroundings, so it feels like a proper start. On days I meditated, when I take public transport and it’s super crowded and people are pushing... I "detach" myself again.

**R:** Because you're angry?

**P01:** Because if I get angry, I become more sensitive. By detaching myself, I get less stressed and feel more at peace.

**R:** Morning rush hour is a stressful situation. Being packed like bean sprouts in a jar is painful. But you’re saying that if you detach yourself, the stress goes down. How do you detach yourself in that moment?

**P01:** Even if people push me, if I get annoyed... I try not to let those thoughts snowball. If I think "Why is that person doing that?" it just leads to more thoughts and the emotion lingers. So I try not to think that way. It’s the feeling of detaching myself from the situation. My body is in the situation, but...

**R:** Mm-hmm.

**P01:** It’s a feeling that my soul is not in this place.

**R:** But they say if you try *not* to think about something, you think about it more. How do you manage not to think about it?

**P01:** Rather than thinking about my "emotions," I think about the "situation" itself. "There are just a lot of people." "This is what rush hour is like." Instead of "I’m angry," "This is annoying," or "I want to go home," I just try to think about the situation: "There are many people."

**R:** But annoyance can still rise up. It’s an unpleasant situation, after all. What do you do with the annoyance that rises?

**P01:** For the rising annoyance... usually, I just give up (accept it). But if it still rises despite my efforts, there's nothing I can do, so I just...

**R:** Mm-hmm.

**P01:** I feel the annoyance as it is and try to get out of that stressful situation as quickly as possible.

**R:** What do you mean by "feeling the annoyance as it is"? Do you express it?

**P01:** I just... feel annoyed. In whatever way.

**R:** Do you, like, hit the person next to you? [Laughs]

**P01:** No, not that. I don't vent it on others. I just stay angry by myself, or maybe complain to a friend. That’s about it.

**R:** Not inside the subway, but over the phone? Or after you get off?

**P01:** Via KakaoTalk.

**R:** I see. In that case, you text "I'm so annoyed because there are too many people," or you acknowledge "I'm really annoyed right now." Depending on your condition, some days might be worse. In that state of high annoyance, can you still see your state objectively? Do you ever do that?

**P01:** When I’m *that* angry... it’s because I already tried those methods before getting to that point and they didn't work. When I’m extremely angry, it doesn't seem to work well.

**R:** Right. When you’re completely "in" it, you can't detach. You're "stuck." So you're saying you can look at your state objectively *before* it reaches that extreme point.

**P01:** Yes.

**R:** You mean you look at yourself like, "I'm annoyed right now," "I'm suffering," "I'm having these thoughts," "I really want to go home." You see your state from a distance.

**P01:** Yes.

**R:** And I understand that by doing so, the annoyance naturally subsides.

**P01:** Yes. Oh, wait, my delivery just arrived. Can I go open the door for a second?

**R:** Yes, go ahead. It's fine.

**P01:** Thank you.

**R:** Deliveries feel like presents, even if it's my own money. Dogs don't seem to like the delivery man, but they like the food delivery person. [Laughs] Anyway, while doing this program, were there any difficulties or things you felt were missing?

**P01:** Difficulties... you taught us so many things, so it was fun to choose what to do, but because there were so many, it was a bit difficult to perform them all and check them off. That was a bit hard/difficult.

**R:** "Hard" is good, "missing" (disappointing) is fine too. Anything is okay.

**P01:** Something I felt was missing... this is more about myself, but I’m disappointed that I worked so hard during the program, but once it ended, I haven't been doing it as much. I’m disappointed in myself for that.

**R:** So during the program, having so much to learn was both good and tiring. At first, we did meditation, and then we moved on to mindfulness. How was that? Was it hard to choose then, too?

**P01:** During mindfulness?

**R:** We did meditation, then mindfulness...

**P01:** Mindfulness was okay because it didn't take a lot of time. And for meditation, action meditation was fine because I could do it while doing my own tasks. But for Yoga meditation or Breathing meditation, I had to set everything up and be fully prepared. That part was hard. It takes up time and requires total focus, so it wasn't easy to do it often.

**R:** I see. So things like Yoga, Breathing, or Body Scan meditation were hard to practice because they require preparation and a specific time slot.

**P01:** Yes. But honestly, when I actually did them, those were the ones with the most effect.

**R:** Which meditation suited you best or had the most effect?

**P01:** The one that "suited" me best was action meditation, but the one with the most "effect" was Yoga meditation. I think it was Yoga meditation.

**R:** Do you exercise?

**P01:** I play basketball now.

**R:** Since basketball requires a lot of thinking, you probably can't do it as "Yoga meditation."

**P01:** No, not really.

**R:** You have to use your head a lot, more than one might think.

**P01:** I have to use my head and communicate with teammates.

**R:** Right, so that doesn't work. Something like the gym is simple and repetitive, so you could use that as Yoga meditation... I’m actually participating in a program too these days. I’m a participant in a meditation/mindfulness program. I did a "full day" session once. Now I do it over Zoom, but I went in person for a full day... it was such an ordeal. I really resonate with what you said. They do Yoga meditation for 50 minutes. Breathing/Sitting meditation for 30–40 minutes, Body Scan for an hour. I don't know... it was very exhausting for me. Since I’m an honest person, I gave honest feedback, and the leader seemed a bit discouraged. I wondered if I was too honest. [Laughs]

**R:** But I personally like it when group members speak honestly. That’s how we can adjust in a good direction. I see this program as being in a state of growth/development, too. By hearing this from you, I can adjust the program next time. So I like honesty and thank you for being honest. It really was exhausting, right? It seems your sentiment is similar to mine. Even though our sessions were short, practicing them in daily life isn't easy. That’s why we keep emphasizing action meditation. "At least do that." But action meditation is good. And for breathing or body scans, you don't necessarily have to lie down; you can do a part of it while on the subway, or just breathe for 3 minutes.

**R:** Or "Five Senses" meditation is good too, while waiting for something—like the elevator. When I go to school for counseling, I see many college students looking at their phones even in the elevator or for a brief moment on the subway. I’ve made it a habit to just meditate during those brief moments... anyway, I’m rambling. Is there anything else you felt was missing? You said having too much to learn was a bit disappointing—would focusing on just a few things be better?

**P01:** Rather than the number... there were many, but they were all necessary things, so I can't really "reduce" them. Knowing them all is good because you can use them when needed. So reducing them is a bit...

**R:** Yes.

**P01:** Maybe just reduce the *frequency* of the meditation [assignments]?

**R:** So you think it’s better to learn everything. But when it comes to practice, you can just pick what suits you and do it consistently. That’s how I’m summarizing it. Besides those, we did Compassion, Gratitude, Well-being Perception, etc. How were those?

**P01:** Those things allowed me to think about myself again. It felt like self-reflection. It made me look back at my life, and I felt more gratitude for my life.

**R:** So it was an opportunity to rethink yourself and your life. But it’s hard to do it consistently?

**P01:** Well, for "Well-being Perception," I think I can keep doing it depending on the situation. And for self-compassion, I can just say the phrases. I think I could do it consistently if I tried.

**R:** We also wrote journals—like well-being journals. Is it hard to keep those up alone?

**P01:** Yes, honestly, keeping a record/writing it down isn't easy. Just thinking about it is fine, but...

**R:** Yeah, I totally agree. It’s not easy. But some people really utilize that, so it seems there are individual differences. Some people just do it in their heads, some love recording it, and some intentionally use imagery. So I think the method should be tailored to the individual. Any other disappointments? Earlier, you said you were disappointed that you don't do it alone now that the program is over. But it sounds to me like you're still doing action meditation and mindfulness in your daily life. Why do you think you aren't doing the other things?

**P01:** For the other things... during the program, there was a visible record—checking things off. So I did it. But now that it’s over, no one is holding me accountable. If I do it, I’m the only one who knows. I can't share it with anyone. So I became lazier and stopped doing it. Also, things like "Sensory Meditation" or "Action Meditation" can be done while living my daily life, but other meditations require an investment of time, so I didn't really do them.

**R:** So if there was someone to do it with, or a system of encouragement and verification, you could keep it up?

**P01:** Yes, I think so.

**R:** Like a group chat where participants "verify" their practice—a lot of people study that way these days. If that was available, would you have the intention to continue or participate?

**P01:** Yes, but only if it’s a group of people who are truly committed to doing it.

**R:** Right. Exactly. Some people do use those kinds of rooms. If such a room was used, which of the things we did would you want to do together?

**P01:** Meditation, and the words... the words you say to yourself.

**R:** Those things?

**P01:** Deciding on your own phrase and saying it in certain situations.

**R:** We call that "Well-being Perception"—intentionally using thoughts. Anything else you’d want to do together?

**P01:** Self-compassion? Writing a gratitude journal would be okay too.

**R:** Writing a gratitude journal together. We call it a "Well-being Journal," but yes. Earlier you said your thoughts about yourself or the world changed. After the program, is there anything new you learned about yourself, or has your thinking about yourself changed?

**P01:** While doing "Stress Perception"...

**R:** Yes.

**P01:** By doing that, I recognize the situation where I’m feeling stressed. By doing so, I realized, "Oh, I’m under a lot of stress."

**R:** Mm-hmm.

**P01:** And I realized that I was actually capable of detaching myself from a situation and looking at it from a distance. "I can do this too."

**R:** I see. Does knowing "I am under stress" change your attitude toward yourself?

**P01:** Since I know what situations cause me stress, I’ve tried to avoid those situations, and since I do the stress perception each time, I think I’ve gained a coping mechanism.

**R:** Avoiding the situation is a coping mechanism, and if you can't avoid it, you’ve gained a way to deal with it. For example, what kind of coping mechanism did you gain?

**P01:** I think the coping mechanism is just that—Stress Perception itself.

**R:** I see. It has that functional role. Did participating in this program change your relationship with yourself, or with professors, friends, family, or patients during practice?

**P01:** First, regarding the relationship with myself, I feel like we’ve become more intimate. I kept thinking about myself—what makes me happy, what situations stress me out—so I feel closer to myself. Also, in the last session, we wrote what we thought our moms would want from us and what we wanted from our moms. Doing that made me think from my mom’s perspective, so I could look at relationships with others, not just myself.

**R:** You don't write it out formally now, but do you "practice" or "execute" that in your head?

**P01:** I haven't been able to practice that one. Other than that...

**P01:** When doing self-compassion, I also did compassion for others. At the end, I even wished compassion for someone I don't particularly like. Doing that seemed to soften my resentment toward them, and I think I understood their situation a bit more.

**R:** Was that something you did in the group? Or practiced alone?

**P01:** I think I practiced it alone. When we did it in the group...

**R:** Yes.

**P01:** I only did compassion for myself and people I like.

**R:** Yes.

**P01:** But later on, I felt like I wanted to do it for that person [I disliked], too.

**R:** Oh.

**P01:** So I think I did it.

**R:** You're better than me. I don't really do it for people I dislike. I should expand my own power of compassion, too. I’m reflecting on myself now. Anyway, it seems you were able to practice empathy in your relationships. By empathizing, you understood them more, and in a way, you didn't get as angry—it became more comfortable.

**P01:** Yes, you could see it that way.

**R:** Thank you. Is there anything about the program you think should be changed?

**P01:** About the "sharing reflections" part... I’m not sure how to say this, but would it be too burdensome if the order of sharing was fixed? When it was time to share, it was always so silent. I usually went first because the silence was so suffocating. I don’t know how to make it more flexible, but... anyway, I wish the sharing of reflections was more fluid/flexible.

**R:** So the silence when waiting for volunteers was uncomfortable for you. That’s why you volunteered first—to break that discomfort. Thank you for that spirit of sacrifice. So you’re saying it might be better to just assign the order.

**P01:** That might actually be better. Once you ask them specifically, they usually speak very well.

**R:** Right, that’s a dilemma for me too. If I assign it, some people feel very uncomfortable. I don't want to seem like a dictator or be too forceful. But in the end, I usually end up making everyone speak. Looking back, it might be better to just be a "dictator" and go in order. Because if I don't ask, as you said, we might miss out on such helpful words. Those words help me and the whole group. So it’s a dilemma, and I really rely on people like you who can't stand the silence and speak first.

**P01:** Thank you.

**R:** You've spoken so well. How do you feel? We’ve been talking for an hour—how was this interview for you?

**P01:** Seeing you again makes me feel like I should work harder. I’ve regained the will to try the things I had forgotten.

**R:** Which things do you feel the will to try again?

**P01:** Using the files you sent...

**R:** Yes.

**P01:** I want to try Yoga meditation and Breathing meditation. I want to make some time for them.

**R:** It might be good to slip a little "Compassion" at the end of the Yoga and Breathing.

**P01:** Yes, thank you.

**R:** Thank you. Goodbye.

**Participant: P02
Date: 2024-04-02
Duration: 69 min**
----------------------------------------------------------------------------------------------------------------

**R:** There’s no audio-only option [on the platform], so I’ll just use the audio later. As you know, all personal information will be anonymized. You’ve read about qualitative research, right?

**P02:** Yes.

**R:** There’s nothing in there that can identify you personally, right?

**P02:** Yes.

**R:** Thank you. Have you ever done an interview for qualitative research before?

**P02:** I don't think I've ever done an interview.

**R:** Well, you’re adding a new experience. It’s nothing special—we’re just exploring what your experience with the MMPT program was like and how it overall influenced your life. I didn’t send the questions beforehand, as you know. So, please just speak honestly and comfortably as things come to mind. That will help in running the program better in the future. Honestly, it’s a bit of a limitation for the researcher who conducted the group to also do the interview. We’ve grown fond of each other, so you might not be completely honest [laughs]. Originally, a third party should do it, but I trust that the participants will give their bold and sincere opinions. I'll proceed with that trust.

**R:** Is there anything you'd like to ask or say before we start?

**P02:** No. I think we can start now.

**R:** You said you're "waiting" after graduation. What does that mean? You’ve been hired, but you’re waiting for your official assignment?

**P02:** Nursing students get jobs during the summer break of their senior year. So, as a "prospective graduate," my employment was confirmed last summer. The start dates are usually in March, May, June, September, or even November. You either go in when you want or where the hospital assigns you. I’m scheduled to start in June.

**R:** June... you have about two months left. It must be a "honey-like" (sweet) time for you. How is it?

**P02:** Honestly, because of the current doctors' strike, even the existing staff are having trouble operating. I'm not sure what will happen. It was completely confirmed, but suddenly things became uncertain, so I do have some anxiety.

**R:** I see. Still, surely nothing big will happen... you're just worried that the waiting period might get longer?

**P02:** Yes.

**R:** Right. The first question I want to ask is: What was your reason or purpose for participating in our MMPT (Meditation, Mindfulness, Positive Psychology Training) program?

**P02:** From the perspective of someone about to start working, the "newbie" period is quite difficult. And for nurses, the turnover rate within the first year is very high—statistics say almost 50% quit within a year. So, managing stress early on is crucial. I wanted to use this opportunity to look into that.

**R:** So, your employment was decided, but you wanted to prepare for the stress of the first year, including the 3-month training period?

**P02:** Yes, it’s about 2 to 3 months depending on the hospital.

**R:** They say that first year is the hardest. You wanted to prepare for that. Back then, you weren't under much stress, but you wanted to prepare for the future?

**P02:** At that time, yes. It was my graduation season after the national exam. It wasn't that something big was happening *then*, but I saw it as a "life skill"—the ability to manage my stress and my state. I had participated in a similar study before, a counseling program, and what I learned then was very helpful. So I applied thinking I could get help this time too.

**R:** What kind of program was that previous one?

**P02:** It was about 8 sessions... what should I call it?

**P02:** They counseled us on stressful situations and gave us assignments. Things like how to interpret a stressful situation, recording my emotions about a situation... I remember it was a study that screened students with high stress scores and compared them before and after.

**R:** That one had assignments too—like a journal?

**P02:** It wasn't exactly a journal, but I remember an assignment about "challenging negative beliefs" regarding an event. It’s been a few years so I don’t remember clearly, but it was a study that helped me shift my perspective on the difficult situations I was facing back then.

**R:** I think I know what that is. When was that?

**P02:** I think it was 2020.

**R:** Around your freshman year of college?

**P02:** Around the second semester of my sophomore year.

**R:** I see. It’s a bit different from our program. Anyway, that was helpful, and this time you joined to learn management methods specifically for the big stress ahead. So, what kind of changes did you experience personally through the MMPT program?

**P02:** Through MMPT?

**R:** Yes, MMPT.

**P02:** I used to ruminate (dwell) on my mistakes. If I made a mistake, I’d keep thinking about it to the point where I couldn't sleep. It’s already passed, it’s not a big deal, but I’d constantly repeat thoughts like "Why did that happen?" I didn't really know how to liberate myself from those thoughts or how to shift my attention. But in the first week, we practiced focusing on sensations, right? Sensory meditation. Doing that, I learned that there’s a physical limit to the energy we can spend. If we intentionally focus on sensations, we realistically can't use energy on other thoughts. Knowing that... when I’m about to get absorbed in a thought, if I shift my thoughts to the physical sensations I’m feeling now, I realized I could free myself from ruminating and repeating thoughts all night.

**P02:** From that first session, it was very beneficial, and I found myself looking forward to the next sessions.

**R:** So, understanding the *principle*—and then focusing on sensations—was more helpful than just doing it without knowing why.

**P02:** Yes, that definitely helped.

**R:** Then, even though we practiced together and experienced it in the group, knowing the theory and actually doing it in reality can be different. How did you apply it in your actual life?

**P02:** I wasn't in a period of high stress, but I have a lot of travel time. I do various jobs and hobbies, so I travel a lot. I always had this obsession with self-development—feeling anxious that I should be reading a book or studying a foreign language on public transport. But the subway is so crowded. In that space, I practiced observing things around me—the sounds, the sights, the smells—and I was able to practice controlling that anxiety. It was good.

**R:** So you utilized "Five Senses Meditation" in short bursts on the subway or during transit to regulate your anxiety. But you mentioned you used to ruminate a lot.

**P02:** Yes.

**R:** Even to the point of not being able to sleep. Does this meditation help with that habit of ruminating?

**P02:** That happened a bit early on [in the program], and not much after that. But if such a thought occurred, I tried hard to shift from the thought to the immediate sensory experience. Just knowing that such a method *exists* was a big help.

**R:** That makes sense. "If a thought pops up, I can just shift to sensations." How exactly does just knowing the method help?

**P02:** When I talk to people around me, everyone has their own life problems. When I hear them, I can tell them about this. When they are struggling with something, I can say, "Stop that thought. It’s in the past. Just learn what not to do next time." You can't go back by thinking about it. If it’s too hard because of the thoughts, just focus on what’s in front of you—what you’re experiencing now. I can't explain it professionally like a teacher, but I could tell them that if we immerse ourselves in something else, the energy going into [the stress] will decrease. I received a lot of help in that regard.

**R:** When you pass this on, do they understand and actually use it?

**P02:** I haven't tracked if they actually use it...

**R:** It actually takes a lot of practice. But I can tell you practiced with focus, which is why it worked for you. Knowing it with your head and being able to do it is different. We call it a "skill." It's great that you can tell others, but for you, P02, does just knowing "I can shift to sensations if I'm distressed by thoughts" give you help?

**P02:** Before, I didn't know how to cope, so I just kept thinking. Now that I know, I’m not anxious. Even if a situation that used to distress me occurs, I know I can just learn what I need to, avoid the bad parts, and I don't have to think too much. I feel like my emotions are more under control? I definitely feel that.

**R:** I can see that. If thoughts pile up and you lose control, that’s when it gets hard. You might have anticipated that, but now you have the "I can just do this" thought, which might give you a sense of relief or a sense of control. Also, what kind of changes did you experience in the way you perceive and react to stressful events?

**P02:** When we did "Mindfulness of Stress," I practiced looking at myself—the one experiencing the stress—objectively. Recently, since I’ve been traveling and wrapping up my previous work, I’m in a situation where I have no income. I saved up enough money to use until I start my job, so it’s fine, but not having an income feels different than just having money. Regardless of the amount. So, I found myself getting stressed every time I spent money. Looking at that situation, I realized, "Oh, I'm thinking I'm stressed because I have to spend a large amount of money in this situation." Instead of being swept away by it, I started looking for a solution. If I leave myself in a vague state, the stress is too much. By objectively recognizing that I'm stressed in this situation...

**P02:** I allocated a budget for myself for the next few months. Because I worked hard for that money, I have the right to spend it. I organized how much to spend on what and gave myself the "freedom" to spend within that allowed range. In the past, I would have just been stingy and agonized every time I spent money, but recognizing "This situation is a stressor for me" led me to think about solving it. I think the fact that I didn't just stay stressed was a good change.

**R:** That's amazing. That is exactly what mindfulness is, but it's not an easy skill. Looking at oneself objectively is very difficult. I feel like you've been practicing in your spare time, which makes me feel proud and happy. I’m practicing mindfulness on myself right now, noticing "Oh, I'm feeling this way" [laughs]. In the past, you would have been buried in the event, feeling anxious and acting reactively—like "I shouldn't spend anything!" out of fear. But this time, you realized you were stressed. I guess in the past, you didn't even realize you were experiencing stress?

**P02:** Yes.

**R:** Because you recognized it, you were able to choose and act in a way that actually helped you.

**P02:** Yes.

**R:** But how were you able to realize it? In the past, you just experienced it unconsciously, but this time you saw it: "I am experiencing stress," "I am very anxious," "I'm worried about running out of money." Was there a specific method or effort that allowed you to see all that?

**P02:** I have a hobby I really love. But it requires going to a specific place and socializing, so it costs some money. I've always done it because I love it, but at some point, I started hesitating: "Should I go today or not?" I wondered why I was hesitating about something I truly love, and the root was the money issue. "Should I just stay quiet during the week and only go on weekends?" Because during the week I can only do it for an hour or two, so it feels like a waste of money... I discovered myself having these thoughts. So I thought, "Why am I hesitating about things I used to do without a second thought?" and I realized the recent situation—quitting work soon, spending a lot on a trip, and having about two months left before the new job...

**P02:** Thinking about all this, I realized, "I'm stressed about spending money without an income." I realized that. In the past, when I was a student and had no money, the stress was uncontrollable. I couldn't even get my boyfriend a proper birthday present, and that was so hard. Back then, I just accepted "I am stressed," but now, after studying mindfulness, I look at it from a step back: "I am having the thought that I am stressed." Then I think, "What can I do for 'me' who is feeling this stress?" and that led to the solution of planning a budget—categorizing hobbies, friends, education, medical—allocating it to myself and letting myself spend within that.

**R:** It sounds like your attitude toward yourself has become more compassionate.

**P02:** I think so. Yes.

**R:** By stepping back and looking at yourself objectively, you saw "Oh, she’s struggling. She wants to go, but the money..." They say spending your savings without earning is like "picking off the dried persimmons" (eating away at your reserves). Even though you have enough persimmons to eat, seeing the empty spots makes you anxious, and you saw yourself unable to do what you wanted. Instead of just saying "Don't do it!", you looked at yourself with pity and compassion.

**R:** Moving on... you haven't done any training yet, right? After our program.

**P02:** Training?

**R:** I mean clinical practice. You probably haven't had clinicals since then. So my next question was about the influence on clinical practice, but since you haven't had any, let me rephrase: You’re about to start your life as a nurse. What kind of influence do you expect then?

**P02:** This is common for friends in other jobs too, but when you first start working, your mood and state are heavily influenced by every single word from your "Sasu" (mentor/senior). You get this strong desire to be recognized. Of course, that’s important. I saw it during my student clinicals—words like "What would we have done without this student?" or "You don't even know this?" really affect your mood. And if they don't help you, work life becomes hard. So, external recognition is something I can't control, but the one who can think "I did this well today," "I did this wrong," or "I should fix this" is me. When I start working, if I only dwell on the mistakes and go in a direction of self-destruction... since there will be many mistakes at first, it would be too hard. So, I felt I should step back, observe my stress, and instead of being immersed in it, understand *why* I'm stressed and move toward thinking of alternatives. For example, if I got scolded for a mistake due to lack of knowledge, I can admit "I feel depressed and upset." After acknowledging that, I can decide to study that part, or if I was unfairly criticized, I can meet my colleagues/peers who can empathize and resolve it together. I think my direction is changing toward "managing" and "resolving" rather than just being stuck in the stress. I've realized I should move toward protecting myself.

**R:** That sounds like a method you are planning to use when you start working. Anything else?

**P02:** If there's anything else... I'm not sure if it's related to the future clinicals, but...

**P02:** Yoga meditation. I don't do yoga, but I usually do strength training or stretching. I love exercising. But if I stop, it's hard to start again. Starting work will be physically demanding, and as I move from my 20s to my 30s, I think exercising is very important. I feel I got help in the sense that I should exercise even if I use meditation as an "excuse." Because I *have* to exercise, and I have to meditate, I'll think "I'm exercising to meditate" and keep it up even while working.

**R:** So you’ll do strength training or stretching *as* meditation. That’s Yoga meditation. If you just exercise, it's just exercise. But when you do it as meditation, paying attention to the sensations... even just raising an arm can be Yoga meditation. You have a plan to use that to manage both your physical strength and mental state. How do you think that will help?

**P02:** In the past, I felt like the time spent exercising was a waste. I always had this idea of multitasking—useful use of time—so I’d watch a drama while exercising. If you fill your time like that, there’s no time to "come down" or resolve things. You’re just constantly filling. When I exercise now, I won't be in that "saturated" state. If I focus my mind on my body while exercising, my accuracy will go up, I’ll get the maximum effect, and by focusing on my body, I can liberate myself from the day's stress. I'm expecting the effect of stopping the thoughts and focusing on my physical self.

**R:** That’s great—getting the workout and clearing the mind at the same time. It’s a time to empty. Suddenly I want to do it too! I’m actually participating in a program too, to get a certification in meditation. I'm listening to the mandatory lectures, so I have homework too. I was supposed to do it today but I kept putting it off. I tell you all to do your assignments, but here I am lagging behind [laughs]. I have to do it by tomorrow. But I realized assignments are necessary; without them, I wouldn't do it at all. In my program, the focus is entirely on meditation, so the sessions are very long. They make us do sitting meditation for 40 minutes. Then Yoga meditation for 50 minutes to an hour.

**R:** And Body Scan... they make us lie down and do it for an hour, so people start snoring like crazy. Experiencing that kind of intensive, long meditation gave me a lot of empathy and learning. Honestly, it's very hard for me. Doing it so long and with such intention... the assignments are burdensome. So if you do yoga in the evening, I think if it's too hard, you won't do it. If you do it without too much pressure, you might be able to sustain it longer. Just my two cents... I'm supposed to be the interviewer, but my "professional disease" (habit) of advising is coming out [laughs]. Anyway, were there any difficulties or things you felt were missing?

**P02:** There are many techniques—Mindfulness of Stress, Well-being, Compassion... they were all in the later part. It’s unavoidable, but I wish I had learned them earlier. Is there a fixed order for these?

**R:** So you're disappointed that things like Stress Mindfulness, Well-being, or Compassion weren't placed earlier?

**P02:** No, not that... just a feeling of "I wish I knew this sooner." Honestly, when I first heard of Sensory Meditation, it was completely new and amazing. "Oh, there’s such a thing!" So I liked Sensory Meditation, but the things that came later were also good. I just felt it was a shame I learned them so late. I don't necessarily think the order should be changed.

**R:** What were the difficult parts?

**P02:** Breathing meditation...

**R:** Yes.

**P02:** You gave us the audio files, right?

**R:** Yes.

**P02:** For breathing meditation, it didn't work well without the file. So I didn't do it much in my daily life.

**R:** So it worked well during the live sessions, but not so well when doing it alone without the file?

**P02:** No, it works well *with* the file...

**R:** Yes.

**P02:** But doing it entirely on my own didn't work well. Only breathing meditation was uniquely like that. Others were fine.

**R:** I see. You could just use the file, but having to turn it on is another "hurdle." Which one works best for you?

**P02:** Personally, I think it's Sensations.

**R:** There are individual differences in what works and what doesn't. But ultimately, this is "attention training." You can just focus on what works best for you. If you keep doing what works, your ability to use your attention will grow. Meditation and mindfulness are all a type of attention training. By doing it, your "attention muscle" grows. Exercise is hard at first but gets easier as your muscles grow. Like students memorizing English words—it's hard at first, but I heard of a student in Daechi-dong who was made to memorize 300 words a day for a month. At first, it was brutal, but later it was no big deal.

**R:** That's because the "power to memorize" itself grew. Just like that, if you work hard on what works (Sensory), your muscles will grow, and naturally, breathing meditation will become easier later. It might not be as "fun" as sensory, but... for me, Body Scan didn't work well at first.

**P02:** Hmm.

**R:** But later it did. I totally resonate with what you're saying. And to explain the order—you have to "empty" first to be able to "fill" later. Once the rumination/dwelling is somewhat regulated and you feel more at ease, then you can step back and look at yourself. To use your attention to step back, you need to be able to regulate yourself somewhat comfortably. I’ve tried changing the order before, but this order seems to be the one. Looking at participant reactions... there’s a reason for it. Any other disappointments?

**P02:** Each session was an hour and a half, right? It felt a bit long to me.

**R:** The time was long?

**P02:** The number of participants kept fluctuating. Some people love sharing their experiences, and some love listening, but for me... if the group is large and the wait is long, it gets a bit boring. That part was hard when there were many people.

**R:** Listening to others' reflections?

**P02:** A few people are fine, but when it's 7 or 8 people, it gets boring. And I have to remember what I felt so I can speak, but then I forget. I don't know what others think, but for me, maybe 5 to 6 people at a time would be ideal.

**R:** I see. That makes sense. Good. My next question is about how you are using what you learned now that the program is over. It seems you are consistently using Stress Mindfulness, and for meditation... you mentioned Sensory Meditation?

**P02:** Yes, I'm doing Sensory Meditation well, and since I've been stretching every morning lately, I'm doing Yoga Mindfulness then.

**R:** Excellent. We taught many things—anything else you're using?

**P02:** I wouldn't call it a formal "Compassion Practice," but I've always been the type of person who wants everyone to be well. But I think I'm doing it more *consciously* now when I meet new people. I meet many new people through my club activities, or see people I haven't seen in a while. We talk, and if I hear someone is starting a new business or has something going on, I consciously think, "I hope those four aspects [of their life] go well." I believe that I can be happy only when there are happy people around me, because I am someone who is easily influenced by others.

**P02:** I think I've become more conscious of doing compassion for people I meet for the first time. Originally, I took good care of people close to me through actions, but I wasn't that way with strangers. It’s nothing grand—just me saying to myself, or saying a word to them like "I really hope your new venture goes well." Saying those positive words and keeping my thoughts positive feels better for me. Does it help the listener? I don't know, but it's better than saying something bad.

**R:** Of course it helps. It makes your heart comfortable and your attitude toward them easier. As the saying goes, "A single word can repay a debt of a thousand nyang." If someone feels bad after hearing you wish them well, that's their problem [laughs].

**R:** What would you do if they took it the wrong way? Nothing you can do. Anyway, has anything changed about yourself? There’s the relationship with others, but there’s also the relationship with *yourself*. Earlier, I felt that you've become more compassionate toward yourself. Is there anything new you learned about yourself or any change in your thoughts?

**P02:** Through this program, I learned ways to control my emotions. Recently, after graduation—I think it was this March—I met a college friend. We talked about how we’ve grown between entering college and graduating. Looking back at the past four years, I felt like I gained the "skills" to solve my own problems. I felt I’ve become more emotionally mature. Back then I felt like such a "student," but now I feel like a "college graduate." I felt my growth in that area. Before that... well, I’m a nursing graduate who hasn't even started working yet.

**P02:** I graduated from a "Specialized High School" (Mokgo), and there were many smart kids around me. Looking at my friends... I’m two years behind because I retook the exam and took a leave of absence. Some friends have finished their Master's and are starting their Ph.D. this year, some started companies, and some went to international organizations abroad. Hearing about them, I sometimes felt so shabby. "I just graduated from nursing school and I'm not even earning money yet." But instead of comparing myself to others, when I looked back at what kind of person I became through college, I saw: "I used to be easily swayed by others' opinions, didn't know how to handle stress, and couldn't control old childhood wounds." But through college, I participated in research, went to the school counseling center... the efforts I made to control my own life seem to be showing results. I felt my growth there.

**P02:** I was able to confirm that I worked hard in college, so I was able to compare my current self with my past self, rather than comparing myself to others. I think that was a very good outcome.

**R:** "I’ve worked hard for myself. I’ve grown." You’ve acknowledged yourself in that way. You feel proud.

**P02:** Yes.

**R:** And as I listen, you’ve always been compassionate toward others. But has the program made a difference in how compassionate you are *toward yourself*? Is there a connection there?

**P02:** Hmm...

**P02:** What would be the answer?

**R:** You're looking at the ceiling while you think! [Laughs] Is the answer written there? My question might have been vague. It's okay. I guess you were already somewhat compassionate toward yourself?

**P02:** Ah...

**R:** Just as you are toward others, you were already treating yourself with compassion.

**P02:** I think so. Honestly, I don't get stressed that much. And even when I am stressed, I often don't realize it until it's over. When I was preparing for employment, I thought I wasn't struggling. But after it was over, my heart felt so light. "I guess I was struggling back then," I thought.

**R:** You’re naturally a "resilient" or "easygoing" person. That can happen. But these days, you notice the moment you are stressed and acknowledge it. I’m also a bit of an easygoing person... I’d go to the hair salon and they’d say, "Oh, you have a bald spot [from stress]!" Then I’d realize, "Oh, I guess I'm stressed." I was that type. But you're intentionally practicing "noticing" now. Listening to you, I really do feel your attitude toward yourself has become more compassionate. "So what if I spend that money? I want this, let's try it." Planning a budget so you can do what you want—that is an act of "caring" for yourself, which is ultimately self-compassion.

**R:** I feel a change in you. What do you think?

**P02:** That all counts as compassion? I see. I can think of those things as compassion too.

**R:** Indeed. This might sound like a leading question, so I'm a bit concerned, but "Compassion" (자비) is made of the characters for "love" (자) and "sorrow" (비). It means feeling sorrow for someone's suffering—loving them and wanting to pull them out of that suffering. So wishing compassion, acts of compassion... all those are compassion. So from my view, yes, it's compassion. You've always done that for others, but sometimes we can't do that for ourselves. It’s good. Please continue to take care of yourself first. One last question: Have you experienced any changes in your relationships—with yourself, professors, friends, family, or patients—through the program?

**P02:** I'm worried because I haven't met many people lately, but I feel that when you're stressed and struggling, you can't look at your surroundings. How can you look at others when it's hard to take care of yourself? So...

**P02:** I have students I tutor. They each have their own worries. One middle school girl has boy trouble and worries about her looks. She's so pretty to me, but she says she's ugly—she wants her eyes to be this way, her nose that way. I understand, because everyone goes through that at that age. But usually, I could only say the obvious things: "You're pretty as you are," etc. But what those kids really need is someone to listen. In the past, I might not have been able to do that well.

**P02:** If the stress is too much, I help them see it objectively. Before, I wouldn't have known what to say. For example, she's stressed because her nose is "ugly." I might have thought "Nothing you can do about it," but now I realize, "That's not objective, it's just what you think." To me, her nose is pretty, but to her, it's not. I acknowledge that first. But I can't let her stay stressed if she's thinking that way. So I can suggest solutions—like using "shading" (makeup). If she's satisfied, that’s it. It’s not that she's objectively ugly, but she *thinks* she is. So by doing makeup, she can...

**P02:** Get self-satisfaction. I think if she does makeup and gets less stressed, it helps her well-being. Her parents might not like it, but I understand because I had the same worries in my teens. I even shared some of my samples with her. "Do it well so you don't get caught," I said [laughs].

**R:** These days everyone wears makeup. Even boys.

**P02:** Yes, I see many boys doing it too. In places like Gangneung.

**R:** I heard in all-boys high schools, they talk while dabbing their compacts during breaks [laughs]. It’s a new world. Anyway, because you’re managing your own stress better now, you can understand their problems from *their* perspective, empathize, and even help with solutions. You’re actively involved, like it's your own business.

**P02:** My perspective on stress has changed. It's not that her nose is objectively ugly, but because she *thinks* so. I can't fix her nose, but I can help with the thought. That part was good. If stress is because you *think* you're stressed, then a solution can be found. Like my problem with spending money.

**R:** It’s a form of acknowledgment, first.

**R:** Instead of "Is that anything to be stressed about?", you say "It can be that way for you" or "for me." You acknowledge the stress itself, and then you look for ways to reduce it and increase happiness. How does that approach affect your relationships?

**P02:** The fact that someone trusts me enough to tell me their worries and talk to me helps my self-esteem and happiness. And in the relationship, they trust me more because we share sincerely and look for solutions. My relationship with my students is getting better.

**R:** Great. You’re a good teacher—thinking from the kids' perspective and worrying with them. Otherwise, it’s too easy to just give advice. They already hear advice everywhere. But you're in the same boat, worrying seriously, and even helping with makeup. You’ve become an "accomplice" against the parents, making you even more of a team.

**R:** It’s been over an hour! How are you feeling? Was it difficult?

**P02:** It's my first time doing this kind of research, but it was a good experience.

**R:** If I had given you the questions beforehand, it might have been easier to answer. I’m worried that thinking and answering on the spot was hard. How was it?

**P02:** No, it might have been more "compassionate" this way [laughs]. I think hearing the questions and answering immediately is fine.

**R:** If I gave them beforehand, you would have agonized over them too much. That makes sense. Well, we can stop here for today. If I have follow-up questions, I might contact you again, though that rarely happens.

**P02:** Thank you.

**R:** Thank you. Goodbye.

**Participant: P03
Date: 2024-04-02
Duration: 58 min 06 sec**
----------------------------------------------------------------------------------------------------------------

**R:** I’m recording this, and just in case, I’ll also use this voice recorder as well. Oh, I pressed "play" instead of "record," I was startled! I thought a ghost was talking [laughs]. Are you currently in a semester?

**P03:** No, I’m a graduate, so I’m waiting for my official placement.

**R:** I see. So when we were doing the program together, you were in your senior year...

**P03:** No, even then, I was waiting for graduation.

**R:** Right, waiting for graduation, and you probably had your graduation ceremony in the middle of the program.

**P03:** That's right. Yes.

**R:** And now, "waiting for placement" means you've been hired by a hospital and you're waiting for your start date. Has it been decided yet? When it is?

**P03:** No, not at all. Maybe because they are busy [due to the strike], they haven't contacted me. I just have to wait without a set date.

**R:** Gosh, it must be a mix of relief because you passed the hiring process, but also uncertainty because the date isn't set. You can't really start something new, but you can't just do nothing either. How do you feel in that state?

**P03:** I felt that way in February, but I heard they might call me very late. They said it could even be next year. So I was told to just wait comfortably, at least for the first half of the year.

**R:** I see. That can happen. So, are you just waiting? Or are you doing something else?

**P03:** I’m doing a part-time job and learning some things I wanted to learn.

**R:** It’s a period you can really enjoy as long as it’s not too vague. I’m glad you seem to have adjusted well. I sent the questions in advance, and I assume you’ve skimmed through them. Basically, I want to know what your experience with the MMPT program was like and what kind of impact it has had on your life. How about we start with your reason or purpose for joining the program?

**P03:** While "waiting," I was initially very anxious.

**P03:** It was around February, and I wanted to wait with a healthy mind, without being impatient. Also, I knew I would be under a lot of stress once I started clinical work, so I joined because I wanted to learn ways to adapt well to that situation.

**R:** So, during the wait... when was your hiring decided? Usually during summer break?

**P03:** It was decided in the second half of last year. Back then, I thought they might call me as early as March, so by February, I was getting impatient, wondering "When on earth will they call?"

**R:** So you expected March, and as February came, the impatience of waiting started to become difficult. You wanted to regulate that. And you've heard how hard actual work is, right? So you wanted to do "stress management" to prepare for that. Was it helpful?

**P03:** Yes, I think I learned several different methods.

**R:** For example, what kind of methods were helpful and in what way?

**P03:** I mentioned this at the very beginning [of the program], but I had a tendency to eat [stress eating]. But after learning Body Scan and things like that... usually, when I eat, I watch TV at the same time, so it feels like neither activity ever ends. I just keep watching and keep eating. But I changed it to "just focus on eating." By focusing more on the senses, like the taste, I can say "Eating is done" and move on to the next step. My daily routine has changed in that way.

**R:** So you use "Eating Meditation" when you're stressed. What does it mean to "move on to the next step" in your daily life?

**P03:** If I want to watch TV, instead of doing it together, I finish eating and then say, "Now let's watch one drama episode." It feels like a clean break. Or after eating, I do the dishes and then move on to what I need to do. Before, eating while watching TV made me lose track of time and just extend those behaviors. Now, it feels like the segments of my day are clearly divided because I focus on the senses and finish.

**R:** Does that help with stress regulation?

**P03:** Yes. I’m the type who gets stressed by myself if I eat too much, so gaining a sense of control over that was [beneficial].

**R:** Instead of overeating, you eat just enough and stop.

**P03:** Yes.

**R:** And then you move on to other tasks. Usually, people eat because they are stressed. When you do Eating Meditation, what happens to that high level of stress?

**P03:** What happens to the stress once it's mitigated?

**R:** Well, you said you turn to eating when stress is high. You decided to do it as "Eating Meditation." Then...

**P03:** Now, when I’m stressed, I don’t fall into [mindless] eating. If I feel anxious, I do Body Scan—that’s what I do most often. I focus on what sensations I’m feeling, or I go outside for a walk to relieve the stress.

**R:** How is that? You say it relieves the stress?

**P03:** It relieves that feeling of being "stifled." When I focus only on walking, sometimes my tangled thoughts get organized. It feels like I untangle the knots and come back. Before, I would just leave the knots as they were and start eating. The stress would still be there; I’d forget it while eating, but once I finished, the stress would double. That’s how it felt.

**R:** So doing Body Scan or walking doesn't mean you are avoiding thoughts; rather, it has the effect of helping you "untangle" them. Is there anything else?

**P03:** I thought a lot about the "Well-being Cognition" part. You said to use specific phrases and look for ones that fit. I couldn't find one back then, so I kept thinking about what to use. Later on... I value regulation and control very much. So, when I don't want to do something but it's actually aligned with my needs, I repeat this sentence to myself: "If I do it, it works. I can do it. I must do it."

**R:** Do you intentionally write that down somewhere? Or do you whisper it in your mind?

**P03:** I keep whispering it in my mind regarding the situation.

**R:** I see. When you have tasks or assignments ahead of you, it’s burdensome. But you first "notice" that "This is important to me, this is something I want."

**P03:** Yes, yes.

**R:** Then, by whispering "If I do it, it works; I can do it; I must do it," even if it's hard, what kind of impact does that have on you?

**P03:** Even if I don't want to do it, that mindset "covers" the reluctance and gives me the "execution power" to just start. Once it's finished, since it was something I had to do and something important to me, I feel a sense of pride. Like, "I did well."

**R:** You acknowledge yourself, saying "I did well." Actually, we already use Well-being Cognition in daily life.

**P03:** Yes.

**R:** But we encourage using it more actively and finding what specifically works for you. We suggest using it intentionally and proactively. Do you feel like you've been practicing this intentionally because of the program?

**P03:** Yes, completely intentionally. I didn't have this before, but I looked for it, and because whispering this sentence worked, I’m planning to look for more. I’m trying to think of other sentences or images for different situations. It's honestly completely intentional.

**R:** Right, intentional is good. If you keep doing it intentionally, at some point, it might naturally become a part of you. Is there anything else? You said you've been practicing the skills you learned.

**P03:** There was a question about whether I learned anything new about myself. Through that time of "looking into myself," I looked at what I really wanted for myself. First was what I mentioned—living while regulating and controlling my needs/desires. Second was "finding things I like." I hoped I would find them. So, since I have a lot of time now, I’m doing a part-time job at a cafe, which I really wanted to try. Making drinks, talking with colleagues, interacting with customers... I needed that time and really wanted to do it, so I'm doing that. Also, I was interested in hairdressing before, so I'm learning "hobby hairdressing." And I thought I should do some English while I have time, so I'm doing some English conversation. These actions are efforts to find what I like, right? So I consider them "Well-being Behaviors."

**P03:** I think of them as Well-being Behaviors to satisfy the "Motivation for Competence." I'm focusing more on what I'm doing and trying to keep it up for a set period.

**R:** So you reflected on "What do I want?"—we did that "Four-set of Wishes" exercise too. You reflected on your needs. And you realized that you are someone for whom the "Need for Competence" is important. So you are actively performing Well-being Behaviors by learning and trying out things you like, especially since you have the time. It seems like a great opportunity. If you were busy, you might not have been able to. How does actively seeking and doing these Well-being Behaviors affect your life?

**P03:** I used to always prioritize what I *had* to do over what I *liked*, which is why I couldn't find what I liked. But as I recognized and did things I like, the way time feels changed. Before, I’d think "A month has already passed. How will I get through the next month?" But now, my mindset has changed to "I've done this for a month but I still don't know it well enough. I'm excited for the next month." Doing the behaviors I like definitely brings more joy to my daily life, and I feel there are more things to be grateful for and happy about.

**R:** That’s amazing. In a way, the objective reality of your daily life might not be that different. But while you used to focus on what you *had* to do, now you’ve inserted things you *like*. Even though a cafe job can be tiring and learning English isn't always "fun" in a traditional sense, because you say "I'm doing this because I like it, because I want to," it comes across as something exciting and happy. Since I keep asking similar questions, is there anything else? Anything that impacts your life or yourself?

**P03:** Before, if I had time, I only spent it on "input." I just filled my time. But now, when I walk, do the dishes, or wash up, I do "Action Meditation." I don't fill that time with something; I empty my head or organize my thoughts. It gives me a sense of liberation. It feels like more empty space is being created in my mind and heart, which gives me the room to accept other things.

**R:** What did you used to fill that time with?

**P03:** I wouldn't just stay still; I’d constantly be listening to music, reading a book... not a time for organizing, but a time where I was constantly being filled from the outside. Or I was doing things I *had* to do.

**P03:** Now, if there’s an empty moment, I try to stay "blank" (spaced out) and not think, or I try to organize my thoughts. I put effort into having that kind of time.

**R:** Before, it was "input, input, input"—music, TV, or books. Now, you empty or organize more. What’s the benefit of that? To someone who hasn't experienced it, it might just seem boring.

**P03:** But you actually "secure" more time. When you move from time "soaked in dopamine" to time spent just being blank, time flows very slowly, relatively speaking. So it feels like I have more time. Also, because there’s more room in my heart, I have the capacity to accept things—a sort of "receptivity"—and I feel more at ease.

**R:** "Receptivity"—for example, what are you able to accept more of?

**P03:** I can accept what others say more, accept others' opinions more, and accept their emotions more. It feels like an "extra space" has been created in my heart.

**R:** Good. While we're at it, is there anything else?

**P03:** No, that's it.

**R:** Does your face hurt? [Noticing the ice pack]

**P03:** I had a wisdom tooth pulled, so it's swollen. They told me to keep an ice pack on it.

**R:** Did you have it pulled today?

**P03:** I had it pulled yesterday.

**R:** I see.

**P03:** Last week was fine, but this week’s tooth was a struggle to get out, so it’s a bit swollen.

**R:** You’re pulling your wisdom teeth a bit late. Usually, people do it in high school. They say it’s better to pull them.

**P03:** They said they were already decayed because of poor management. That's why I had no choice but to pull them.

**R:** If you had managed them well, you might not have had to pull them. That’s a shame. But thanks to that, I hope you become even prettier—maybe your jawline will get sharper!

**P03:** I feel good about it.

**R:** Good. You’ve touched on this already, but let me ask more formally: What kind of changes have there been in the way you perceive and react to stressful events?

**P03:** I've been going to the dentist a lot lately, and there were many anxiety-inducing situations. I didn't want to go. But like "Well-being Cognition," I kept reminding myself, "It's something that has to be done anyway," and I went. And it was really anxiety-inducing... last week it took 5 minutes to pull, but this week it took 20 minutes. I was honestly so scared. But the only things I could do were focus on sensations, focus on my shaking body, and "trust the doctor." Those three were all I could do. Instead of letting my thoughts spin toward bad situations, I focused on sensations and soothed my heart. It was bearable.

**R:** So you didn't get more anxious or shaky—well, you were anxious, but you soothed yourself and, above all, focused on sensations so you wouldn't be dragged away by your thoughts. That’s good. When I’m at the dentist, I listen to the clattering sounds... there’s nothing else you can do with your eyes closed. So you felt the body sensations? You heard a lot of sounds too.

**P03:** Yes, the sounds can be very scary. But I thought, "There's that sound, so I should try to open my mouth wider," and then I focused back on the sound.

**R:** Right, focusing on the sound. Is there anything else? Any other changes in how you perceive and react to stress?

**P03:** When I feel those sensations, you told us to try to look at them from the "outside." So instead of being buried in the emotion, I say "I am feeling this way" or "I am thinking this way," and I organize it. Instead of digging into negative thoughts from there, I just note "I am having this thought."

**R:** You’re doing that. That is a very difficult skill—we call it "Mindfulness." It means stepping back and seeing "I am anxious right now," "I am having this thought," "I want to do this."

**P03:** And then I add, "Is this true?"

**R:** What does that mean—"Is this true?"

**P03:** "Am I overthinking this?" Even if it's an emotion I'm feeling, "Am I perceiving the situation in a distorted way because of this uncomfortable emotion?" I add that question to "doubt" [the thought] one more time.

**R:** That sounds a bit like Well-being Cognition. How does your heart feel when you do that?

**P03:** If I start with a negative thought, I can organize my thoughts by saying, "It might not be true. I might just be feeling it more intensely because I'm uncomfortable with the previous situation." Usually, I’d be the only one getting hit by all the "arrows" of my thoughts, but now I can dodge a few of them.

**R:** Great. It seems like you're using a "hybrid" approach. Not just sensations, or just mindfulness, or just Well-being Cognition—I feel like you're using all of them together. You first "notice" that you are under a lot of stress, then you note "I am anxious" or "I am thinking this," and then you say "Let's focus on sensations, let's feel the body, let's listen to the sounds." And at the same time, you use Well-being Cognition—the words you say to yourself—like "Is this true?" or "Trust the doctor." You're mixing them appropriately. What would it have been like in the past? In that situation, lying there for 20 minutes with the sound of the drill and the smell of disinfectant?

**P03:** When I heard the sound... the doctor said they were carving the tooth into pieces. In my head, I would have been imagining the tooth being carved. "How is it being carved? How is my gum being ripped? How much blood is there?" I would have only had those bad thoughts. My fists would have been clenched tighter and tighter...

**R:** Probably. If you kept having those thoughts, how would your heart have been?

**P03:** I would have been constantly anxious. I used to think that hearing my heart beat made me even more nervous. I would have tried to "avoid" the body sensations entirely, kept my fists clenched, and my heart would have beat faster. I wouldn't have known because I was trying not to feel it, but my body would have been tense, my jaw wouldn't have opened well, and it might have taken longer than 20 minutes.

**R:** That's true. Since you were rigid and tense... the dentist usually keeps saying "Relax, relax."

**P03:** My hands were very relaxed, so I thought I was being comfortable, but they kept saying "Lower your head." I guess I was unconsciously lifting my head [from tension].

**R:** I see. This time, did you feel your heart too? In the past, you said you avoided feeling your heartbeat because it made you more anxious. This time, did you actually observe "My heart is beating like this" or "My body is tensing up"?

**P03:** Yes, I think I look at that first now. Even before the doctor came, while I was lying there, I was listening to my heartbeat.

**R:** And how was it? Did you get more anxious? No change? Or did you become calmer?

**P03:** I didn't get more anxious. Rather, I thought, "I am this anxious. Okay, this is fine." It wasn't beating faster and faster... it was just faster than usual. "Thump, thump, thump." Honestly, because I was so focused on that, I didn't really know if I was getting less or more anxious. I was just like, "Thump, thump, thump."

**R:** You were just quietly feeling it. That's good. By doing that, you didn't judge if it was anxious or not... it sounds to me like you "accepted" your anxiety: "It's okay to be anxious." What do you think?

**P03:** Exactly. "It's acceptable. It's acceptable to be this anxious in this situation."

**R:** Right, right. It is an anxious situation. The dentist is the scariest! You might even misunderstand and think you "love" the dentist because your heart is fluttering so much [laughs]. You mentioned you joined to prepare for the high stress of clinical nursing work. Now that you've participated, what kind of influence do you expect it will have?

**P03:** Everyone will be in a sensitive state, so I’ll try to "notice" that situation first. When patients or colleagues say harsh words to me, I'll think, "That person is in a sensitive situation right now. They don't hate me or dislike my personality; it's just that the words are coming out harsh because of the situation." I think that will be the first big help. Also, when I’m anxious in a chaotic situation, instead of "firing back," I’ll think like that, and then soothe myself by recognizing my physical changes. Also, you told us to "separate work and private life." I'll try hard to leave hospital matters at the hospital. At home, I'll try to focus on my actions—eating, washing up—to live my daily life.

**R:** How do you feel, having those "countermeasures" (strategies)?

**P03:** Since I haven't been doing clinicals, I skipped those questions on the sheet. But as you ask me to imagine it, I’m just telling you what I’m currently using, and it feels like it will be much more bearable. Instead of taking in 100% of the stress, I’ll take in much less. Since you said it depends on the situation and how I accept it, I think I’ll be more "flexible" in how I accept things.

**R:** So it sounds like you’ll first "observe" yourself. "I am feeling anxious right now"—you'll see that first.

**P03:** Because that's what the situation will be.

**R:** Right. Anxious situations will happen, and there will inevitably be people who say hurtful things to you. As you said, it's a place with many sick people, a sensitive place where life and death are on the line. Sharp words can fly around. In those moments, what does it mean? Does it mean you "care for yourself first"? Or does it mean you "empathize with them first" (put yourself in their shoes)?

**P03:** It means "keeping in mind" that they are in a sensitive situation. And when I'm in that situation, I'll take care of my own emotions and sensations first.

**R:** I see. If you do that, what kind of impact will it have on you?

**P03:** Instead of misunderstanding or distorting things, I'll first pull myself together. Then I'll look for how I should help them, or if it was a conflict, how to untangle it. Instead of just cowering or being intimidated.

**R:** Yes, you'll be able to "choose" a helpful action. It won't be easy, but it’s all "training." We say these are all "skills." It won't be easy, but as you practice, I’m sure you’ll do great. Because, we did this for 6 weeks, right? Listening to you, it seems you worked really hard on the assignments. I felt a bit sorry about giving "assignments"—I worried about giving "homework" to people already living hard lives. How was that? Doing the assignments?

**P03:** But I had to try it out to "embody" it.

**R:** Yes.

**P03:** By practicing what I learned in those 3 weeks [of training], I could think about how to apply it next time. If it had just ended with the lesson, I would have forgotten everything. It was a way to remember even a little bit more.

**R:** That's good. Were there any difficulties or things you felt were missing?

**P03:** During the 4th session when we learned Well-being Behaviors... maybe because we were short on time, but when we did the "7 types of motivations/needs," we moved too fast. So the meaning of each motivation wasn't well-established in my mind. I thought it would be easier to understand later if examples were included, even in the PPT we receive.

**R:** You found that missing. Categorizing things into these motivations was meaningful to you?

**P03:** I wanted to look into what each one was and create many Well-being Behaviors. But while doing it, I wondered "Is this right?" I wasn't sure if I understood it correctly while writing down examples.

**R:** I see. That makes sense. Accurately distinguishing them is important, but the act of "knowing" or "trying to know" is also important. If we had more time... our time was a bit short. I regret that too. If we had more time, we could have looked for all of them and shared them, and you could have reflected more deeply on your own motivations and needs. But we couldn't go into detail because of the time limit. Is there anything else that was hard or missing?

**P03:** I think everything else was fine.

**R:** Any difficulties while doing the assignments? Either the homework itself or practicing in daily life.

**P03:** At the beginning...

**R:** Yes.

**P03:** You mean the "circle-checking" thing? The checklist.

**R:** Checking the list might have been hard, or actually performing the tasks in daily life... or maybe nothing was hard.

**P03:** When I first saw the list, there were so many items! But after only one week, we only knew a tiny bit, so I was like, "What is this?" I wondered how I was supposed to understand and check the items further down. Honestly, in the first week, I printed it out. And in the first week, I felt like I did the top items and also the "Well-being Cognition" stuff... and even though I didn't know "Breathing Meditation," I thought, "Well, I'm breathing," so I checked it off [laughs]. If you had told us in the first week that the list follows the weekly schedule, I would have just left it and focused only on the first week's parts. It was only bad in the first week. I think you told us in the following week.

**R:** I see. Next time, I should use colors to separate "Week 1 items," "Week 2 items," etc. on the table. That would be a good way. What were the good parts of our program?

**P03:** I liked that it was online. I had already moved back to my hometown. If it had been offline in Seoul, I would have wanted to join but wouldn't have been able to. So personally, the online format was good. And honestly, making each person [speak/share]... it’s very burdensome before it’s your turn, but because of that, I felt more nervous and focused more. I got to tell my story and hear others' stories. I think I did that a lot more.

**R:** It's a relief to hear you liked the reflection/sharing time. I actually do that with a bit of a trembling heart, fearing I’m giving you a burden. But my intention was for us to learn from each other while listening to others, so hearing that you liked it makes me feel relieved. And it's good that the online format worked for you. What you are using now—even after the program—is "Action Meditation." But what about Breathing, Yoga, or Body Scan? You mentioned Body Scan.

**P03:** Yes, Body Scan.

**R:** Do you do Breathing Meditation too? Breathing, Yoga, Body Scan...

**P03:** I don't think I do Breathing or Yoga meditation. And I don't do Gratitude Practice or Self-Compassion either. Those are things I want to try and add in the future.

**R:** How do you plan to do Gratitude and Self-Compassion?

**P03:** I think doing it before bed is a good way to look back on the day. Before sleeping, I want to think about what I was grateful for during the day. For Self-Compassion, I’ll use the "Geon-Pyeong-Haeng-Seong" (Health-Peace-Happiness-Prosperity) phrases we learned.

**R:** So before bed, wishing well for yourself or others, and reflecting on things you were grateful for. We did things like "What was good," "What was grateful," or "Good things that happened to others"—basically all the "Well-being" things. Instead of writing them down, you’ll just think of them in your mind. And then finish by doing the Compassion practice—which is a type of prayer or wish. You want to wrap up your day like that.

**P03:** Yes.

**R:** That’s great. You want to try those. And you’re already doing Well-being Behaviors and actively using Well-being Cognition. How exactly do you do Body Scan?

**P03:** Is "feeling sensations" different from Body Scan?

**R:** Well, "Sensory Meditation" is... no, Body Scan is also a type of that. Feeling how your body is. You don't set a specific time for it, right? I mean, not "formally" feeling from head to toe, but just briefly feeling body sensations in the moment?

**P03:** Yes.

**R:** I see.

**P03:** I do it when I feel a significant change in my body sensations.

**R:** Right. Formal meditation isn't easy to do.

**P03:** No, it’s not.

**R:** You have to "strike a pose" and set aside time, so even if it's short, it's not easy. It seems you are using it by "inserting" it into your life in short bursts here and there. That’s how we practiced—teaching things you can insert into your life. What if we had *only* done formal meditation? How would that have been?

**P03:** "Striking a pose" and meditating for a set time... I am interested in that too. But it wouldn't have been "new." I know it's a time to relax the mind, close your eyes, and focus on breathing or sensations.

**R:** Yes.

**P03:** But being able to learn various ways to regulate stress was much more beneficial. I learned a lot of really "different" (unique) things. The terminology was very new too.

**R:** So doing various things instead of just meditation was more interesting and had higher utility for you?

**P03:** Just looking at what I use in daily life, there are many more of the "other" (non-meditation) types.

**R:** I’m glad. I was worried you’d find it hard because there was too much. In a way, having more "tools" is better. If you have many tools, you can use this one here and that one there. Did you learn anything new about yourself or change in any way since the program? You already answered that yourself earlier... is there anything else you’d like to add?

**P03:** I think I’ve said it all.

**R:** Good. This might be the last question: Through the program, has there been any change in your relationship with *yourself*—since we also have a relationship with ourselves—or in your relationships with others, like professors, friends, family, or patients?

**P03:** Like I said, after "emptying" a lot, I feel like I have more time for others, and my heart has become more "roomy" (generous). When I talk with my family, I can listen more and I’ve changed toward being more "receptive" to their emotions.

**P03:** It’s not like we’ve become "dramatically" harmonious, but there are small changes. My emotions have moved in a positive, harmonious direction.

**R:** You mentioned being more "receptive" to others' emotions because you have more room. What do you mean by "receptive"?

**P03:** For example, someone sees a situation and gets annoyed. I might not understand why they are annoyed by that. But instead of my own standards, I try to understand from their standards. That makes their annoyance "receptive" (understandable/acceptable).

**R:** To me, that sounds like "putting yourself in their shoes" (empathy). Is that right?

**P03:** I think so. Even if it's not about me, I can accept how that person views an external situation.

**R:** I see. Does that happen for *yourself* too?

**P03:** Yes. I’ve come to know my sensations, emotions, and needs much more. I definitely know myself better than before. Changing the way I regulate stress, gaining more methods... it was that kind of time.

**R:** So you know your emotions, your needs, and your state. What is that like? It sounds to me like you're not just "knowing" them, but becoming more "accepting" of them. Is my understanding correct?

**P03:** Yes, yes. I think I can accept those things.

**R:** What was your tendency in the past?

**P03:** In the past, I frequently "ignored" or "avoided" sensations unconsciously. That frequency has decreased, and "acceptance" or "embracing" has increased.

**R:** What is the result of that "acceptance"?

**P03:** Once I accept it, it ends more easily. If I ignore it, it grows like a "lump" or a "mass." But if I accept, acknowledge, and permit it, it ends at that size. The emotion doesn't explode; it feels like it just "scatters."

**R:** That’s amazing. I should do that actively too. I’ve been having a hard time lately [laughs]. I feel motivated to acknowledge myself: "Oh, you're having a hard time." P03, you’re amazing. Was this your first qualitative research interview?

**P03:** Yes, it’s my first time participating in qualitative research.

**R:** Have you participated in experimental research before?

**P03:** No. This is my first time participating in research at all. I’ve participated in programs before, but never a program for research purposes.

**R:** I see. How was the interview?

**P03:** A lot of things came out that I hadn't thought of. Especially the question about "How do you expect it to be in clinical practice?" helped me organize my thoughts. It made me look back at all 6 sessions. It feels like a good "period" (full stop) at the end. And I was surprised that we actually filled an hour!

**R:** Right? When you just look at the questions, you might think, "This won't even take 10 minutes."

**P03:** I thought it would end quickly.

**R:** But as we talked, an hour was filled. That was a new realization for you. You spoke so delicately and well, it was very interesting for me too. The hour flew by. I feel you’ve observed and understood yourself very well, which helped me understand you better too. I think we should wrap up now. If I need follow-up questions, I might contact you again.

**P03:** All right. Yes, yes.

**R:** Thank you so much for doing so well today. Goodbye!

**P03:** Yes, thank you.

**Participant: P04
Date: 2024-04-07
Duration: 50 min 22 sec**
----------------------------------------------------------------------------------------------------------------

**R:** It’s okay to record this, right?

**P04:** Oh, yes, yes.

**R:** And just in case—this is precious data. Since our interview is research material, I’ll record the call as well.

**P04:** Okay. [Jokingly] "I hate being video recorded!" just kidding.

**R:** Video? But I’ll delete the video. I’ll do it as soon as we're done. This tool doesn't record only audio, but I don't look at the video anyway. I just take the audio and convert it into text. Then I analyze that. I don’t present any information that could identify the individual. I’m not sure if you’ve read qualitative research...

**P04:** I know what it is. Yes, I know.

**R:** You do? "Participant 1 said this and that"—it’s at that level. Good. I should have sent the questionnaire beforehand, but I realized I forgot. This is how I work [laughs].

**P04:** If you send it now, I’ll take a look. The questions.

**R:** That's fine. I can just ask you. It doesn't matter. You can just speak comfortably and honestly. I’ll ask follow-up questions for details if needed. So, you can just say whatever comes to mind comfortably and honestly. The first question is about the MMPT program you participated in. Do you have a reason or purpose for participating in the MMPT program?

**P04:** The reason was that it’s related to stress relief. Nursing students get a lot of stress, right? During the semester or when doing assignments. So I applied because I thought it would be helpful.

**R:** So you wanted to get help with managing stress, and that was both your reason and your purpose.

**P04:** Yes.

**R:** How do you feel? Do you feel like that goal was achieved?

**P04:** It worked well during the vacation, but during the semester, I’m so busy that it doesn’t seem to work well. When the program was ongoing, we had the weekly Zoom sessions and participated, right? There were missions and assignments to perform, and we did it regularly every week, so it went well then. But now there’s nothing like that, and I’m so busy that I don’t have much room in my heart. Maybe that’s why I feel distant from meditation.

**R:** Oh dear, are you very busy because of your clinical practicum?

**P04:** Well, even if it’s not clinicals, the current one ends next week. On the weekend, there are quizzes and assignments right away. And even on weekends... I have an academic conference this week, and an image interview clinic next week. Then there are more assignments and lectures to listen to. Since the semester started, it’s been a non-stop rush.

**P04:** I’m a senior (4th year), you know. So I’m really busy and a bit out of it.

**R:** There's so much to do. You have to do clinicals and the classes are compressed into a short period, right?

**P04:** Yes. 2 weeks of clinicals, then 2 weeks of class, but those 2 weeks cover a whole month's worth of material. So it's very tight.

**R:** And right after that, midterms. I see. Since it’s so tight, you can’t help but have a lot of stress. You participated because you wanted help with managing that. You participated for a month and a half, for 6 weeks, without missing a single day. After that, was there a change in yourself? If so, what kind of change was it?

**P04:** While I was doing it, how should I put it... we do those meditations, right? When emotions or thoughts arise, I can shift them a little through various methods. When a feeling comes up, I’d say, "Oh, so that’s how I felt," and I used sensory meditation and other things. I think it was very helpful for me. When I’m anxious or stressed, it helped me not to fall too deeply into those thoughts.

**R:** So it helped prevent you from getting stuck in your thoughts when you're anxious?

**P04:** Yes. Especially when showering—even now, I keep thinking about what to do. "What should I do? What should I do?" If it's evening, I think "What should I do tomorrow?" or "What did I do today?" But while doing that [meditation during shower], I had a goal, so I could think, "This is warm," or "This smells good." It worked well. By doing that, my thoughts move to the senses, so I can consciously stop thinking. I liked that.

**R:** So back then, you could stop thinking and reduce anxiety. But you said your purpose for participating was to use this during the semester when clinicals are hard. You already know the techniques. There must be a reason why you aren't using them now.

**P04:** I think it’s because there is so much to do that I don’t have any room in my heart. Even in those spare moments, I keep thinking, "I have to do this, I have to do that." Meditation doesn't actually take that long, but I’m not doing it well. Even from Thursday to Saturday, I was so anxious and restless... I felt like I had to do something, but I couldn't do anything, and I just stayed in that state. That made me upset. It went so well during the vacation, but now I don't know what to do. I’m under so much stress.

**R:** But during the vacation, you actually had even bigger stressors back then.

**P04:** Yes, but honestly, it’s the same now as it was then. To be honest, it’s been very hard for me. Friday and Saturday were just... I wanted to do something, but it didn't work out. I don't know why.

**R:** Back during the vacation, you were in the middle of the program. Even though you had hard times then, what did you try doing?

**P04:** Back then, I think I made a very conscious effort to do those things. Lots of meditation. After 2 or 3 weeks from the start, I was actually in a pretty good state. Maybe that’s why. But now, it feels like I’ve returned to how I was when I was first struggling. I want to do something, but it’s like I’ve forgotten everything. That’s the state I’m in.

**R:** It seems like things got busy almost as soon as the program ended.

**P04:** Yes. The semester probably started that same week. It was the first week of March. It lined up perfectly.

**R:** That’s a shame. The program ended and a new semester started right away. Timing-wise, it seemed perfect to use what you learned in real life, but it didn't lead to that. Listening to you, I feel a bit of regret. If something had been different, do you think it would have continued?

**P04:** When I applied, I obviously intended to use it during the semester. If the program had been *during* the semester, I wouldn't have even been able to apply. But still, if there was a meeting once every two weeks... two hours is too long, but even 30 minutes or an hour of sharing, or even just the teacher asking me for 10 minutes if I did the task—I think I would have worked harder at it consciously.

**R:** So, if there was someone to check in on you, or a device to keep you in check, you would have done it steadily. You’re saying it's hard to do it autonomously on your own?

**P04:** Yes. If you gave us a mission like, "Shall we try yoga meditation this week?", I think I would have done it more. Of course, I wouldn't be able to do it during exam periods. But even if I couldn't do it every day, I think I would have tried. Right now, I’m so out of it that even when I try to do more, it doesn't work. That made me quite upset.

**R:** I see. So after MMPT ended, are there no techniques you are practicing or using in your daily life?

**P04:** I think there was one. I don't remember clearly, but during my Psychiatric Clinicals, you told us to step back and observe our emotions like a CCTV. "Oh, so that's how it was." During the first week or two of the semester when I was doing psych clinicals, I think I had more room. Because then I had to be there by 8:00 AM, but now I have to go by 5:30 AM [implies earlier shift], so I have no time. I don’t remember exactly what happened, but I remember going to work saying to myself, "You were feeling this way," or "You were upset." I remember feeling a bit better. I try to do that occasionally when I remember. Like patting myself on the back and saying, "You felt this way."

**R:** Are you still using that from time to time?

**P04:** Yes, I'm trying to do that a little. But I don't know why that "Shower Sensory Meditation," which I did the most, isn't working. It was the one I did most often, it was the easiest, and for me, it had the best effect. Why is it not working well?

**R:** Maybe you're too busy getting ready for work in the morning. Perhaps the pressure to wake up and go quickly...

**P04:** But I usually shower in the evening, so I should have more room then, but it still doesn't work well, which makes me upset. I really tried to do it. Especially from Thursday to Sunday, it was so hard. But it just didn't work. I wonder if it's because I'm under too much stress. I want to do it.

**R:** I see. But besides showering... it’s been about 4 weeks since the program ended. Have you been doing it steadily during those 4 weeks?

**P04:** No, I haven't been able to do it steadily. I do it whenever I think of it, but not every day. Saying "Oh, so that's how it was"—I do that, but honestly, I haven't been paying so much attention to this program that I don't even know if I did it or not. But I do remember that when I *tried* to do it, it didn't work well.

**R:** You're referring to the Shower Meditation.

**P04:** Yes.

**R:** This is also a skill, so it probably worked well when you were practicing it intensely. But it’s been 4 weeks since it ended. You haven't done it for 4 weeks because you were busy—you had your reasons—so perhaps the skill you built up has weakened. Just like with yoga, if you don't do it for a month, things you could do before don't work as well. Also, meditation works better when the mind is at ease.

**P04:** Yes.

**R:** But when the mind is struggling, thoughts increase, so it’s naturally harder.

**P04:** I think that’s it. During the vacation, my mind was actually very much at ease.

**R:** And you did it steadily then. But because you were busy and stopped doing it, your "skill level" dropped, and then you suddenly tried to perform at a "high difficulty" level...

**P04:** That must be it. Truly.

**R:** So it is a bit of a shame, but...

**P04:** I really need it, so I tried very hard to use it.

**R:** You did? I see. That’s okay. Life goes on, and this isn't the end.

**P04:** I did do that one thing. Even today, since I was a bit upset, I said, "You were quite upset."

**R:** You must have been very upset.

**P04:** [Discussing a recent specific stressor] I just endured and endured, being very careful, and I reached out gently, but when the reaction came back so cold, I felt very upset and disappointed. The weather is so nice lately, which makes me think of it even more...

**R:** It's okay. You can just try again next time.

**P04:** Oh, so... when you go to clinicals and it’s hard, or various thoughts come up, you observe your mind from a distance. You've done that steadily. But the meditations you did a lot during the program, you haven't been able to do because you were busy. Is there anything else you are using? We actually covered many techniques together.

**P04:** Let's see... what else is there?

**P04:** Those two were my main ones. I used to do Breathing Meditation too, but since I can't sleep at all now, I don't think I have the chance to do it. I either fall asleep immediately because I'm exhausted or I can't sleep at all, so Breathing Meditation seems impossible. Walking, though—I walked twice today. When I walk, I try not to think. Like "The sky is pretty," you know? I try to focus on the senses. I haven't even had time to choose [different techniques], but I try to do those. And the "Gratitude" practice you taught us—when I’m feeling okay, I try to think about things I'm grateful for. There are times when it works well.

**R:** There are times when it works and times when it’s difficult. Especially when your mind is struggling, it’s harder. And since you're very busy lately, that's definitely a hindrance to steady practice.

**P04:** Yes, I think so.

**P04:** Are other people doing well even when they're busy? I'm curious.

**R:** The people I've interviewed so far weren't that busy. I recruited 3rd and 4th years...

**R:** So, you are the first person I'm interviewing who is truly having a hard time.

**P04:** Maybe everyone is similar? I think they'd be like me.

**R:** Well, the three people I interviewed before are spending the most relaxed time of their lives right now.

**P04:** Right, after the national exam. I’m jealous. I’m at my busiest. And as you know, with the doctor's strike, all the university hospitals aren't hiring new nurses, so it’s a complete emergency for us. Everyone is in a panic. Those people [graduates] probably won't even be able to start their jobs even if they got in.

**R:** They seemed anxious about how long the wait would be.

**P04:** Yes, that's right. That’s what I heard.

**R:** The next question: If there was a change in yourself, Jeong-hwa, through the MMPT program, what kind of change was it?

**P04:** You mean now? Or while I was doing it?

**R:** Both are fine.

**P04:** During the program, I think I tried very hard to do these things. It went well because it was vacation, but sadly, it doesn't seem to be practiced well in real life now.

**R:** Yes, yes.

**P04:** I really wanted to use it during the semester, which is why I applied during the vacation, but I’m a bit disappointed that I can't use it as much as I thought during the semester. Yes, that's right.

**R:** And the next question: What kind of change did you experience in the way you perceive and react to stressful events?

**P04:** Honestly, receiving stress feels the same. But like I said earlier, when I have certain feelings or emotions, I can now notice them and say, "So this is how I felt," and try to look at my emotions objectively. I think I’ve become able to do that. It doesn't mean I don't get stressed; if a situation happens, I get stressed just the same. It doesn't feel like I get *less* stress. It's the same, but I can comfort myself more. "So that's how it is." That part is different from before. I don't think I did that in the past.

**R:** How did you handle stress in the past?

**P04:** In the past, I didn't really have stressful situations like this. Usually, when I was stressed, I ate or slept. When there were many assignments and much to do, I was just restless. But a few days ago, that stress... I’m in a position where I have to wait for something. I was so anxious, restless, and under extreme stress. I couldn't do it right then, but I think I did it for the first time today. "So you felt very anxious. That can happen." Today I finally felt a bit better, so being able to do that for myself is a bit of a change.

**R:** Looking at yourself that way... it means looking at yourself objectively, seeing that you're feeling such emotions and struggling, right? Is it different when you look at yourself like that? In what way?

**P04:** It feels like I can think, "That can happen, it's okay anyway."

**P04:** How should I say it... it comforts me. It feels like I'm comforting myself.

**R:** A reaction where you comfort yourself. How does your heart feel when you accept and comfort yourself like that?

**P04:** I think it feels much better. By saying, "You were this anxious and sad. It's natural to feel that way," what should I call it? I feel that I'm not weird, and it's natural and normal to have such feelings. I think I’ve come to accept it more. Usually, people want to suppress emotions or stress, or they don't want to feel those things. But by saying "It's okay, it's still okay," it feels like the stress is mitigated a bit. It’s like, "It's okay to have those feelings, it's okay to be under such stress. That can happen."

**R:** That’s warm.

**P04:** I'm glad I could at least do this.

**R:** Of course. Life is hard enough; imagine how much harder it would be if you scolded yourself.

**P04:** I want to cut off those thoughts, but they keep coming back. In those moments, I don't really know what to do. It’s like I’ve forgotten everything I learned.

**R:** Later, when you have time, you can look at the "Card News" I sent you on KakaoTalk.

**P04:** The PPT stuff?

**R:** Yes. You can skim through those and use something that looks good.

**P04:** Okay, I'll try.

**R:** We are bound to forget. That’s why reminders are necessary. It’s not just your characteristic; everyone works that way. It’s even more so because you’re busy. Did MMPT have any impact on your clinical practicum?

**P04:** The impact was... when things were hard during clinicals, I looked at my emotions objectively, like "Oh, so that's how it was." Then, when I go to work, I can pull myself together and go with a lighter heart. Even if something hard happened yesterday, I can start today happily by letting those things go. I think it helped with that.

**R:** You must have done that often in the morning.

**P04:** When going to work... honestly, I was doing Psych clinicals, so it worked better. Like praying for the patients. Now that I'm in a general ward, it's a bit different. Psych wasn't physically taxing at all. So I think it was possible. I went to work by 8:00 AM and did programs with patients... the job was basically talking and "therapeutic communication." I think I could do it until then.

**R:** So in the morning, you noticed your state and encouraged yourself, and that helped your day go better.

**P04:** Yes, yes.

**R:** Is it hard these days?

**P04:** Yes. These days I have to wake up at 5:00 AM and go right away. And since it’s a hospital ward... I think it’s impossible.

**P04:** Honestly, at the hospital, waking up at 5:00 AM every day is hard, and my body is exhausted. Even though this clinical doesn't have many assignments, I personally have been under a lot of stress... so I kept thinking, "I should contact them then," and those thoughts made it harder. And I felt like I couldn't do anything, so I wanted to cut off my thoughts using these techniques, but it didn't work at all, which made me upset.

**R:** As you're speaking now, is there anything you wish you had tried?

**P04:** Maybe something like Walking Meditation. When I walk, thoughts often disappear. But currently, I keep thinking even while walking. If I did those meditations that use the body, wouldn't that have been good? But since there’s no time for that, it’s upsetting.

**R:** Really? One can't get through the day without walking.

**P04:** You mean like during my commute?

**R:** No, we walk all the time. I bet you walk constantly in the hospital.

**P04:** Yes, yes. But it's impossible in the hospital.

**R:** Really? Why?

**P04:** I have to be mindful of the patients and the other nurses. I never even thought about doing it in the hospital. I walk, of course. I walk the ward. Ah, I see. I always thought of "walking" as something like taking a stroll when I have time... but come to think of it, I used to do it during my commute.

**R:** Are you talking about during the MMPT program? Commuting then?

**P04:** I think it was okay until the Psych clinicals. When I had some room during the first two weeks.

**R:** Yes, you did Walking Meditation during your commute then.

**P04:** Yes, it worked then. Thinking back, it was quite...

**R:** I see. That’s excellent. Why do you think it doesn't work now? Your commute time is probably the same, just the clock time has changed.

**P04:** I do commute. Why doesn't it work? Honestly, I'm so sleepy now that even for a short while, I just want to look at vegetables [on a phone/market] or I just doze off on the bus. I never even thought of doing it. I guess I didn't have the mental room to think that far.

**R:** You’re very tired.

**P04:** Yes, I'm so tired but I can't sleep, so my brain isn't working properly right now.

**R:** I see. So when you weren't *that* tired, you did it.

**P04:** Now that you mention it, I remember. It must have been so relaxed and nice back then.

**R:** And in the morning, you noticed yourself and encouraged yourself, so your day went much better.

**P04:** Yes, thinking back, those two weeks I went to work feeling very joyful, happy, and grateful.

**R:** But since the 5:00 AM shifts started, you haven't been able to sleep, you're tired, so you let go of all these things.

**P04:** I guess so. Just mindlessly.

**R:** I see. If you were to try one thing again now, what would it be?

**P04:** What would be good? Breathing? Since I can't sleep well, maybe that would help? And even if it's short, walking would be good. I really liked meditating while showering. I'll try it again, but it didn't work well before.

**R:** Then do what works. If you like walking, do walking. Even inside the hospital ward, there are many moments of walking. When I see nurses, they are always running around.

**P04:** When walking in the ward for such a short time, what should I think about?

**P04:** No, you just need to feel the soles of your feet. It's not about "thinking." Meditation is about feeling sensations. Even during that short time, just feel the sensation of your soles, the sensation of your feet.

**P04:** When I walked before, I tried to feel nature. "The air is good, the sunlight is warm." But now it’s so early that it’s pitch black. And when I go, the sun might be up, but there’s no nature in the ward. So I didn't think to do it.

**R:** Meditation is about experiencing through sensations, so it doesn't have to be "good" things. Even a ward consists of all sorts of colors and shapes. And when you move quickly, you can feel the flow of air. That’s wind. You can feel that, or the temperature, or the contact of your clothes against your body. Countless sensations occur even during that short movement. You can feel your soles hitting the floor, the strength in your toes, or the sound of your footsteps. Your five senses are active. Just feeling the five senses as they are—that’s meditation. You can do that for a moment. Or even in a busy ward, you’ll go to the bathroom to wash your hands, right?

**P04:** Yes, I wash them a lot.

**R:** Then while standing there for a moment, use your five senses. Wouldn't that be a "spare moment" practice? If you try to do it too formally, you won't be able to. You're too busy and there’s too much pressure.

**P04:** You're right. I wash my hands so much at the hospital, but I never once thought of doing that.

**R:** It won't be easy because the pressure is high. But in those moments, if you empty your mind once, even if it's hard, wouldn't it be more helpful? Now I’ve suddenly switched from interviewer to teacher [laughs]. It’s okay. Were there any other difficult or disappointing parts?

**P04:** As I said, since there were no weekly Zoom sessions, I stopped doing it on my own. If I had more room, I might have done it, but because I’m busy and there was no "enforcement" or anything, I stopped. There were too many other things to do. And when I was under extreme stress, I couldn't do anything. That was a bit disappointing. But that’s exactly when I should have used what I learned, right?

**P04:** Actually, in the most necessary moments... I did do it. After Thursday, Friday, and Saturday passed, and I felt a bit better today, I said, "So that's how it was." The problem is that in the heat of the moment, I can't think of anything.

**R:** It would be great to use it in those moments, but you were in a very painful situation. In hospital terms, you were in the ER bleeding out. Someone who is a real expert or "master" might be able to do it easily even then, but as I see it, Jeong-hwa, you were in an "ER bleeding" state, so it couldn't have been easy. In a way, getting a "shot" [medical intervention] might have been better at that moment. But I'm glad you practiced it three days later. Were there any good parts of our program?

**P04:** During the 6 weeks?

**R:** During, after, the content—anything that comes to mind.

**P04:** During the program, I liked learning something every week, doing it together, and sharing. Learning new things every day was good. And you know, to keep it up steadily... but since everyone there was a stranger... if it was with friends, like sending a "proof shot" saying "I did this," that would have been good. For example, I have a close group at church and we do Bible study together. If someone says they prayed today, we send a prayer emoticon. If there was a meditation equivalent, we could send meditation emoticons. I think that would stimulate and help each other.

**R:** So you mean making a group chat and "verifying" your practice?

**P04:** Yes. Even if it's not "verification," if the teacher sends something every day like "Shall we try this today?" like a notification talk—that would be good. Even if I can't do it, someone is reminding me. Like a weather alarm. I think I could have done it more steadily then.

**R:** I see.

**P04:** Because it’s not familiar.

**R:** So having continuous reminders or alarms to help you practice would have been good.

**R:** Right. But we did give assignments, and I shared a Google Sheet. How did you perceive that?

**P04:** But I didn't record on the Google Sheet right after doing it. I’d record it later, like "Oh, I did this."

**R:** So you're saying a daily alarm to prompt you would be more helpful for practice?

**P04:** Yes.

**R:** Okay. I'll think about how to reflect that next time. During or after the MMPT program, did you learn anything new about yourself, or has your thinking about yourself changed?

**P04:** Something I learned about myself?

**R:** Yes, or if your thoughts about yourself have changed.

**P04:** These days, I’ve realized "I have a very quick temper/am very impatient." I learned that about myself. And changes... knowing that there is such a thing as meditation, I try to do it when I remember. I can't control all my emotions or stress like an expert who does it every day... I actually expected that when I applied. "Does this really help?" But I think that was my greed. How could that happen just by doing this for a short time? I’m not a master or a miracle worker.

**P04:** So I think I expected a "dramatic" change. I’ve realized now that it’s not like that.

**P04:** Expecting a dramatic change for anything you do doesn't really make sense.

**R:** To what extent did you want to regulate yourself?

**P04:** For instance, when something stressful happens, I think it's natural to feel sad, anxious, restless, or depressed. But at least to the point where I can sleep. To the point where it doesn't affect my life.

**P04:** Not being able to sleep for 3 days was so hard. My body ached, and my head was foggy. When you can't even think, how can you think about meditation? I wanted to be at a level where my "thinking" isn't blocked so that I can function in daily life and not say weird things because I can't think. It's a bit disappointing that I can't think [of meditation] in those moments...

**R:** Was that the "dramatic change" you initially aimed for?

**P04:** Yes. I wasn't expecting [to never feel] stress, but like I said at the start, when I have exams or assignments... when I first get an assignment, I get very restless. "What should I do? There's too much." Now I have even bigger stressors, so I don't feel "restless" [about assignments] anymore... but I wish I could have regulated that anxiety and restlessness better.

**R:** But you're saying the impact on those things was minimal?

**P04:** Yes.

**R:** It was difficult to experience significant change. What do you think?

**P04:** About dealing with stress?

**P04:** Yes. But this was an "extreme" stress that I didn't expect. Honestly, judging [the program] based on this situation doesn't feel right. It’s just that this unexpected thing happened right before the interview.

**R:** That's fine. What about restlessness regarding exams?

**P04:** Ah, when that time comes, it'll be less than this, so it might be okay.

**R:** I see. Were there any changes in your relationships? With professors, friends, family, or patients you meet at the hospital?

**P04:** I’m not sure about other people. But during the psych clinicals, those patients have hurting minds and aren't "normal." There are moments when they are nice and then suddenly curse or change strangely. If I didn't know [about these techniques], I might have been hurt. But I think I’ve come to understand them more. Should I say I’ve become more "generous"? "That can happen. Okay, you weren't feeling good because of this. That can happen. You are hurting, and I am someone who should understand you." I can do that.

**R:** When you say you became "generous," do you mean toward those people? Or toward your own "hurt feelings"?

**P04:** I think both. My understanding for them increased, and for myself too: "Just like yesterday and today, you were upset and sad." I like that. I’ve never done that for my own emotions before. You told us to "label" our emotions, right? That was interesting.

**P04:** It feels like I’m really patting myself on the back. Even if no one else knows, *I* know myself. So that’s good. I don’t think there were many changes in relationships with friends.

**R:** But you said you became more generous toward patients.

**P04:** Patients. Yes, I think I treat them differently.

**R:** Does "treating them differently" mean treating them more generously?

**P04:** Yes, I try to treat them like an angel.

**R:** An angel? You're not an angel, but you're trying to act like one. You're putting in a lot of effort. Acting like an angel is hard. It seems you are trying very hard. Does your clinical end this week?

**P04:** No, it ends on the 12th.

**R:** The 12th... so you're even going on the 10th (Election Day)?

**P04:** I don't go on Election Day. I only have 4 more days to go.

**R:** 4 more days. Then your first-semester clinicals will be over.

**P04:** No. There’s one more in May. After that, I have classes and midterms, and then I go back out in May.

**R:** After that, you’ll have to prepare for employment.

**P04:** Yes, and then finals.

**R:** Gosh. Will you be less busy during the summer vacation?

**P04:** Well, this part won't go into the [research] content, right? Originally, I was...

**R:** Wait, I’ll turn off the recording.

**P04:** Oh, okay.

**Participant: P05
Date: 2024-04-08
Duration: 66 min 17 sec**
----------------------------------------------------------------------------------------------------------------

**R:** Just in case, I’ll record this through this device as well. I shouldn't lose such precious data, so I'm staying sharp and recording in several places. You might conduct research yourself later, and then you'll understand how I feel [laughs]. Good. Until when is your current clinical practicum?

**P05:** It ends this week, next week, and the week after that.

**R:** So you'll be in clinicals almost until the end of April. You had classes in March, and now you’re doing clinicals in April.

**P05:** It was clinicals in March/April, and then classes in May/June.

**R:** I see. So you’re more than halfway through your clinicals now.

**P05:** Yes. Once April is over, I’m done.

**R:** Is this your third clinical placement?

**P05:** You mean for this year?

**R:** No, I mean in total since you became a nursing student.

**P05:** I’m not sure. I’ve rotated through so many hospitals that it’s probably not the third—maybe the fifth or sixth.

**R:** I see. And after finishing this in April, are you done? Or do you have more in the second semester?

**P05:** I have more in the second semester too.

**R:** You're working so hard. Okay, this is qualitative research. The main question is about your experience in the MMPT program—you might have forgotten the name—and exploring what impact it had on your life. First, what was the reason or purpose for your participation in the program?

**P05:** It was the winter break before my 4th year, right before job hunting. Since the research was about 'mindfulness,' I figured it was a period where I’d likely face stress from seeking employment. I didn't have much stress back then, but I thought it would happen in the future, so I wanted to prepare in advance through mindfulness. Also, I thought it would be a program that helps me mentally if I could set aside time once a week for myself. It was also the period for writing self-introductions, so I needed time to get to know myself. I participated thinking I could organize my strengths and inner thoughts through meditation.

**R:** So, the purposes can be summarized as: first, wanting to learn how to manage your mind for job hunting, and second, wanting to increase self-understanding. Anything else?

**P05:** Also, I lost my dog last July, so I was struggling mentally. Going to a psychiatrist felt a bit too "heavy," but I wanted some form of counseling or something like that.

**R:** How many years did the dog live?

**P05:** 15 years.

**R:** Your voice is trembling even now.

**P05:** Yes.

**R:** 15 years... that means you adopted it as a puppy and stayed together for 15 years before it passed.

**P05:** Yes.

**R:** That’s much more than half of your life. I see. These days people get counseling for "pet loss," but...

**P05:** I didn't specifically do anything like that. I’m okay now; it's been about 6 months.

**R:** I see. You participated with those three goals. Looking back, do you feel those goals were achieved to some extent?

**P05:** I can't tell by objective numbers, but having a regular time for meditation was good for me.

**R:** Good. So, through the program—practicing, training, and learning various techniques—what kind of change did you experience personally?

**P05:** Usually, I only did abdominal breathing and breathing meditation, but through the program, I learned theoretically that there are many different methods of meditation. I learned there are ways to meditate through sensations, like sensory meditation, or by focusing on specific thoughts, like compassion meditation. Learning the theory was helpful. It gave me a chance to try various types of meditation in the future.

**R:** Did you meditate before this?

**P05:** I’ve been doing yoga since middle school. In the beginning of yoga, you always close your eyes and breathe. I continued that through high school and college; we always did about 10 minutes of meditation during yoga class.

**R:** They give you time for meditation in yoga? Just Savasana?

**P05:** Yes, that too. Sometimes lying down, sometimes sitting at the start.

**R:** So they actually guide meditation there?

**P05:** Yes, I’ve had several teachers, and they all did it.

**R:** Really? Then what’s the difference between the meditation you did there and the breathing meditation in our program?

**P05:** There, it was mostly about inhaling deeply, holding it, and exhaling. Here, it was... I don't remember clearly, but it was more diverse and longer.

**R:** I see. Do you still do breathing meditation the way you learned it then?

**P05:** I prefer following what others tell me to do, so it's not like I choose what I like; I just follow the class or the time I'm in.

**R:** Do you still go to the yoga studio these days?

**P05:** Not lately. I can't do it during clinicals.

**R:** Because you're busy?

**P05:** Yes, but even when walking, I try to do abdominal breathing. While walking to my clinical site today, I focused on my breathing.

**R:** Focusing on the breath while walking, but doing it as abdominal breathing? Inhaling deeply, pausing, and exhaling slowly... so you use it while commuting?

**P05:** Yes. If I don't pay attention, I don't do abdominal breathing. I feel my breath gets deeper when I do it. Also, it makes me yawn. My eyes are very dry, but if I yawn, they feel moist. Since abdominal breathing always makes me yawn, I do it on purpose.

**R:** You said learning the theory in our program was unique. What did that mean to you?

**P05:** Usually, meditation is just about the practice. But here, there was an academic aspect to mindfulness—why we do it and how it helps. You taught me information I didn't know through the PPT. That was different. There were many theories and methods, and those felt new to me.

**R:** How did that "newness" impact you?

**P05:** For example, there was a lecture saying that if you try to stop thinking, you end up thinking more.

**P05:** I used to think that was just my own personal issue, but when you said it in the theory, I realized, "Oh, this is a normal thing, others are like this too." Also, I never thought about things like "compassion" or wishing for others' well-being before...

**R:** Mmhmm.

**P05:** But through this, I approached things in a new way and could have various thoughts.

**R:** Does the theoretical education—using PPTs and such—affect your actual practice? For example, when meditating or managing stress?

**P05:** Not *significantly*...

**R:** Yes.

**P05:** The content wasn't that difficult. It’s better to be told *why* we are doing this before starting the practice, but I felt the theory part was a bit long. I thought it would be better if it were more practice-oriented.

**R:** More practice-oriented, I see. Did you know about mindfulness before?

**P05:** No, I didn't.

**R:** So you applied because you were curious about the title?

**P05:** Yes.

**R:** Was your curiosity about mindfulness satisfied?

**P05:** Yes, I think so. I even felt like I'd want to participate again next time.

**R:** How are you utilizing mindfulness now?

**P05:** First, in stressful situations, I'm forming a habit of looking back at my emotions. And while emotions disappear after a certain time, dwelling on them just triggers more emotions. So I try not to think about the past and focus only on the present sensations. It helped in developing that habit.

**R:** That sounds like it's being used for stress management. How exactly has your perception of and reaction to stress changed?

**P05:** For me, stress starts from recalling past events. I try not to be bound by the past. To forget that, focusing on my current senses is most important. Among the things I learned, "Hand-washing meditation" was good. Feeling the water temperature or the soap sensation helps shift my attention elsewhere, so I don't recall the past events that cause stress.

**R:** If you pay attention to the senses while washing your hands, does the constant thinking about the past actually stop?

**P05:** Yes. It’s hard to prevent the thoughts from popping up, but by moving to wash my hands and focusing on that, I've often experienced the thoughts stopping.

**R:** And what happens then?

**P05:** Then I can forget it and get back to my work without other distracting thoughts.

**R:** So you can refocus on your task. That sounds like you're utilizing hand-washing meditation. How about "mindfulness" in general? How are you using it in daily life, outside of stress?

**P05:** There isn't much use for it yet... I'm not sure.

**R:** I thought you mentioned earlier that you "observe" your emotions?

**P05:** Trying not to think about past emotions and focusing on current sensations—I think that *is* mindfulness for me.

**R:** That’s fine. So you *notice* when you're dwelling on the past.

**P05:** Yes.

**R:** Once you notice it, you think "Let's go wash hands, let's do hand-washing meditation," and you actually move.

**P05:** Yes.

**R:** Before this, did you notice yourself dwelling on the past?

**P05:** Yes, yes.

**R:** Back then, when you knew you were digging into worries, what did you do?

**P05:** I had the will to stop, but the thoughts just kept popping up... it was a cycle of recurring thoughts. Back then, I just tried to do something else. I mostly went for walks.

**R:** Did you do abdominal breathing during those walks too?

**P05:** Not back then. I used to do yoga a lot, so I spent a lot of time on that.

**R:** You mean at a studio, not at home?

**P05:** At a studio.

**R:** The difference now is that while walks or yoga require going somewhere, hand-washing is something you can use frequently in the moment.

**P05:** Yes.

**R:** You're in your clinical practicum now. Did the program impact your clinical experience?

**P05:** Since it’s a hospital, I wash my hands a lot. I try to wash them as long as possible, feeling the sensations in that moment and focusing on myself. Life during clinicals is repetitive and can feel numbing, but I thought of hand-washing as a time to focus on myself.

**R:** You use it frequently then?

**P05:** Yes.

**R:** I guess there are many occasions to wash hands in clinicals? For disinfection and cleanliness.

**P05:** Yes, I use hand sanitizer or wash my hands every time I contact a patient, so it's quite often.

**R:** In those moments, you focus on yourself through meditation. How does that affect you psychologically?

**P05:** It makes me feel "I'm alive" and gives me a more vivid feeling.

**R:** A vivid feeling of being alive. Right. Being able to perceive oneself. In the past, how did you wash your hands? Just... washed them? There's a difference, right?

**P05:** Before, I just washed them out of obligation. My hands get dry if I wash them too much, so I didn't like it. But now, I think of it as a time that helps me, so it feels more valuable.

**R:** How is your heart/mind after washing hands this way?

**P05:** It doesn't hurt... there isn't a specific change in my mind.

**R:** No specific change, but when your thoughts are scattered, washing hands helps stop that and lets you focus on your work. The change in mind might vary depending on the case.

**P05:** Yes.

**R:** Were there any difficult or disappointing aspects of the MMPT program? You mentioned the theory was good but a bit long.

**P05:** I felt I could have focused better if it were in-person during the semester. And... there were quite a few questions that required deep thinking, and it was hard to come up with answers on the spot. Personally, I prefer following physical practices without thinking—like yoga or breathing—so those were no problem. But questions about abstract topics like "happiness," which I don't usually think about, were not easy. For example, "Words that give me courage" or "Words that make me feel confident"—I don't usually have those in mind, so trying to think of them was actually a bit difficult.

**R:** It was hard because you were doing something you don't usually do?

**P05:** Yes.

**R:** You're likely referring to 'Well-being Behaviors' and 'Well-being Cognitions.' It was hard because you hadn't done it much, but how did it feel to actually do those activities despite the difficulty?

**P05:** Sharing with others was fun, and hearing how others think was valuable.

**R:** Did you manage to come up with a few things?

**P05:** Yes, I just said whatever came to mind in that moment, but it might not have been the "best" answer.

**R:** In a sense, just *starting* to think about it is important because many people don't. Since then, have you continued to think about or practice those things?

**P05:** Yes. There were questions about my values, and since I'm writing self-introductions now, those topics come up. I think about them while writing.

**R:** So do you use 'well-being behaviors' or 'words of wisdom/strength' in your daily life?

**P05:** Yes, occasionally. Since it's job-hunting season, if a word comes to mind, I try to write it down.

**R:** In your self-introduction?

**P05:** Just on a calendar. If a word pops up while walking that could be a good material for a self-introduction, I have a habit of writing it down on my phone.

**R:** Do you look back at those?

**P05:** Yes, I have a process of transferring them later.

**R:** Does that mean you use them intentionally? Like, saying those words to yourself when you're struggling?

**P05:** Yes, I focus more on those thoughts.

**R:** Can you give me an example?

**P05:** Just a moment... For example, I wrote down, "Emotions are just passing moments; there’s no need to hold onto them." Another one was, "Let's be a person whose emotions don't become their attitude." I think I’ve been thinking a lot about emotions.

**R:** Those are words you tell yourself to regulate your emotions.

**P05:** Yes.

**R:** And you even write them down. When do you read them?

**P05:** On weekends. I read what I wrote during the week.

**R:** What impact does that have on you?

**P05:** If I hadn't written them down, I would have forgotten them. By looking back, I think about them again. It’s a time to recall how I felt when those thoughts occurred.

**R:** I see. Do those words actually help in specific moments? Is it too abstract?

**P05:** Sometimes I see others and think, "I shouldn't be like that," and I write a sentence. Then my own attitude changes, and it's a time for reflection.

**R:** So it helps with regulation?

**P05:** Yes.

**R:** Good. How about behaviors that make you happy? You said you only did a little during the program, but have you explored and practiced them since?

**P05:** Job hunting is hard, but on weekends, I go for walks, exercise outside, and spend an hour or two with my family every day. That makes me happy. Even when busy, I set aside studying on weekends to focus on happiness.

**R:** It's not easy to find time for yourself in the midst of busyness. But you are doing it. What impact does that have on your life?

**P05:** It makes me feel like I’m not *just* studying; I’m enjoying life enough so I won't regret it later. I think of it as managing stress.

**R:** Even a short time helps with stress management?

**P05:** Yes.

**R:** It sounds like the feeling that "I'm not only studying" is what helps. Is that right?

**P05:** Yes. Exercising builds physical strength and helps organize my thoughts, so there are many benefits.

**R:** So doing things that make you happy actually helps with your studies? (I'm not leading you, just asking.)

**P05:** Yes, that's right.

**R:** We talked about difficulties, but was there anything else you liked about the program?

**P05:** It made me realize the need to take care of my own mind in the middle of a repetitive life. I learned the necessity of focusing on myself and figuring out what I like.

**R:** Did the program help you identify and practice what you like?

**P05:** Yes, just having the time to figure out what I like was helpful.

**R:** And does it lead to practice?

**P05:** Yes, now that I know what I like, I should practice it.

**R:** Earlier, you mentioned that learning about "Compassion" stuck with you. What did that mean to you?

**P05:** It felt a bit like praying. I’m not Christian, and I’ve rarely had the experience of wishing for something for others...

**P05:** Wishing for it doesn't necessarily make it happen, but just thinking about it made my heart feel warm. I think I understood why people pray.

**R:** Just wishing for others makes *your* heart feel warm and better. Have you tried it since then?

**P05:** I haven't done it since.

**R:** We wish for ourselves and for others. I gave a lot of assignments—it's a practice, so I kept telling you to do clinical practice. I wasn't strictly checking and giving feedback, but how were the weekly assignments? Were they too hard?

**P05:** Honestly, I wasn't very diligent with them. But through the checklist—even if not daily, maybe once every three days—I’d think, "What should I try today?" or "I did this yesterday, let's try something else today." That helped me practice. The checklist was more helpful for me.

**R:** It helped you actually practice. In the 5th week, we had Compassion practice as an assignment. Did you do it then?

**P05:** I wrote it down briefly before class.

**R:** You wrote down compassion?

**P05:** I wrote down three things to show compassion for.

**R:** I see. That’s new to me—writing down "content to show compassion for." How did you do it?

**P05:** I just wrote key keywords like "health" and wrote words wishing for others and myself.

**R:** So you wished for them in your heart while writing. That’s one way. We usually visualize the person and say it in our hearts, but you applied your own method by writing. That’s good. But after the program ended, you aren't practicing it much?

**P05:** Yes, life is just clinicals and then assignments at home. Unless it's obligatory, I don't think I'll do it.

**R:** It's hard when you're busy.

**P05:** If there were a book or something where I could see one page a day of what to do, that might work. It's hard to set aside time just to repeat what I learned on my own.

**R:** So you're saying if there were a guide, a reminder, or an assignment from someone else—if there was an external stimulus—you would continue?

**P05:** Yes. Like a simple worksheet or a fill-in-the-blank thing.

**R:** Since it's just a simple thought process. That’s a good point. A system or mechanism to promote practice after the program ends. Thank you for the idea. So, the things you are still using are: "Activity Meditation" (hand-washing), and "Walking Meditation" (breathing), though you did the latter before. And you're intentionally doing things to make yourself happy and writing/reading helpful words. Is that correct?

**P05:** Yes, that's correct.

**R:** Anything else?

**P05:** Not really, other than yoga.

**R:** We consider yoga as meditation. Is the yoga you did before the same as the yoga in our program?

**P05:** The program was just sitting, but at the studio, we use the whole body, so the scope was different.

**R:** Did you focus on sensations during your studio yoga before? Like meditation?

**P05:** Since it's using the body, I think I felt and thought about every corner of my body. Without other thoughts, I focused only on my body and the movements I couldn't do, noticing the changes.

**R:** So you already did it that way.

**P05:** Yes.

**R:** Then meditation in our program probably wasn't difficult for you.

**P05:** Yes, other than thinking about abstract topics, I had no trouble following the practices.

**R:** Right, you seem familiar with meditation, so that part was likely easy. But the programs in the latter half were new and a bit difficult?

**P05:** Yes.

**R:** You said you were curious about mindfulness, and that curiosity was satisfied. How do you understand it now?

**P05:** I understand meditation as a method of mindfulness. I learned the necessity and importance of mindfulness theoretically, which made me think more deeply about stress management.

**R:** Good. We’re almost done. It must be hard to squeeze your brain for answers after a long day... are you okay? Just two questions left. Second to last: Is there anything new you learned about yourself, or has your thinking about yourself changed?

**P05:** I felt more of a need to continue the yoga I’ve been doing since middle school. I always liked stretching, but now I think I should develop it as a stress management method. I want to do it more regularly for both physical and mental health.

**R:** So you used to equate it with physical health, but now there’s a shift to seeing it as related to mental health too?

**P05:** Yes.

**R:** How is it good for your mental health?

**P05:** Clearing my head even for a moment and focusing on my body to get rid of other distractions was good for me.

**R:** You probably did that before too.

**P05:** Before, I just gave meaning to the action itself. If deep breathing was for health, now it’s shifted to doing it to clear my thoughts mentally.

**R:** You're using it more meditatively now that you know it *is* meditation. You've been doing yoga for almost 10 years since middle school?

**P05:** Yes.

**R:** How far can you go with the poses?

**P05:** It’s more about the meaning of steady stretching than technical skills. I don't do very high-difficulty poses.

**R:** You've been doing it to keep your body healthy rather than to do something "cool."

**P05:** Yes, I mostly do basic movements.

**R:** Do you do Hatha?

**P05:** There are many different classes, so I do a variety.

**R:** Like healing, then something more intense... you choose based on the schedule?

**P05:** I try to do various programs like Flying Yoga, Vinyasa, Ashtanga, and Pilates. Doing different things is more interesting and helps me stay consistent.

**R:** The ones you mentioned are all quite intense.

**P05:** True, but the people I do it with are also hobbyists, so I didn't think the difficulty was that high. Though sometimes I get orthostatic hypotension [dizziness] because we change head positions so much.

**R:** Wait, you said earlier you just do basic stretching! Ashtanga and Flying Yoga—you sweat buckets after those! Why did you lie to me? [laughs]

**P05:** I do Healing Yoga too!

**R:** You must be very good at it.

**P05:** My original goal was to build muscle.

**R:** I see. 10 years is truly impressive.

**P05:** It was at school, so it was somewhat obligatory.

**R:** Most kids quit because they develop a resistance to it.

**P05:** I also chose it because I liked it.

**R:** Good. This was an opportunity to realize it's not just for physical health but for mental health and stress management.

**P05:** Yes.

**R:** Last question: Through this program, did you experience any changes in your relationships with yourself, professors, friends, family, or patients?

**P05:** I thought a lot about stress. For example, when I’m working my part-time job, if a customer "throws" their credit card at me... they might have done it by accident, but it feels different depending on how I think about it. If I think it was an accident, it's fine. If I think it was on purpose, it's upsetting. I felt that it changes depending on how I think.

**R:** So how do you think now?

**P05:** I realized that my emotional state and feelings dictate how much I accept the external environment.

**R:** Did you observe that?

**P05:** Yes, I felt it.

**R:** What was the difference? The customer's action is the same—honestly, it's a rude action.

**P05:** When I don't care, it doesn't bother me at all. But sometimes, if it feels repetitive, I get fixated on it and feel like "today is an unlucky day."

**R:** Yes.

**P05:** I think that changed based on my mood. The pool of customers is mostly the same, so if I felt bad that day, I thought maybe there was an issue with my own state.

**R:** Did you observe that in the moment? That "I'm in a bad mood, so I'm thinking worse of that person"? Or did you think of it later?

**P05:** I think I saw myself fixating on those things in the moment.

**R:** So you noticed, "I'm in a bad mood, so I'm interpreting this negatively." What happens when you notice that?

**P05:** I think I become more understanding. I think, "This isn't something I have to feel bad about," and since I've always worked this way, I just think "my level of acceptance is different today."

**R:** How does your mood change then?

**P05:** It gets better. I decide not to focus on it anymore.

**R:** And then do you actually stop focusing on it?

**P05:** Yes.

**R:** That's great. Are you practicing that lately?

**P05:** I'm not working the part-time job now, but when I deal with a sensitive patient, I've thought about how best to treat them.

**R:** Can you share an example?

**P05:** For example, when a patient needs to change positions—instead of just saying "You were lying on your left, now lie on your right," they only change after I explain the specific reason. So from the start, I say, "I need to give you an injection in the hip, so for me to do it comfortably, you need to lie on your right." Even if it's longer, being specific prevents us from feeling bad and allows for better communication.

**R:** You've gained some composure.

**P05:** While observing in clinicals, I think about what would be better.

**R:** You must be so busy and tense just with clinicals, but you're finding composure and expanding your thoughts. We're done with the questions. Anything you'd like to add?

**P05:** Nothing else.

**R:** You have very pretty dimples.

**P05:** Oh, thank you.

**R:** Is that your "charm point"?

**P05:** Yes, I've had them since I was a kid.

**R:** You remind me of the model Han Hye-jin; she has similar dimples. Right?

**P05:** [laughs] Yes.

**R:** They are very charming and make you look even cuter. You should keep them well. We should wrap up. Was it too hard?

**P05:** No, it was fine.

**R:** I can tell you're tired, but you tried so hard to answer seriously. I'm both grateful and a bit sorry for taking your time.

**P05:** Thank you.

**R:** Yes.

**Participant: P06
Date: 2024-04-09
Duration: 55 min 22 sec**
----------------------------------------------------------------------------------------------------------------

**R:** I’ve started the recording, and just in case, I’ll also record this with my voice recorder.

**P06:** Okay.

**R:** Thank you. I’m always anxious about losing this precious data—some people even turn on multiple recorders because of that anxiety. I’ll use two. How have you been?

**P06:** I've been doing well.

**R:** You're in your 4th year now, right?

**P06:** Yes, I’m a senior.

**R:** I see other students are in their clinical practicum. How about you?

**P06:** I finished my clinicals last week, and now I’m back at school for theoretical classes.

**R:** Which one is harder?

**P06:** Both are tough, but I think clinicals were a bit more burdensome than the theory classes.

**R:** Even though theory classes are compressed and the exam schedule is intense, you still feel clinicals are harder?

**P06:** Yes, I think it’s because clinicals are more physically demanding.

**R:** What do you mean by "physically demanding"?

**P06:** Well, I have to leave very early in the morning—at dawn, basically. I work for 9 hours, and then I have to come home and do assignments. It was very exhausting.

**R:** So you have to do assignments *about* the clinical work too.

**P06:** Yes.

**R:** You worked so hard for that month. Good. I sent you a simple list of questions, and you’ve probably looked them over. The first question is: What was your reason or purpose for participating in this program?

**P06:** I was curious about things like meditation, and I figured I’d face way more stress in life later on than I do now. I participated because I thought it would be good to learn how to manage that stress more efficiently.

**R:** Managing stress efficiently... what kind of outcome were you expecting?

**P06:** When I’m stressed, instead of just getting angry or letting it out in a bad way, I wanted to learn how to resolve it quietly through thinking or by focusing more on myself.

**R:** So you wanted to change your reaction, or focus on yourself. What does "focusing on yourself" mean to you?

**P06:** It means not just paying attention to negative stimuli, but—like we did with mindfulness and well-being—paying more attention to things I like and doing those activities one by one.

**R:** I see. Is "anger" usually your main source of stress?

**P06:** Oh, yes.

**R:** Is that right?

**P06:** Yes, lately it has been.

**R:** Before this, how did you respond when you were stressed?

**P06:** Previously, I would either go out drinking with friends or just express my anger directly.

**R:** Drinking or expressing anger could be a way, but why did you want to replace those with something else?

**P06:** First, I felt it was an unhealthy method and that it was hurting me. Rather than just expressing it like that, I thought building up my "threshold" for those stimuli would be better for living my life.

**R:** I see. So drinking or venting anger was actually a disadvantage for you.

**P06:** Yes.

**R:** You mean it's bad for your health when you drink too much?

**P06:** Yes, the over-drinking, the getting angry... all of those things acted negatively on me, so I wanted to change.

**R:** Any other disadvantages?

**P06:** Mentally, it wears me down more, and it can ruin relationships with others.

**R:** Right. With those goals and expectations, you participated for 6 weeks and never missed a single session.

**P06:** Yes.

**R:** That’s not an easy thing to do. And you did the assignments in between. Did the program meet your initial expectations?

**P06:** Yes, it was actually beyond my expectations. I had an idea of what meditation would be since it’s well-known, but "mindfulness" and things like that were completely new to me, so it was impressive.

**R:** What do you remember most? How did mindfulness meditation affect you?

**P06:** I only knew about breathing meditation before, but I learned there are things like body scans. Using the five senses was really fascinating. I learned that just looking at colors with your eyes can also be a form of meditation.

**R:** Did you use that frequently?

**P06:** I tried to, but in daily life, when I’m actually angry, the thought "I should meditate" doesn't pop up immediately. But I made an effort to do it whenever possible.

**R:** How does meditation affect you?

**P06:** To be honest, I don't feel a big impact when I'm already in a calm state. But when my emotions are very high or when I'm angry, if the meditation goes well that day, I feel a real sense of becoming calm.

**R:** You mentioned that getting angry was a struggle. Did you use it then?

**P06:** Yes, I used it a lot right before going into this latest clinical rotation when I was very angry.

**R:** If you're comfortable, could you tell me what happened?

**P06:** It wasn't one specific big event. It was just the day before clinicals started, and thinking about how I’d have to live miserably for the next two weeks made me so irritated. So I meditated with a friend.

**R:** Did that friend already do meditation?

**P06:** No, that friend also participated in this program with me. We are from the same school.

**R:** Which meditation did you do together?

**P06:** We put on a breathing meditation video from YouTube and did it together.

**R:** What change did you feel?

**P06:** We both became calm. We were both very angry at first, but our mindset changed to "What’s the point of being angry?" and we just settled down.

**R:** Becoming "calm" sounds like the anger subsided. Is that right?

**P06:** Yes, comparing the before and after, the anger definitely went down.

**R:** May I ask who that friend was?

**P06:** Park Soo-young.

**R:** I see. It’s interesting that you utilized it together. The second question is: If there were any changes in yourself through the MMPT program, what were they?

**P06:** The biggest change is that I started keeping a diary. I downloaded a diary app on my phone and started organizing my feelings—what was good that day, what was bad. It’s become a habit. I’m still doing it now.

**R:** Recording your good days, your emotions, and your thoughts... it must be hard to write every day. How much do you write?

**P06:** I write almost every day. Even if it's just something short like "I went to school today."

**R:** You’re motivating me! I can't even do that. Were you always good at writing?

**P06:** No. I used to think keeping a diary was meaningless. But while doing the MMPT program, recalling good things made me want to try it. I thought it would be good for record-keeping later, so I’m sticking with it.

**R:** You must keep doing it because it’s beneficial. What do you like about it?

**P06:** I write down everything—the hard times and the good times. When something is hard, writing it down helps me look at the situation more objectively. Instead of just getting angry, I vent it out through writing. It feels like... a "discharge"? That’s why I can be more objective.

**R:** In our program, we warn that diary writing can sometimes make you "sink" into the anger if not done right. But you’re saying you use it to look at yourself objectively.

**P06:** Yes.

**R:** So you’re using it like a "Mindfulness Journal" and a "Well-being Journal."

**P06:** Yes, that’s exactly right.

**R:** And that helps you see yourself objectively. What impact does that have?

**P06:** If I write about something that made me angry and look at it again a few minutes later, I think, "Why did I even get angry at this?" and I truly become peaceful.

**R:** How do you feel?

**P06:** It makes me feel peaceful.

**R:** By looking at the anger objectively, the anger subsides and you find peace.

**P06:** Yes. When I read what I wrote and think about the situation again, I realize there were things that weren't worth getting angry about at all.

**R:** What’s the difference? You were very angry in that moment.

**P06:** Instead of resolving the emotion immediately in an angry way, I immerse myself in the act of writing and organizing. That dissipates the emotion, and then I can see it objectively.

**R:** I’m truly impressed that you’re doing this consistently. By doing this, do you find yourself being objective even *during* a situation, without writing?

**P06:** Pardon?

**R:** Is the writing always a "reflection" after the fact?

**P06:** No, I try to write it right when it happens as much as possible. If I reflect later, I tend to forget everything.

**R:** I see. So you write it right when the emotion surges.

**P06:** Yes.

**R:** If you keep doing that, have you had an experience where you could see yourself objectively even without writing?

**P06:** Yes, but only once. Exactly once.

**R:** That’s okay! Seeing your emotion when it's surging is not easy at all. Could you tell me about that experience?

**P06:** It’s a bit personal, but at school, a professor was being very irritable with us. I got really annoyed too, but then I thought about it from the professor's perspective and thought, "Well, that can happen."

**R:** Did you observe yourself thinking, "I’m annoyed right now, and I’m having these thoughts about the professor"?

**P06:** Yes, I had the thought, "I’m getting irritable just like they are."

**R:** How did it feel to notice that?

**P06:** Once I thought that, the biggest feeling was that getting annoyed like this is completely pointless.

**R:** And you thought, "The professor might have a reason to be irritable." Your perspective shifted.

**P06:** Yes.

**R:** And did your irritation subside?

**P06:** Yes. Once I understood the other person's position, I wasn't really angry anymore.

**R:** I’m sure. Was that really only once? I have a feeling there might have been more.

**P06:** Well, I can't remember any big ones, but there might have been more.

**R:** Good. In a moment of anger, you looked at the anger, noticed your irritability, and realized you didn't need to be angry. You even thought of an alternative—the professor's perspective. Before, you wouldn't have even known you were angry and would have just reacted.

**P06:** Yes.

**R:** And about the diary—you said you write when you're angry. That itself means you already "saw" the anger. You saw it and decided to write. I understand that as you "minding" your anger. What do you think?

**P06:** (Sound cuts out momentarily) I didn't hear that last part.

**R:** I mean, it’s not just the incident with the professor. Usually, you notice "I'm angry now" and go to write.

**P06:** Oh, yes.

**R:** You notice the anger, feel how intense it is, and think "I should write."

**P06:** Right.

**R:** To me, that means you practiced mindfulness on your own state. What do you think?

**P06:** Yes. I didn't start the diary thinking "I must do mindfulness," but as I used it every time I got angry, I realized, "Oh, this is what mindfulness is."

**R:** Exactly. Before, you’d react without knowing you were angry. Now, you know you’re angry and move to an alternative behavior (writing).

**P06:** Yes, that’s right.

**R:** I’m proud. Being able to notice and shift to a different behavior shows you’ve practiced a lot. Any other changes?

**P06:** Other than the diary and trying to do short meditations, there aren't any huge changes.

**R:** What kind of short meditations do you do?

**P06:** When the subway is too noisy or crowded, I get really irritated. In those moments, I try to block out the noise and clear my thoughts. I made an effort to do that.

**R:** So on the crowded subway during your commute...

**P06:** Yes. Before, I used to get unbearably annoyed when I was tired in those situations.

**R:** But now you block your ears and try to eliminate thoughts. How is that possible?

**P06:** Like we learned, focusing on the visual aspect—blocking my ears and focusing on what I see—was effective.

**R:** You focus your attention on what is visible.

**P06:** Yes, just like we did—looking for colors and such. While doing that, time passed, and the anger was "covered up" and passed by.

**R:** You're doing visual sensory meditation. Before, you would have gone home or to work while being annoyed. Now, you arrive with the anger subsided. Is there a difference when you arrive?

**P06:** If I go home angry, my mood stays bad. The biggest difference is that shift in mood. When I'm in a bad mood, I can't do what I need to do, so I think I received a good influence in that regard too.

**R:** I see. That’s how it's being utilized and how it’s affecting you. The third question is one we've touched on, but I'll ask anyway: What changes have occurred in the way you perceive and react to stressful events?

**P06:** Before, I would react immediately to stress. After practicing this, I look back at the situation once more.

**R:** And after looking back?

**P06:** The reaction... disappears? Since the emotion subsides, I feel like I don't have anything to react to emotionally.

**R:** The emotion subsides, so there’s no emotional reaction. You just finished a month of clinicals. Nursing students find that period the hardest. Did the MMPT program have an impact on your clinical practice?

**P06:** This time I was in the operating room (OR), so I didn't have to meet many people. I didn't get stressed from interpersonal stuff. But since I hated the commute, I used what I learned—like meditating with my friend the day before—to reduce my anger.

**R:** Was there less stress this time? As a layperson, I imagine the OR would be very high-tension. Is that not the case?

**P06:** In the OR, we just observe. We don't have tasks to do, so there was no stress.

**R:** Since the OR is a sensitive space, they don't give students much to do.

**P06:** Right.

**R:** So the hard part was just not wanting to commute early in the morning, or the irritation on the crowded subway. How long was the commute?

**P06:** About an hour and a half to the OR. I think it would have been harder if I were in a ward meeting patients instead of the OR.

**R:** Why is that?

**P06:** Meeting patients or more people provides more stimuli, so I would have found it more difficult.

**R:** So you tend to get stressed mostly from interpersonal relationships.

**P06:** Yes, I find it exhausting.

**R:** What makes you angry? What do others do that triggers you?

**P06:** When people demand things I can't do, or when they keep bothering me. That makes me angry.

**R:** Unreasonable demands, or demands you can't fulfill. That’s your "anger point."

**P06:** Yes.

**R:** Of course, that’s infuriating. But you have more clinicals coming up.

**P06:** Yes.

**R:** You'll have to meet patients then. And those situations you mentioned *will* happen. Patients can be very demanding. How do you think you can use the skills from MMPT in those stressful situations?

**P06:** Usually, if a patient or staff member gives me a lot of tasks, I’d be like "Why are they making me do this?" and get very irritated because I'm busy. But now, I think I'll try to take a step back, observe, and just start with what I can do right now.

**R:** What does "taking a step back and observing" mean to you?

**P06:** I’ll try to exclude the emotion of being angry about someone giving me work. I'll just accept the facts and try to understand the other person's perspective. Then the emotional reaction will probably subside.

**R:** So you'll use mindfulness in the way you've been doing.

**P06:** Exactly.

**R:** Looking at yourself objectively and then practicing empathy. That’s great. I hope a "strong" patient appears soon so you can test it out! [laughs] It’s not easy, but we aren't aiming for 100%. Since you've practiced steadily, I expect it will be different. Were there any difficult or disappointing parts of the program?

**P06:** No, not really.

**R:** Does it have to do with the fact that you had meditated before?

**P06:** It’s not so much that, but rather that our generation doesn't know much about meditation, yet I was able to accept it without any resistance.

**R:** Where did you meditate before? On YouTube?

**P06:** Yes, I just watched YouTube.

**R:** For how long?

**P06:** I didn't learn it professionally like in MMPT. I just did 3-4 minute videos for about 3 months before this.

**R:** That’s quite a long time to do it consistently. Is there a difference between those videos and MMPT?

**P06:** On YouTube, it was only breathing meditation. Since it was short, I didn't feel any effect. But with MMPT, I had the theoretical lectures too, so I understood it better. External stimuli can't be changed, so let's change the internal factors—that phrase really stuck with me. I focused on that during practice.

**R:** So the variety of methods and the theory in MMPT helped your practice?

**P06:** Yes, the theory helped a lot.

**R:** If you don't mind, could you elaborate on how it helped?

**P06:** YouTube just tells you *what* to do. "Relax here, focus on your breath there." But MMPT explained it in a way that felt like "letting go" or "releasing," and gave more detail.

**R:** What was that?

**P06:** It helped because you explained it in detail.

**R:** Theoretical details were helpful.

**P06:** Yes.

**R:** Good. I was worried the theory might be boring. This wasn't on the list, but: what was the best part of the MMPT program?

**P06:** Before I started, I thought the researcher would just give instructions and we would follow. But seeing you, the teacher, practicing *with* us made it feel more trustworthy. I liked that part personally.

**R:** What do you mean by "trustworthy"?

**P06:** Usually, a leader just recites things and the followers copy. But since we all practiced together, it felt like a real community.

**R:** I even did the assignments with you!

**P06:** Yes, that sense of doing it together was great.

**R:** I liked it too because it gave me a chance to practice. I felt a bit guilty telling you to do things I wasn't doing myself. You liked the "doing it all together" aspect. Anything else?

**P06:** Nothing comes to mind right now, but if I think of something, I’ll tell you via KakaoTalk.

**R:** Thanks. Next: How are you utilizing what you learned in MMPT now that the program is over? We've talked about a few things—sensory meditation, breathing meditation... any others?

**P06:** I’m not using *everything* perfectly, but I did try to use the yoga/body meditation we learned.

**R:** How about exercising in daily life?

**P06:** I do walking meditation frequently. Just when I’m walking around.

**R:** Frequent walking meditation... you're using quite a lot! And you're still writing the mindfulness and well-being journals. You write about good things, joyful things, and things you're grateful for. What is the benefit of that?

**P06:** First, thinking about good things while writing makes me feel good in itself. It’s also a record I can look back on. If I feel bad later, I can look at what made me happy and try doing those things again.

**R:** What do you expect will happen if you do that?

**P06:** I think I’ll become happier by doing things I like when I’m stressed.

**R:** It helps you actually *do* the things you like.

**P06:** Yes, and it helped me discover *when* I was happy.

**R:** Have you discovered many of those moments?

**P06:** Not tons, but recently...

**R:** Yes?

**P06:** Recently, I discovered that my mood improves when I eat something delicious.

**R:** You discovered that! What else?

**P06:** There’s a game I play, and achieving good results there makes me feel good. And this was surprising, but finishing assignments quickly and getting them out of the way felt really good.

**R:** That’s the sense of achievement. Anything else?

**P06:** Just lying comfortably under the covers in a cozy place.

**R:** You probably discovered even more. You mentioned walking earlier too.

**P06:** Oh, yes.

**R:** So you intend to "give" these happy behaviors to yourself when you're stressed?

**P06:** Yes.

**R:** Have you put that into practice?

**P06:** I’ve tried going for a walk when I’m stressed.

**R:** How about the others?

**P06:** I’ve done many, but I can't recall them right now.

**R:** That's fine. So, by intentionally doing things that make you happy... what change do you feel?

**P06:** I feel a sense of fulfillment from "taking care of myself." And actually doing those things improves my mood, so it’s been effective.

**R:** That can be a way to cope with stress.

**P06:** Yes.

**R:** Next: Did you learn anything new about yourself during or after the program, or did your thoughts about yourself change?

**P06:** I learned that I can feel happiness even in trivial things. And I never really thought about "taking care of myself" before, but now I intentionally think, "I should take care of myself."

**R:** And you're practicing it.

**P06:** Yes.

**R:** How does that make you feel about yourself?

**P06:** I used to think I was a very "simple" person, but I don't think that's true anymore. That’s the biggest thought: "I am not simple."

**R:** What does that mean?

**P06:** As I learn more about what I like and dislike, I feel like I'm learning how to treat myself better.

**R:** So "simple" meant you didn't know yourself well?

**P06:** Exactly.

**R:** But now you know yourself in detail. You’ve realized there are many diverse aspects to you.

**P06:** Yes, but I think I’m still in the process of learning. I still have a lot to discover.

**R:** You said you thought you were a "simple person" before. What is a "simple person"?

**P06:** I didn't think I could change my own mood. I felt my mood only changed based on the external environment, so I felt I was "simple" in a reactive way.

**R:** So you just felt emotions as they came from external stimuli. Reactive. That’s what "simple" meant. How has that changed?

**P06:** Now I try to look at things objectively, a step back. I don't just react immediately; I think more deeply.

**R:** About yourself?

**P06:** I think more rationally than emotionally. I weigh whether I truly like or dislike something.

**R:** You've started observing your state. Not just "That person hit me, so I'm angry," but...

**P06:** Right.

**R:** There’s a stimulus, but you look at your emotions and thoughts objectively, like "I'm angry because of this reason." And you understand your likes and dislikes better, and intentionally do things you like. How does that feel in your heart?

**P06:** I haven't thought about this deeply, but the first thing that comes to mind is that I feel "at ease."

**R:** At ease. That’s wonderful. Final question: Did the program have an impact on your interpersonal relationships? With yourself, professors, friends, family, or patients?

**P06:** I don't just take what others say at face value like I used to. Because of that, my level of reaction to those stimuli has decreased significantly. So, both for myself and the people I deal with, I think we experience more comfort in the relationship.

**R:** So relationships have become more comfortable.

**P06:** Yes, it feels like a burden has been lifted.

**R:** What kind of burden decreased?

**P06:** I don't try to over-interpret what others say. I just accept it as "This person said this." That lifted the burden.

**R:** So you accept things easily. You don't add interpretations. How has that affected real relationships?

**P06:** Everyone is in a sensitive period right now, so we used to fight or bicker a lot. But by just letting things flow and accepting them, those petty arguments have significantly decreased.

**R:** That must be so comfortable. We rarely fight over huge things; it’s usually petty stuff that ruins the mood. You don't meet many "sworn enemies" in a lifetime. [laughs] Looking back at what we've said, is there anything you'd like to add?

**P06:** No, I think I’ve shared all my feelings and what I wanted to say.

**R:** You explained everything so well; I think this will be great data. Are you going home now? Or do you have to study?

**P06:** I’m going home now.

**R:** You must be hungry. You worked so hard. Go home, have a delicious dinner, and since you don't have school tomorrow, I hope you have a peaceful rest.

**P06:** Thank you.

**Participant: P07
Date: 2024-04-09
Duration: 67 min 50 sec**
----------------------------------------------------------------------------------------------------------------

**R:** Thank you. Just in case we lose this valuable data, I’m going to record with this recorder as well. How have you been?

**P07:** I’ve been doing well.

**R:** You look younger somehow.

**P07:** Maybe because I cut my bangs.

**R:** Do people say similar things—like you look younger or more youthful?

**P07:** Not really.

**R:** These days there’s a social climate where people try not to talk about appearance, so I figured you might be being careful. I’m kind of old-fashioned, so even though I tell myself not to comment on looks, I still end up doing it. I should fix that.

**P07:** I catch myself doing it too, suddenly. But yeah.

**R:** You’re a fourth-year student now, right?

**P07:** Yes, I’m a fourth-year.

**R:** Are you currently in clinical practice, or have you finished?

**P07:** This week I’m doing classes, and starting next week I go out to practice.

**R:** Then in March, were you in practicum or theory classes?

**P07:** At my school we alternate every two weeks—two weeks practicum, then two weeks theory—so in March I went out once and then came back.

**R:** So you went out in March, then did theory, and then practicum—now you’re doing theory again, and when that ends you have to go back out again. You must feel like you can’t keep your head on straight.

**P07:** Yes, I’m so busy.

**R:** Since it was during vacation, that’s why you could participate in our program then, right?

**P07:** Right. I think there was a first round and a second round, and if I’d been in the second round I might have had to miss more.

**R:** Exactly. Anyway, you remember really well—like that there were a first and a second round. You must be very meticulous. Out of the seven participants, you’re the first one who actually read through the consent form while filling it out.

**P07:** Really?

**R:** Most people just sign, check the boxes, and leave. You may not have been able to read every detail carefully, but you at least skimmed it before signing—so I could tell you’re thorough. And I guess that’s why you remembered there were two rounds.

**P07:** I think I just remember small things well.

**R:** That’s because you were paying attention—when you focus, small things stick. That’s a good habit.

**P07:** Thank you.

**R:** Of course. I forget the nagging I just did and then nag again.

**P07:** I think that can’t really be helped. I hear it over and over too.

**R:** Yeah, I see.

**R:** I sent you the questions in advance—did you get a chance to skim them?

**P07:** I looked briefly.

**R:** Today?

**P07:** I looked briefly later on.

**R:** That’s totally fine. The point was just to glance at them—who has time to sit and think deeply when you’re that busy? Modern life, busy busy. So, the first question was: What was your reason or purpose for participating in the program? How was it for you?

**P07:** At first, it was just vacation, and I saw the promotion on social media. “Mindfulness” isn’t something you can easily come across, right? So I thought, “Oh, something like this exists,” and since I had time, I joined out of simple curiosity—like, “Should I just try it?”

**R:** Had you heard the word “mindfulness” before?

**P07:** Not really. I just thought, from the phrase “mindfulness,” that it would be some kind of action of taking care of your mind, and I thought maybe it could help me manage my mind on my own.

**R:** So you didn’t already know the term, but the phrase itself gave you an expectation. Nice translation choice, honestly. So it started as curiosity, but the phrase “mindfulness” pulled you in. Then you probably had some expectations while participating—what kind of expectations did you have?

**P07:** I applied and started around the end of January, and at that time I had a lot on my mind and things were hard. I wanted to reduce that sense of burden, and I thought maybe doing this program could help reduce it—that was my expectation.

**R:** So you were feeling psychological burden at the time. If you’re comfortable, could you share what kind of burden it was? If it’s uncomfortable, you don’t have to.

**P07:** It wasn’t anything huge. Since it was vacation, everyone was preparing for employment—studying TOEIC, doing personal things. I’d told myself since third year that I should start TOEIC, but I kind of neglected it—just hanging out and also building a new relationship. Then when I finally tried to do it, it wasn’t going well. I also had to write my personal statement, there were too many things to do, not enough time, and the burden felt really heavy. And on top of that, with that new relationship, it was a period where conflict could happen. So my mind was really complicated then.

**R:** That must have been hard. Fourth year, preparing for employment—you have to study TOEIC and write your statement, and “new relationship” probably means a boyfriend?

**P07:** Yes, right.

**R:** So late January was a time of big burden and stress for you—TOEIC, personal statement, and also conflict with your boyfriend. And you hoped the program would lower that burden and stress. Even though it might be early to ask, how do you think it went—did it meet that expectation?

**P07:** At first, when we started, it was something where everyone talks and shares as we go along, right? But my personality isn’t very outgoing or social, so it felt a bit burdensome. But as everyone did it and we gradually shared, instead of that, I felt like I couldn’t even fully identify what was in my mind, but I felt calmer. It felt like time where I could just rest. In that sense, it was okay.

**R:** So at first, the sharing part—because I end up calling on everyone—felt burdensome.

**P07:** I’m not really someone who’s good at presenting, so that part felt a bit burdensome.

**R:** You have a special skill: you don’t show it at all.

**P07:** Really?

**R:** You sounded very natural and clear—your tone is steady and articulate. So I didn’t realize you were feeling that burden inside. If it had shown, I would have tried to be more considerate.

**P07:** It’s okay.

**R:** I’m glad. If someone feels too burdened, sometimes they run away in the early stage of a study. So I was honestly nervous, calling on people and asking questions. Thank you for sticking with it. You said you hoped to reduce and regulate the burden—how was that part?

**P07:** My feelings from that time are a bit faded now, so I’m not sure precisely. But as time passed—and there was that daily checklist, right?—I kind of forced myself to do it, and while doing it I kept “mind-controlling” myself like, “This is something I have to do anyway, so from where I am now, let’s do only as much as I can.” And I think that helped reduce the burden.

**R:** So after we taught something, we gave homework, and because it was homework, you did it thinking, “I should do it.”

**P07:** Without it, I think I would’ve been too lazy and probably wouldn’t have done much.

**R:** Of course. I did it too, thanks to that. So when you say the burden reduced—what do you mean by that?

**P07:** I guess I accepted those feelings. “The burden reduced”—in a fundamental sense, I think burden really reduces when you accomplish everything. If you focus only on accomplishments, the burden can’t really go down. If you keep thinking, “I have to do that,” and only look forward, the burden keeps growing. But I kept thinking, “Let’s do it step by step, little by little,” and I think that helped reduce it.

**R:** Was that something you intentionally told yourself—like self-talk?

**P07:** Yes. Like you said we wrote it down and posted it. It’s a bit embarrassing, but I wrote things like “Let’s go slowly,” and kept doing that.

**R:** That’s the technique we called “well-being cognition” in the program.

**P07:** Yes.

**R:** Did you use it intentionally after learning it?

**P07:** I used it after learning it.

**P07:** Before, I’d just think briefly, “No, it’s fine, just do it,” and move on. But because it was just a passing thought, the will kind of disappeared quickly. I felt like I wasn’t really keeping the cognition active. So since you said to put it right in front of you, I wrote even small phrases and posted them up there.

**R:** Like on your desk?

**P07:** Yes, I posted them on my desk.

**R:** I do a lot on my computer, so even now I stick things on my computer and look at them. When you do that, what effect does it have?

**P07:** When my personality gets rushed, it’s like nothing else is visible. So everything gets mixed up—“I have to do this, I have to do that.” But to do anything you have to sit at the desk anyway. So each time I sat down and saw what was right in front of me, I’d think, “Okay, calm down,” and it felt like I could settle myself and gather my mind.

**R:** So it has the effect of helping you steady and calm yourself. Great. If there was a change in you through the program, what would it be?

**P07:** I’m not sure if this counts as a change, but I feel like I recognize my emotions better and accept them better, and I think, “I shouldn’t be like this.” For example, I was working a part-time job with other workers, and I got annoyed because their way of working didn’t match mine. I spoke in a kind of snappy, bickering way. After I said it and later looked back, I thought it was too sharp and that I was being really prickly. I thought maybe it’s something I didn’t like, or maybe I was just sensitive, but I felt like, “I shouldn’t be like this.” I think I’ve started having that self-feedback process.

**R:** So in the past, you didn’t notice as clearly, “I’m annoyed right now,” “I’m sharp right now.”

**P07:** It wasn’t as immediate as now. Back then, I think emotions were more prioritized.

**R:** When you say emotions were prioritized—can I interpret that as you became more emotional?

**R:** Then now, if it’s not being emotional, what do you mean?

**P07:** I am emotional, but compared to before, the feedback feels faster. Like, I think, “I’m sensitive,” “I’m angry,” and then I can think, “I should calm this down.” I think it became an opportunity for that.

**R:** So you can observe those things—“I’m angry,” “I’m annoyed,” “I’m sensitive.” When you can observe it, what happens next? It’s a bit abstract, but—how do you feel then?

**P07:** Rather than “how I feel,” it’s more like a bit of guilt. Using the earlier example, I think, “This isn’t something to get that angry about—why am I like this?” and I feel like I should fix it.

**R:** I see. Does your behavior change too?

**P07:** Changing behavior is still a bit difficult. It’s more like I just think, “Let’s do better,” but I don’t think there are obvious behavioral changes yet.

**R:** That’s good. So you notice it and think, “Is this really something to get that angry about?” When you do that, what happens to the anger or irritation?

**P07:** The emotion doesn’t naturally disappear. I recognize it and think, “Let’s put it down,” and that makes me feel more comfortable, but just recognizing it doesn’t immediately change it.

**R:** Of course. You were angry because it felt worth being angry about. In the past, when you didn’t recognize it, you said “guilt,” but it’s also a kind of reflection—thinking in another way. How was that process for you?

**P07:** In the past?

**R:** Yes.

**P07:** In terms of time, I think it lasted longer. It was like, “I’m angry,” that came first—“I have to let this out somewhere.” Rather than calming it down, I felt like I had to express it.

**R:** Then these days, has that “expressing it” decreased?

**P07:** Even if I express it now, it’s not like “bursting out.” It’s more like complaining—rather than clashing with the other person, I try to say, “Why did you do that?” in that kind of way.

**R:** Then your behavior has changed too.

**P07:** I think so. But maybe it’s also just my basic personality—I don’t like fighting or conflict.

**R:** Sure. But still, you’re saying you express it in a more softened way. Any other changes you’ve noticed?

**P07:** This is similar, but when I realize, “This is a situation that makes me tense,” or when I run into a problem and think, “Maybe meditation could help,” then I think, “Should I try it?” And if I have time, I try it—there have been experiences like that.

**R:** First you notice, “I’m tense.” Does that become noticeable to you?

**P07:** The most recent example is that I did interview consulting, and you have to present. When they say, “Introduce yourself,” you’re like, “Hello, I’m ___ with these strengths,” and you talk about your experiences in front of others. It’s so nerve-racking. Everyone’s attention is on me, it feels like my words are being evaluated, and I’m like, “I’m going crazy, what do I do?” And I think, “Is this when you use meditation?” Realistically, I think the only meditation I do is breathing. So I think, “At least I should do that,” and that’s what happens.

**R:** So then you tried breathing techniques or breathing meditation?

**P07:** In that situation I can’t do it for long—just about 30 seconds, 10 seconds. I don’t know if it’s the effect of meditation or placebo, but I was like, “Okay, I can do this,” and I did it.

**R:** How is it for you to notice, “I’m really tense,” and have the option, “I can try meditating here,” or “Meditation could help”? What does having that option mean to you?

**P07:** Over those six sessions, meditation became like a refuge for me. When I feel like I can’t endure the situation, it felt like I could briefly put everything down and heal a little. What was the question again?

**R:** What kinds of thoughts or effects does it bring you to have that refuge—what influence does it have?

**P07:** Having that refuge… I don’t know how to say it—like a safe place. In a burdensome situation, it might be funny to call it my “only hope,” but it’s like… through meditation, I feel like I can get a little better.

**R:** That would feel supportive—like something to lean on.

**P07:** Exactly.

**R:** Now, about stress events—you said earlier that in a fundamental sense, stress truly goes away when the problem is resolved. Still, did you experience any changes in how you perceive stress events or how you respond to them?

**P07:** It’s basically the same context as what I said. I think I spent more time thinking on my own. I try to look at the situation objectively—like, “My position is this,” and if it involves someone else, “Their position is that.” I think I became calmer in how I think.

**R:** So you began to think more objectively—“I’m like this, the other person is like that.” Before that, how do you think you perceived or responded when you were stressed?

**P07:** In the past, if I was stressed, I had a personality where I felt I had to release it all. If someone poked me a little, rather than anger, I’m the type who cries. So I cried a lot. Just because the situation itself felt hard and burdensome—if someone spoke firmly, crying doesn’t solve anything, right? But I just cried and then went back to daily life thinking, “I have to live.” Of course, if there was something I could do to cope, I did, but if it was a situation I couldn’t resolve, I think I was the type to soak in the emotion—like being immersed in “I’m suffering.”

**R:** You felt very overwhelmed. But now you said you look at it more objectively—then what about crying?

**P07:** Now, even if I cry, afterward I think, “What can I do? I have to do it.” It might sound like resignation, but it’s my problem to solve. And if there’s a better way, I think I’ve become more cool-headed with myself. If I did my best within what I can control, I can think, “No, I did my best—within my limits I did what I could, so now let’s let it go.” I think I tried to see the situation—if there was something I could do, I’d do it; if I did what I could, then I’d think, “This is out of my hands now.” So I tried not to let that stress situation spill into my other daily life.

**R:** I see. You can cry because it’s hard, but while crying you also, in a sense, talk yourself through it in a cool-headed way—leading yourself into coping and action. That’s good. It probably got shorter than before, the whole process. You rotate—two weeks and two weeks—right?

**P07:** Practicum?

**R:** Yes, practicum. Practicum is a tough period. Did this program influence your practicum experience in any way?

**P07:** For me, I think there was one thing—compassion, like wishing compassion. Because there are many very sick people, and during practicum there are many heartbreaking moments, and I’m someone who empathizes a lot. I even went to the ICU. So I found myself wishing, “Please don’t be sick, please be happy.” In such a harsh society where everyone is struggling, I’d think, “Why did this suddenly happen?” and I wished people could just be happy—like offering compassion. And I also thought that for the people precious to me—family, me, everyone—of course earning a lot of money is good, but wouldn’t it be best just to be healthy and happy? I started thinking like that.

**R:** Was that something you did intentionally?

**P07:** It wasn’t like, “I should offer compassion.” It was more natural.

**R:** So naturally, from your heart, you found yourself wishing others well—healthy and happy.

**P07:** Yes.

**R:** Does doing that have any effect on you?

**P07:** I think it helped me let go of some desire—like greed. I used to live with the value that making money is very important. But seeing people who came in after accidents on the way to work… it made me wonder if that’s really what matters. The most important thing is being healthy and happy with the people I cherish. Thinking that way, I think I could let go of some of that desire.

**R:** By that you mean desire for money and success.

**P07:** Yes.

**R:** And reducing that—what influence does that have on you?

**P07:** If it goes the wrong way, it can become giving up. So I think there was a condition: within a range where I’m not overdoing it. So rather than being overly greedy and trying to do everything perfectly—cutting sleep and pushing—I shifted to, “Don’t overdo it. Value myself more. Do my best with what I can.”

**R:** So before, were you pursuing those goals while kind of overworking yourself?

**P07:** I’m kind of unusual—like I enjoy extremes. This is weird, but when it’s hard, I make it harder. Like, I don’t need to stay up all night, but I stay up anyway and go take the exam. Or I miss the timing to eat while studying, and I’m like, “Even better.” I had that kind of pattern sometimes. But now, instead of that, it’s like: still do it, but take care of what needs to be taken care of—do it in a healthier way.

**R:** So in the past, not eating or not sleeping felt like something you could even feel proud of.

**P07:** I’m the type who kind of enjoys that.

**R:** But now it’s not “give up.” It’s more like: keep pursuing achievement and success, but also sleep, eat, rest—care for yourself. You’ve got a new equation. “STEM-major tailored,” right? Any other changes in yourself?

**P07:** Nothing else comes to mind right now.

**R:** Great. During practicum, you offered compassion—maybe not in a formal “compassion practice” format, but you wished for patients, and also for people around you. And through that, you also felt more like you should care for yourself. Any difficulties or disappointments while doing the program?

**P07:** It’s a short time to fully absorb everything. So it felt like a “do it briefly and it ends” kind of feeling—like it didn’t become embodied. That was a bit disappointing. But I think it’s something that improves only if I keep doing it consciously, and realistically it’s hard to extend the program duration, so I think it’s an unavoidable part.

**R:** Right—embodiment happens through practice. The methods aren’t that difficult, but as you said, embodiment is the key. Six weeks wasn’t enough to fully embody it. Do you have any ideas that could help embodiment—anything that comes to mind?

**P07:** I think embodiment ultimately depends on the person’s will. I don’t have any breakthrough idea.

**R:** If we say “willpower,” the whole world’s heads would bow in shame. Willpower is important, but it’s not easy. If we assume willpower is hard to control, then what could still help—anything?

**P07:** We did six weeks, one per week, right? It might be hard, but if you contacted us every day, it would keep it in awareness—“I have to do this.” Then maybe I could do it daily. Like I said, at first I think some degree of semi-compulsion is needed. Not like “You must do it,” but like how you used to message, “Let’s do it together today.” For some recipients it might feel burdensome sometimes, but for people with low willpower, I think it could help.

**R:** A reminder could help—others have suggested that too. In our lab, someone is researching by developing an app, actually. And if you look, there are probably programs like that—some companies do it for a monthly fee. I used to wonder who would pay for that, but now I get it.

**P07:** So there was a reason.

**R:** There is a reason. It seems like you’re still practicing after the program too—meditation.

**P07:** I basically don’t do other meditations. Mostly breathing meditation, and sometimes before bed, when it’s quiet, I just notice sensations—only that degree.

**R:** So breathing meditation and sensory meditation. We also practiced many everyday forms of meditation—how about those?

**P07:** And also—walking while noticing natural sounds, smells—that kind of thing. I’m an extreme commuter, so that degree is what I do.

**R:** So you do walking meditation during commuting. That’s impressive. Before MMPT, you probably didn’t do walking as “walking meditation.”

**P07:** No, because these days everyone listens to music while walking, and I like music too, so I listened a lot.

**R:** So now you intentionally do walking meditation rather than listening to music?

**P07:** These days I often listen to TOEIC audio, and today I forgot my earphones, so I thought, “I can’t listen anyway, so I might as well do it.” If I have to study, I study; if not, my commute is long, so I might listen to music for a bit, and when I’m actually walking I do the walking meditation—kind of mixing them.

**R:** You’re weaving it into life—doing it when possible. Nice. Do you notice a difference between doing it and not doing it?

**P07:** When I do walking meditation, I start by intentionally observing the sky and the scenery. So even though it’s the same road I always walk, the world feels brighter—like, “Today is pretty too.” I get this soft, sentimental, warm feeling. If I just listen to music, it’s like “busy modern life,” but if I do meditation while going, I feel like, “Today is a good day.” It’s hard to put into words.

**R:** Right—more spacious, more beauty, more 여유. That makes sense. You’ll be going back to practicum, and later you’ll become a nurse and go through training. The environment is tough—important work, but dealing with sick people is hard. Even dealing with people in a good mood is hard, and sick people are understandably irritable. And the setting is tense—life and death. So, even though you’re doing well now, are there any skills you want to use more intentionally for stress management in practicum, training, or work?

**P07:** Personally, the well-being cognition fits me the best. Seeing something written and recognizing it is much faster for me than when it isn’t there. Without it, I miss things easily. So I think I’ll use that the most.

**R:** I also think you’re already using mindfulness naturally. From what you’ve described, it seems partly embodied—you objectively see your state and tell yourself, “It doesn’t have to be that big,” and so on. And since in nursing settings you wash hands a lot and walk around a lot, you could use those moments too. You said even 30 seconds made you calmer. While washing hands—three minutes—and walking to the ward—one to three minutes—using tiny moments to reset your mind could be helpful.

**P07:** That sounds good.

**R:** Please try it in your next practicum. Great. Now the last question: Have you experienced any changes in your relationships—with yourself, professors, friends, family, or patients?

**P07:** In situations that could have turned into a fight, I think maybe it didn’t go that far—something like that.

**R:** How is that possible—what’s the process?

**P07:** Like I said, I first think through the causes—how did we get to this situation—step by step. If I find what the problem is, then it becomes clear that I just need to solve that. Before, in fights or conflicts, rather than the real issue, emotions get heated and make everything worse—that’s how I see fights. So instead of emotion, I focus on the cause and try to calm down—“That’s not the issue right now. Wait, calm down. I know you’re angry and I’m angry, we all know that, but doing this now won’t help anyone.” I tell myself, “This is the issue—let’s solve this first, and think later.” I think I developed that perspective—seeing the practical problem more clearly.

**R:** So rather than being driven by emotion, you calm emotions and look for practical alternatives—and you even helped the other person calm down.

**P07:** If only I calm down, but the other person explodes, it’s the same—conversation can’t happen.

**R:** That’s probably in your relationship with your boyfriend.

**P07:** It’s the closest relationship, so there are the most fights.

**R:** Right. You said around the start of the program there was more conflict and burden. Did you notice any influence in that relationship too?

**P07:** I don’t know if it influenced him, but I recognize my emotions quickly and try to calm myself down a lot. And because of that, I think I could do mindfulness for myself. So both of us, compared to before, tried to be more careful—rather than the opinion difference itself, we tried to keep it from escalating emotionally.

**R:** So you notice, “My emotions are rising,” “I’m getting worked up.”

**P07:** I really didn’t know before. I really didn’t. But when he says something like, “Don’t get mad,” in a simple way, I can realize, “Oh, I am getting mad,” and that’s a change.

**R:** You must have done the assignments diligently. Mindfulness isn’t easy—people are busy noticing specks in others’ eyes, not their own. But seeing, “I’m in this state,” is hard. Even though you say you’re weak-willed, you practiced consistently. Now I’m thinking maybe next time I should give more assignments.

**P07:** The assignments you gave—

**R:** Yes. I worried people might dislike it—life already has so much homework. But without practice, it doesn’t become embodied. Knowing in your head alone doesn’t matter. So I did them too and showed you. Anyway, you said you reduced your “greed” and increased self-care—that makes me think of well-being behavior. Remember? We also did activities to find behaviors that make you happy and intentionally do them. How about that?

**P07:** If you put it that way, it could be seen that way, but I’ve never thought of it like that. I’ve never recognized it as “well-being behavior.”

**R:** Then if you think now—behaviors that make you happy, comfortable, joyful, fulfilled—things that create a well-being state—have you been finding and practicing those?

**P07:** Like what I just said? Or brand-new activities?

**R:** Either is fine.

**P07:** I think I said this before, but around late February I found a new kind of happiness. I like giving flowers to someone.

**R:** Giving flowers?

**P07:** Yes—choosing flowers, conditioning them, making a bouquet. That’s a small happiness for me.

**R:** Making it and giving it makes you happy?

**P07:** Yes. I think it has meaning only when it’s a gift. I really like seeing someone receive it and feel happy.

**R:** Do you do that intentionally?

**P07:** Even before giving it, I know I feel happy while trimming and arranging the flowers. So when I see flowers, I know I feel better, so sometimes I’m like, “Should I buy flowers?” and I buy them. But these days I don’t, because I don’t have money.

**R:** Even if it’s not expensive, buying something affordable and making it look great through conditioning—that’s kind of genius.

**P07:** It’s kind of special. I think it’s special.

**R:** How do people react?

**P07:** They like it, and they’re grateful, and then that’s it. And I’m like, “Say it properly—face the right direction,” kind of joking. But then I move on. It’s like, I feel good and you feel good.

**R:** It must feel special—most people just buy a premade bouquet, but if you made it yourself, that’s meaningful. Anything else?

**P07:** Other well-being behaviors… This is similar, but walking.

**P07:** When my head gets complicated, I have a routine—I have to go to the Han River or a lake. It’s like a fixed belief: “I have to go there.” When I go, it’s wide open and expansive, so it feels healing, and I feel lighter. I know myself well in that way, so when something is hard, I use that a lot.

**R:** Nice. The Han River is close. What about small streams?

**P07:** A stream?

**R:** Yes—do you need it to be wide?

**P07:** Yes, I need the sky to be visible. Streams are good too, actually.

**R:** So when it’s hard, you intentionally go to open places to release your mind. And compared to before, you also sleep more, eat more—self-care behaviors. Probably more things exist even if they don’t come to mind now. Great. We’re basically at the end—anything you want to add?

**P07:** Do you do programs like this again?

**R:** Again? I don’t have plans right now. Do you want to participate again?

**P07:** No—earlier you said, “Next time I should give more assignments,” and you kept asking in a feedback way, so I wondered if you might do it again.

**R:** I probably won’t run it as a research study again, but if there’s a request, sometimes I do. For example, if Seoul City budget comes and it’s for citizens; or a public health center requests it for residents; or a women’s self-reliance center; or in the past I did it at a university—Kookmin University—for student stress management. More recently, there may be programs for certain groups, like through a trauma center. And what our lab is really trying to do is integrate this into education—public education—middle and high school. We don’t necessarily run full programs for ministry staff, but sometimes we do a one-hour experience session. So I’ll probably keep doing something, but I don’t know the exact target, program, or group.

**P07:** Since you said you’d give more assignments—at first it’s better to do it like now and gradually increase. If there are too many at the start, people might run away.

**R:** That’s true.

**P07:** That’s what I can think of.

**R:** That’s important. Some people feel pressured at first. And nursing students are conscientious, right?

**P07:** Yeah.

**R:** You know what I mean—so you might accept assignments better. But if I were running a group for middle school boys—

**R:** They probably wouldn’t feel burden at all. Right. Great. How was it doing this interview? Have you done interviews like this before?

**P07:** No. This is my first interview. It’s also my first time participating in research like this, so it was a different experience for me too.

**R:** How do you feel after doing it?

**P07:** I hadn’t really thought about it much. Rather than thinking about my changes, it was more like, “If I have time, I’ll do it; if the situation fits, I’ll do it.” But comparing to the past, I realized that doing this program really became a turning point for me to take better care of myself. It felt like I gained an insight.

**R:** I’m glad. If you don’t think about it, it just passes. My advisor used to make people write reflections, which felt rigid, but writing a reflection once does help organize things—like you said. Great. Thank you so much, and I hope you keep practicing so that what you learned helps with stress management.

**P07:** Thank you.

**R:** Great. Goodbye.

**P07:** Goodbye. Thank you for your work.

**Participant: P08
Date: 2024-04-03
Duration: 62 min 21 sec**
----------------------------------------------------------------------------------------------------------------

**R:** With this tool, it records as well, but we won't use the video. Everything will be transcribed from audio anyway. Just in case, I will record with a separate voice recorder at the same time. Yes, yes. For us, interview data is a precious research resource, so I’m doing a double recording in case we lose it. Some people even do triple or quadruple recordings. But I’m not that meticulous, so I just do it twice. If everything is lost even with two recordings, then at that point, I’ll just have to accept that the heavens were determined to spite me. What else could I do?

**P08:** Yes.

**R:** Did you roughly skim through the questionnaire? Yes, I gave it to you with the intention of just letting you have a look, so you don't need to think too deeply. Basically, you participated in the MMPT (Meditation, Mindfulness, Positive Psychology Training) program. We are mainly exploring how that experience was and what kind of influence it has had on your life, P08. Yes, yes. So, the first question was: Is there a reason or purpose why you participated in that program?

**P08:** I don't really watch TV; I mostly watch YouTube. In vlogs like "Miracle Morning," I always saw that those people have one thing in common: they start their day by meditating with their eyes closed or something like that. So, naturally, watching that made me curious about how to meditate, and I happened to see the recruitment for research participants at school and applied. Because I wanted to learn.

**R:** So you applied because you wanted to learn meditation. Why did meditation... I mean, why did it look good to you? What seemed good about it?

**P08:** I also have a desire to live a diligent life, and I’m the type who gets motivated by watching other people's productive days. Seeing those people consistently meditating, I felt like it would be really good if I did it too, so I started.

**R:** I see. In what specific way did you expect it to be good?

**P08:** First of all, becoming calm. It felt good because it seemed like I could start my day very refreshingly.

**R:** Do those YouTubers talk about those kinds of feelings?

**P08:** Yes, I’ve seen that a lot.

**R:** So you had an expectation that it would help you become more composed?

**P08:** Yes.

**R:** The questionnaire only has those few questions, so some people wondered how we could fill an hour. But as I dig and ask questions one by one, an hour passes by before I know it. So it might be a bit painful for you. I might be very nosy. Well then, do you usually feel that you aren't very calm?

**P08:** Yes. I think of myself as quite emotional. Honestly, I shouldn't be like this, but it shows in my behavior based on my mood, and I often can't control my emotions myself. I’ve always found stable people fascinating and I was envious of them. Seeing that those people usually seem to practice meditation and things like that, I participated because I wanted to become a person like that.

**R:** I see. I understand that by "calm," you mean not being emotional. What specific emotions did you mainly want to make calm?

**P08:** I mean, depending on the situation, my emotions... how should I say... they swirl around? Even if I’m in a bad situation, I should handle it flexibly, and my mind should be at peace first, but I’m the type to get completely swept away by it.

**R:** If it’s okay, could you give an example of what kind of situations usually sweep you away?

**P08:** First, when I have a lot to do, I get very anxious. I should solve things one by one, but I just lose the will to do anything and just get so irritated. And also, with interpersonal relationships, even though they change all the time, I tend to give meaning to every single thing or change too easily depending on how the other person behaves.

**R:** You gave two examples. First, feeling anxious when there’s too much to do, which leads to irritation and losing the will to work—getting caught in emotions. Second, in relationships, your emotions rise depending on the other person’s reaction. Did I understand correctly?

**P08:** Yes, that's right.

**R:** How do your emotions rise according to the other person's attitude or behavior?

**P08:** I mean, the other person might be snappy at me if their condition isn't good, or they might suddenly become indifferent or not react. But for me, every single one of those things bothers me so much that I get a lot of stress. Depending on how they treat me, when it was severe, my whole day was terrible just because of one trivial thing.

**R:** How did they act that ruined your day? They probably didn't suddenly slap you in the face... You have a very soft-looking impression, so I don't think you're the type to irritate others. What on earth did the other person do to ruin your day like that?

**P08:** Well, they might have just been in a bad mood, but for example, during clinical practice, a nurse might be busy with work or in a very sensitive situation. Her behavior toward me might come out like that. When I ask something I’m curious about, or when I didn't understand what she instructed and couldn't perform it... those nurses who react a bit... should I say, they get angry? Those who react quite strongly. On those days, my mood was bad all day long. My self-esteem would also be cut down a lot.

**R:** Your self-esteem drops, and your mood... in what way is it bad?

**P08:** Just... first, I’m scared. I’m scared, and I start thinking, "Am I bad at my job?" And since this is a practicum, it's a problem directly linked to evaluation. If I act like this and the nurse seems angry to me, I wonder if it will affect my evaluation, and to put it simply, I'm anxious that my score might be cut... yes, just scared and anxious, I think.

**R:** And that goes on all day.

**P08:** Yes. I keep thinking about it.

**R:** I see. On those days, it must be really miserable working all day. So those two things—feeling anxious when there’s too much work, and feeling anxious, scared, and losing confidence while watching the nurse's mood when someone is unfriendly or snappy... is that the state of emotional fluctuation you’re talking about, P08?

**P08:** Yes.

**R:** Are those the two main emotions you want to manage?

**P08:** Yes. Honestly, the latter is bigger, and as for having too much to do, I’m a senior now, so assignments, clinicals, exams, self-introductions, TOEIC... all the things I have to do have increased so much since winter break.

**R:** I know. When I have a lot of work, I even get alopecia areata. I didn't know, but the hair salon told me there was a hole. So I felt recognized, like "Ah, I was under a lot of stress." Right. So you attended the program with the expectation that you wanted to handle those things calmly. How was it? Did it meet those expectations?

**P08:** Yes, I think it was quite good.

**R:** What kind of influence did it have?

**P08:** I think I’m a person with too many thoughts inside me. So, of course, thoughts lead to emotions, and those become actions, and a vicious cycle keeps repeating. I’ve been worried about how to break this cycle, but it didn't work well. But this time, while practicing the mindfulness and meditation you taught and applying them to real life, the thoughts inside me have significantly... should I say, decreased? I mean, when my thoughts start creeping up again, I tried hard to empty them as much as possible while meditating, and it actually seemed effective.

**R:** First of all, what’s impressive as I listen is that you notice when "thoughts are creeping up."

**P08:** Yes, I...

**R:** Did the program help with that? Or did you already know that thoughts were creeping up?

**P08:** Well, to begin with, I had never really faced stress head-on or pondered *why* this stress was happening to me. I was the type who just took it as it came. But this time, you explained a lot of theories too, right? Through that, I had an opportunity to think about the fundamental reasons, and thinking about it comprehensively, it became an opportunity to realize that, in the end, it’s because I have so many thoughts.

**R:** You realized that it becomes a source of stress for you. Well, it might not be the *fundamental* reason, but you realized it’s a source of stress, and then you started noticing, "Ah, I’m thinking like this now"? Could you give an example? How is it applied?

**P08:** When you explained the theory, we had a session to analyze the situation where I felt stressed, exactly why it happened, and so on.

**R:** Yes, I know what you mean by the stress part.

**P08:** Yes, at that time, I learned, "Ah, this is how you do it," and I think I learned it by naturally applying it to daily life.

**R:** So when we did stress mindfulness, we wrote it down like that. I guided you, and then said "Now try writing it," and you did. Did you master the method then?

**P08:** Yes.

**R:** We did that together. Then, how did you use that when you were alone?

**P08:** Since I have many thoughts, I start thinking about the stressful situation I'm in, and then, just like we did then, I could think deeply about the stress I'm currently receiving by myself.

**R:** "Thinking deeply"... sorry, please bear with me even if it’s painful that I keep asking so nosily. What does it mean to "think deeply"?

**P08:** I mean, the thought "Why am I receiving stress from this right now?" and "What should I do so I won't receive stress like this?" And then ultimately, the thought "I should just not think at all" comes to mind.

**R:** Oh.

**P08:** I also worry a lot about those solutions. And since I get swept away by emotions, I try hard to look at the stressful situation objectively. Like, from a third-party perspective, "Is this a situation worth being this stressed about?" If I think about it that way, I realize that everyone lives like this, and it’s no big deal, but it’s just that I’m in a sensitive state, so it feels bigger. And I mentioned I get stressed because of interpersonal relationships, but I can't adjust others. I can't go to the nurse and say, "I get stressed when you act like this, so please refrain." I can't control others. So ultimately, I reach the conclusion: "This is a problem I don't need to be stressed about, so I should just not think at all."

**R:** When you reach that thought, what do you do then?

**P08:** First of all, a walk. I also mentioned during the program that I walk a lot, but I always did it while listening to music. But when I have many thoughts and am stressed, according to the meditation method you taught, I try to walk without listening to music, looking at the objects around me, and not thinking much. If I'm in a situation where I can't walk, I try to use methods like hand-washing meditation, focusing on the senses—honestly, that part still doesn't work well—but I tried hard to not think as much as possible and distract my attention.

**R:** How is it? What happens when you do that?

**P08:** Well, in the past, I said I would take the stress as a direct hit. Then I would feel so bad that I would sleep a lot. I would just escape the situation itself, and since I had no other way to resolve it, I was the type who just struggled a bit and then forgot as time passed. But once I meditate like that, I can think less, so I think I can escape the stressful situation faster.

**R:** Then do you get the effect of becoming calm as you expected before participating?

**P08:** Yes. I think my own anxiety level is high, but honestly, while it's not completely better now, I think it's gradually getting better as I do it.

**R:** You’re naturally the type with high anxiety.

**P08:** Yes, I am.

**R:** I am too. Even today, while driving here... yesterday, I couldn't sleep much because I was watching the election results.

**P08:** Oh.

**R:** Since I couldn't sleep but had to work, I downed some coffee. It seems like when you can't sleep, you get a bit hyper. There was a faint trembling and a continuous racing of the heart. In that state, because I drank a lot of coffee, the anxiety rose for no reason. Even while driving, I was so scared. Right. It feels like it will be like that forever. Like it'll be like that until I die. There’s this continuous faint tension and trembling, so I came home and meditated too. It doesn't disappear completely, but even so, it's not like it's completely better as you first expected, P08, but you’re saying it is "regulated" and you become "calm." Well then, you might have already answered the second question, but I’ll ask anyway. Through the MMPT program, if there was a change in you, P08, what would it be?

**P08:** The change is that, in the past, I didn't know how to cope in a stressful situation, so I just took it as it came and felt as bad as it got, just waiting for time to pass. But after doing this, I think I learned how to objectively perceive the stressful situation and find a way to escape it quickly.

**R:** What does it mean to "objectively perceive the stressful situation"?

**P08:** I mean, if I look at the situation I'm in from a third-party perspective... for me, what others go through looks like no big deal. Everyone is like that. What I receive always looks huge. But looking from a third-party perspective means thinking about what if someone else went through the exact same thing, and realizing "It's no big deal," "It can happen to anyone." But because it’s *my* business, I’m taking it in a big way and thinking a lot about it. So, while objectification isn't 100%, I try to think that way, and by actually thinking that way, I’ve become quite unbothered. "Unbothered" means becoming calm and composed.

**R:** So you can end it quickly. That seems to be the answer to the third question as well. The third question is: "What changes have there been in the way you perceive and react to stressful events?" Would what you just said be the answer, or could you explain more or give another case?

**P08:** I think that itself is the answer.

**R:** Regarding your clinical practice, you said you used to be troubled all day by a single word. You probably went into practice right after finishing our program. If so, what kind of influence was there in this clinical practice?

**P08:** After that incident, there was a similar situation again. It wasn't as much as then, but I was following a very sensitive teacher and observing. She was a bit annoyed and didn't like it. But since I was assigned to that teacher, I was in a situation where I couldn't *not* follow her. I had to do that for two weeks, and if the shifts overlapped, I always had to follow her. Honestly, in the past, I might not have been able to go. But since this is also a stressful situation, even if I receive that in the hospital, I thought, "Let's only think about this in the hospital, and let's not do it as much as possible outside the hospital, let's not think about it." I controlled my emotions a lot, and when I had a moment on my way home, I thought quietly by myself and meditated, and I think I became okay.

**R:** Thinking quietly like that on the way home sounds like you were practicing mindfulness. Since the hardships of the day came rushing in, you probably practiced mindfulness on that, and then you thought "Right, let's only be tired at the hospital," and meditated to lower your thoughts. What’s different when you get home?

**P08:** Honestly, in the past, I would have just slept immediately. I didn't want to think and didn't want to receive more stress, so I thought the best way was sleep, and I slept right away and just slept through the next day and went back to the hospital. But if I clear my mind on the way home and come home, I think I become productive. I have assignments and a lot of work to do after work, and in the past, I would have postponed and avoided them, but now that my mind is okay, I can complete them until the end and do all my work.

**R:** I see. Because your mood shifted, you could shake it off and focus on your tasks. You seem to be very actively using meditation and mindfulness. Do you apply anything else?

**P08:** For another thing, I mentioned I have a strong tendency to avoid things. In the first semester of senior year, there’s so much to do. I think it’s in the same vein. I said I unconditionally sleep when I’m stressed, so I keep avoiding like that, not studying and just sleeping. But once I meditate, those feelings disappear, so I think I’ve gained the strength to do my work step-by-step.

**R:** Among the skills we learned, are there any others you use? We did mindfulness meditation, well-being behavior, well-being cognition, gratitude, compassion... are you using any of those?

**P08:** Gratitude... three things a day. But I saw other people doing that a lot too. So, as I’ve been stressed lately, I’ve become sensitive and snappy toward my family and friends. So I tried to write a gratitude journal, but it didn't work well. I always forget and just sleep. But I’m trying to think with a positive mind as much as possible. If someone did something good for me, I’m trying hard to think about that a lot.

**R:** So you don't *write*, but... some people are really good at writing. But those people are usually those who were already good at reading and writing. Personally, I don't like reading and writing much either.

**P08:** Yes.

**R:** You intended to keep writing, but that didn't really happen, and you’re practicing intentionally thinking about good things or things you’re grateful for. Could you tell me a little more about that?

**P08:** Once I have a negative thought, I’m the type to get swept away by it. In the past, I think I really had those thoughts a lot. Just... it’s a bit cringy, but "Why is this world like this?" and I would even dislike the people around me. If I have those thoughts, I only see the bad points. But now, there are many people who are obviously good to me, and my friends are always cheering me on and being good to me, so "Why do I always think like this?" So today, if my friend says something encouraging to me, I try hard to recall that one more time. When I have bad thoughts, feel anxious, or get annoyed with people, I just recall the words of encouragement and support from my acquaintances, my mom, dad, or my grandparents. Thinking "There are such good people, let's not think too negatively and try harder."

**R:** Is there a particular word you recall?

**P08:** Recently, I had a very anxious period regarding job hunting. There were rumors that they wouldn't hire new recruits because of the doctor's strike, or that it would be pushed to the second half. "Will it work even if I do this? There are no positions to begin with." Then, students from top universities would start getting pushed back in specs, and I was very anxious that I might not be able to enter a hospital that used to be easy to get into. I was quite depressed, but my dad told me, "You don't have to get a job, just graduate. You don't need to get a job, Mom and Dad are here, so you don't need to get a job, why are you struggling alone? You don't have to go to a good hospital, you can rest if it's hard, it's okay to even take a leave of absence if job hunting is really hard." Those words were very helpful.

**P08:** It relieved my burden. No one ever told me to go to a good hospital, so why was I making it hard for myself with my own thoughts? So I realized I was tightening the noose around myself, and I let go of a lot and became more comfortable after hearing those words.

**R:** Your dad said such reassuring words.

**R:** In our time, there was something like "If it's hard, come down to the countryside and just farm." In our time, most parents were of the age where they farmed. For people like us who came up to Seoul to study, they would say "Hey, if it doesn't work out, come and farm together." Like a place I can finally go to, "Right, if it doesn't work, I can just go and farm." It sounds like that. In our era.

**P08:** Yes, that's right.

**R:** So when you’re anxious, do you intentionally recall your dad's words?

**P08:** Yes, that's the one I recall the most. In the end, no one is pestering me to get into a good hospital, and no one wants that, but it's just that my own expectations for myself were high, so I was making it hard for myself. I always think that while doing my work, and when I don't want to do all the work I have, I just think "I don't have to do it, let's just graduate," and then...

**R:** So when you're feeling pressured and anxious, you intentionally tell yourself "You don't have to do it, just graduate"?

**P08:** Yes, the words I recall the most are those.

**R:** How is your mind when you recall those words?

**P08:** At first, when I heard them, should I say I felt empty? I felt that emptiness.

**P08:** But thinking about it, the goal I had when entering school was the license anyway, so why did I care so much about grades and things like that? I felt a sense of liberation hearing those words.

**R:** So when the pressure and anxiety rise while studying, you intentionally think "You don't have to do it, graduation is enough." In a way, after telling yourself that, you’re saying you feel a sense of liberation?

**P08:** Yes, that's right.

**R:** When you feel liberation, then what? Do you play more because you feel liberated? Or...

**P08:** I said if I have too much to do, I end up doing nothing at all. But once that pressure on myself disappears, it's not like I completely throw it away, but because of my personality, I *can't* completely throw it away, so rather than not doing it at all and avoiding it, I’ve gained the strength to do it one by one. So I do those things well, and I mentioned that during my last practice, I meditated on my way home and managed my mood. With things like that, my study efficiency has actually gone up compared to before. More so.

**R:** Rather, because you told yourself "Just graduate," you felt liberation, and as a result, you became calm and are actually performing more. That’s what you mean. You said that in the beginning. You said you didn't do it because you felt pressure and anxiety, which led to irritation. That’s good. You use those words intentionally these days?

**P08:** Yes, I’m using them even more these days.

**R:** And you also intentionally think more about the people around you who are good to me, or people who cheer me on, or good things like that. Instead of just receiving it like in the past, if you intentionally think that way, what kind of influence does it have on you, P08?

**P08:** Wait a moment.

**R:** Yes.

**P08:** Sorry, could you say the question one more time?

**R:** I mean, you’re intentionally thinking about the good things that happen to you. Whether other people are being considerate, or sending you... is it emoticons? Anyway, sending those things, saying good words, and so on. In the past, you just received them, but if you intentionally think about them, what influence does it have on you?

**P08:** Then I can start my day refreshingly, and my mood just gets better, and whatever I think about, it doesn't become negative. I’m receiving so much support around me, and no one is saying anything to me, so I don't need to do this well, let's just gain strength and finish it. "Let's cheer up." I think it always ends with that conclusion.

**R:** Yes, good. As a result, you don't go toward negative thoughts like you used to, but rather your mood gets better. These things are good. Next question: Were there any difficulties or regrets while doing our program?

**P08:** Difficulties, regrets... I didn't really feel any. I didn't really feel anything to be supplemented further.

**R:** I see. Then were there any good points?

**P08:** The good points were... learning how to escape from stressful situations or being taught meditation. It's hard to learn those things, and though there might be many on YouTube dealing with those themes, it's rare to have an opportunity to actually try it out like a practicum. It was very helpful that you did it with us. And also, we shared our feelings and things like that with the other participants, right? I felt like we were doing it together, and it was a chance to hear a lot of other people's thoughts, so it was very informative.

**R:** You’re a good listener. You have a special talent for listening. Some people focus when the instructor speaks but don't listen well when other participants share their feelings. So my advising professor always adds a bit of "medicine" when sharing reflections. She increases motivation by saying "There is much to learn from other people's stories too."

**P08:** Ah, yes.

**R:** Right. If you listen to their stories well, there are things that help you too, so listen carefully. She always adds those words. I didn't dare to add such words because I was always watching your reactions, worried you might feel pressured. But since you, P08, listened on your own and took it as your own learning, you have a very receptive and listening talent.

**P08:** Yes.

**R:** That will be a very helpful skill later when you work as a nurse. You agree. Was there an example of that?

**P08:** Before going into clinical practice, we learn the virtues of a nurse in basic courses, but it didn't really hit home. But I particularly felt it during the psychiatric clinical practice. This isn't a job that simply requires skills; you really need the ability to empathize with people, you need to listen to the patient well, and you really need a heart for others to work in this job for a long time. I felt that so much, so I think that’s a really important quality.

**R:** Of course, of course. I’m giving too much of a personal story, but someone I interviewed for counseling got breast cancer in her 30s. Breast cancer in her 30s... she had surgery. But after surgery, you wake up in the recovery room, right? But from the moment she woke up in the recovery room, she found herself listening so well to the nurse. She didn't even say she was in pain even if it hurt; she endured as much as possible. She even helped the nurse next to her. Later, while doing counseling, we looked into why she tried so hard to look good to the nurse and why she tried to endure the pain and help her... she wanted to live so much.

**P08:** That's true.

**R:** She was so scared from the moment she entered the operating room. Since it’s breast cancer in her 30s, it’s possible. And later, it's very unpleasant when you wake up. She said she was very cold. Hearing that made me realize you could be such a existence.

**R:** Like a lifeline. She was constantly watching the nurse's mood to look good, enduring the pain to help... Yes, good. I’ve given a personal story. I think the rest of the questions are done. I just need to ask two more and we can finish. After our program, or during the program, is there anything you newly learned about yourself, P08, or any change in your thoughts about yourself?

**P08:** I think I have many thoughts. In the past, I might not have realized it because I didn't think about that side at all. I was in a state where I didn't even realize I was thinking. But through this class and meditation, I realized "I’m a person with a very noisy mind inside." And I thought I was very good at enduring, but surprisingly, I was receiving a lot of stress. I didn't know it either, but I wasn't managing it, and I realized it was a very necessary situation. Through this program.

**R:** What changed once you realized that?

**P08:** First of all, the fact that I try to escape from that situation quickly is the biggest change. Without avoiding.

**R:** Escaping quickly is different from avoiding?

**P08:** Yes. If I avoid, I just take the situation as it is and avoid it through sleep or not doing what I need to do. But now, I completely stop the thoughts, control my emotions, and try to cope with the situation as much as possible. That’s how I’ve changed.

**R:** I see. You didn't know you were receiving a lot of stress, but you realized "I’m receiving a lot of stress, I’m struggling, I needed care." How was your heart once you knew that?

**P08:** Just... I’m glad I found out even now. Because this is school life. If I receive this much stress even in school life, how much more would every day be a stressful situation in a workplace? If I hadn't realized this quickly and there was no correction, it would have been really hard. That’s why these days everyone leaves after three months. I thought I would have been one of them. So I’m glad to know even now, and I think "Let's build the strength to handle it flexibly by practicing a lot before getting a job."

**R:** Has it influenced your interpersonal relationships as well? Whether it’s the relationship with yourself, professors, friends, family, or patients... is there an influence on those relationships?

**P08:** What I felt the most... I mean, what hit home the most is that I feel like I’ve become very calm. Compared to before. First of all, since my mood is stable and okay... I mentioned my mood shows in my behavior. Since my heart is okay, of course I treat my friends well. And although I shouldn't, the ones it shows to the most are my parents or my younger sibling—my family. But these days, I hardly get annoyed with my family. These days, I almost never do, so my dad actually said, "Something must be going well these days." So I’ve changed a lot in that sense. The relationships have gotten better, and there aren't many fights within the family anymore.

**R:** I see. You have a very gentle-looking impression, but it seems you were different with your family.

**P08:** I shouldn't be, but they are the most comfortable people, and that sense of reassurance... "Anyway, Mom and Dad will keep liking me no matter what I do, and so will my younger sibling." So although I shouldn't, I think I was the most... how should I say... I got annoyed the most with my family.

**R:** Right. You stretch your legs after looking at where you’re going to lie down. Your family became the place for you to lie down.

**P08:** Yes, that's right.

**R:** You were being mean, but you probably could breathe in the middle of your hardships. That’s good. Having such a family is a truly grateful thing, so you should think about that again today. I think you probably felt it as you were talking. Talking like this, you probably naturally felt "What a grateful existence my family is to me, how lucky I am." I felt it together with you. Could I ask just one more?

**P08:** Yes, yes.

**R:** we do a lot of theoretical classes, right? Motivational state theory, information processing capacity, intentionality, constructivism, and so on. We put in a lot of such theoretical education. How is that?

**P08:** But I didn't really feel there were many theoretical classes, and rather... I think you have to learn something first for it to come out as behavior and be practiced. So the theoretical classes were very helpful to me. And not only that, I remember we meditated together every day. So it felt like 50-50 to me, half and half, so it was okay. In this way.

**R:** How did the theory help you perform?

**P08:** First of all, if you just tell me "Do this" blindly, I don't even know why I'm doing this behavior, there's no reason and no grounds, so I myself don't feel the necessity to do it, so I don't do it and it doesn't hit home. But learning the reasons for doing mindfulness through theoretical classes, I could establish the rationality and the reason for the behavior, like "This is truly an important thing, this is why I should meditate."

**R:** That’s true. If I just say "It's good, so just do it," you won't do it. But "This is the principle, I should try it." That increases motivation. I'm supposed to give a one-hour special lecture for Ministry of Education employees the week after next.

**P08:** Ah, yes.

**R:** After hearing you, P08, I should boldly include theoretical education. Since it's an hour, I was thinking "Should I take it out? Should I just let them experience it?" But after hearing you, I realized you shouldn't just tell smart people to do it. They need to be convinced. So I gained the tip that I shouldn't be intimidated and should use theory boldly and then have them practice. Good. You’ve explained it in such detail that I think I’ve obtained rich data.

**R:** How do you feel? Have you done such interviews before? Research interviews like this.

**P08:** No, this is my first time.

**R:** I see. How was it?

**P08:** I said I only think and don't write down, so I didn't have a chance to think "How did this program come to me?" but while thinking and talking about it, I think I organized my thoughts a lot, thinking "Right, that's how it was" myself.

**R:** That’s good. I’m very happy to hear that. Since you’ve decided to take care of yourself, we also found many well-being behaviors for ourselves, right? Even though it was a short time. So that seems to be the walk.

**P08:** That's right.

**R:** You intentionally go for walks now.

**P08:** Yes, when it really, really doesn't work, I try to refresh by breathing fresh air and walking. So when I'm in a bad mood, I intentionally go for a walk.

**R:** I understand that you’re using well-being behavior like that. Besides that, if you combine and do things little by little... nursing work is very easy to get exhausted in. It's an occupation vulnerable to burnout. So meditation is good, mindfulness is good, everything is good, but I think well-being behavior is really good too. Behaviors that can give you positive emotions—whether it's meeting friends and playing, or walking, or exercising, or drinking when the time comes... anyway, I hope you do those things from time to time even in the middle of hardships. For the long term. I feel that way. Yes, yes. Good.

(Omitted: Discussion regarding the 30,000 KRW incentive and administrative delay at the university.)

**R:** Yes, yes. Anyway, thank you so much for today. I’ll be in touch.

**P08:** Yes, thank you. Goodbye.

**Participant: P09
Date: 2024-04-10
Duration: 68 min 7 sec**
----------------------------------------------------------------------------------------------------------------

**R:** Yes, thank you. So, I’ll record with this device simultaneously as well. Yes. Did you have a chance to skim through the questionnaire?

**P09:** Yes. Well, I couldn't write down the answers at the bottom, but I looked through them once to see what kind of questions there were.

**R:** You did well. It was meant for you to just glance through, rather than thinking too deeply. I think you’ve got a general sense of what I’ll be asking. Overall, the main focus is: what was your experience with the MMPT (Mindfulness, Mindfulness-based Positive Training) program like, and how has it influenced your life? That’s what this interview is about. Especially, I’m curious if it was helpful during your clinical practice. Since you, P09, happened to have suffered through 12 weeks of practice right after the program ended, I’m expecting some very vivid stories. Yes, yes. So, the first question is: Is there a reason or purpose why you participated in this program?

**P09:** First of all, when the notice came out, the word "mindfulness" really caught my eye. At that time, I was doing an internship. I was doing an internship at a hospital, and I think I felt for myself that I needed some "mindfulness" (care for the mind). So, I thought I wanted to do this program. But at first, it was supposed to be in-person, and the time overlapped with my schedule, so I couldn't apply. Then, another notice came up later saying it had changed to non-face-to-face (online) and told those who wanted to re-apply to check the announcement. That notice said we would learn about mindfulness and positive psychology. Those two parts—mindfulness and positive psychology—really caught my eye, so I applied.

**R:** So, it happened that you were doing an internship at a hospital. I guess that’s a bit different from regular clinical practice.

**P09:** Yes. I did a 4-week internship and then 8 weeks of school clinical practice, so it was 12 weeks total. This hospital internship was something I applied for personally.

**R:** You must have been having a hard time back then.

**P09:** Well, the internship I did this time was directly linked to hiring. So, whether I realized it or not, I think I felt quite a bit of pressure. Also, I had to be at the hospital all day starting in the morning, and since I was in a position where I was being evaluated all day, I felt like I needed to take better care of myself mentally. Also, after finishing the clinical practice during my junior year, I felt like I needed time to reflect on myself. That's why I became more interested in this program.

**R:** What kind of expectation does the word "mindfulness" trigger for you?

**P09:** I thought, "Wouldn't I be able to care for myself more?" Like self-care.

**R:** You had that expectation. And you mentioned that after your junior year, you needed time to reflect on yourself. What did you mean by that?

**P09:** I did my first clinical practice in my junior year. I used to think that I should focus on taking care of patients and that the patient must always come first. But as I kept doing the practice, I found myself getting exhausted. Going through that process, I realized that I need to be filled up and take care of myself first before I can provide better nursing care and do my best at the clinical site.

**R:** So, what you expected from mindfulness was time to take care of yourself. You said you got exhausted while trying your best for patients. In what way did you experience that exhaustion?

**P09:** First of all, because I was tired at the clinical site, I found myself unable to go to the patient one more time to talk to them, and I felt like my energy was lacking.

**R:** That must have been the period transitioning from junior to senior year, where you start job hunting and then actually head out into the field. So, you were attracted to the words "mindfulness" and "positive psychology" and joined, but there was no time. The in-person sessions were 8 times, 2 hours each. Because of that, there was only one participant. So, reflecting the reality, we changed it to online, reduced the time to 1.5 hours, and reduced the sessions to 6. We completely revised it. And we promoted it to students from other schools, so students from four different schools joined. We didn't mention the school names there, right? But the participants came from four different schools. That in itself might be the reality of nursing students—it’s not an easy reality to participate in an 8-session, 2-hour in-person program.

**R:** I think I experienced that indirectly. Anyway, so you participated. When you joined, what did you hope would change by the end?

**P09:** Do you mean what I expected at the beginning? Or how I actually changed after it ended?

**R:** I mean, when you first joined, what was your goal or expectation?

**P09:** I expected to become better at stress management. By reflecting on myself, I wanted to recognize what stresses I had and manage them well so that I could take care of myself. I didn't want my struggles to be expressed outwardly, so I hoped to have more strength and energy when going to clinical sites. That's what I expected.

**R:** I see. So is that connected to "care"?

**P09:** Yes. I mentioned "self-care" is about looking back at oneself first. So, I expected to be able to focus more on my own voice and look back at my own stress and myself first.

**R:** How is it? Did it meet those expectations?

**P09:** First of all, yes. Seeing that the things the MMPT teacher said come to mind when I’m having a hard time, I think it was effective.

**R:** I see.

**P09:** The lessons pass through my head one by one.

**R:** For example, what kind of things?

**P09:** For example, I realized through this program that I am the type of person who tends to ruminate on thoughts, especially at night. I realized, "Ah, I’m a person who ruminates a lot." Before, I used to keep thinking, "Don't think like this, don't think like this," and struggled with it. But now, instead of fighting the thoughts, I recall the lesson that I should just bring up another thought and let the original thought flow away naturally. That comes to mind. So, I’m getting a lot of help from that.

**R:** Then what do you do? How does that actually help you?

**P09:** When I keep regretting or worrying about a certain event, I try to think of other things.

**R:** What kind of thoughts?

**P09:** Instead of just focusing on that one thought, I realized that if I think of something else, the original thought naturally disappears. So, I think about the good things that happened today, or things I did well... just thoughts that I consider "good," even if they aren't perfectly "positive." Just things that were good today. Thinking about those things helps me calm down and fall asleep naturally.

**R:** So, while you were thinking about regrettable or bad things, you intentionally choose to think "well-being thoughts."

**P09:** Yes, I think so.

**R:** Then do you fall asleep naturally?

**P09:** Usually, I have those thoughts while lying down at night. Then I can't sleep if I’m captured by those thoughts. But as I think [well-being thoughts], my mind calms down, those [bad] thoughts disappear, and I’m able to sleep well.

**R:** You’re utilizing it that way. Originally, we asked you to write a "Well-being Journal." You didn't write it, but you recall well-being thoughts instead. By recalling well-being thoughts, the negative thoughts naturally go down, and you feel comfortable and fall asleep. That’s a great way to use it. There’s the method of writing, but also the method of quietly recalling well-being events of the day in your head. What kind of things have you recalled?

**P09:** I don't remember the exact details because I’ve done it so many times. For example, something simple like "What I ate today was delicious," or "It was fun spending time with my family." On Wednesday, I had those [negative] thoughts again at night, but as I looked back and thought about what the professor said during the group conference, I felt relieved.

**R:** Then what happens?

**P09:** Then the negative thoughts I had before disappear.

**R:** How does your heart feel then? How is your mood?

**P09:** My mood becomes comfortable.

**R:** I bet. Since you're comfortable, you can fall asleep naturally. You’ve used it that way. That’s great. Before, did you not know that you were a person with many thoughts or that you ruminated negatively?

**P09:** I don't think I really thought about it that way.

**R:** Then how did you come to realize it?

**P09:** Well, during the lecture, you said there are people who ruminate like that, and those people have high stress and anxiety. While listening to that, I thought, "That sounds like me." After that, when I observed myself, I saw that I was indeed doing that. I have a lot of thoughts, and those thoughts were increasing my stress. That's how I found out.

**R:** You started observing yourself.

**P09:** Yes.

**R:** So, you noticed, "Ah, I’m doing that now." In other words, while observing, you noticed you were ruminating on negative things. And doing that must have made you feel bad. Just like we discussed in the group session, anxiety, depression, or anger must have risen. So, you did "positive rumination" instead. Is that awareness—or mindfulness—happening frequently these days? Before, you said you didn't notice it. How much do you notice it now?

**P09:** Once I realized that I’m a person who ruminates a lot and has many thoughts, things changed a lot. Before, I would just be immersed in those thoughts. But now, I can think, "I’m a person who has a lot of these thoughts," and I can step back and look at the event more objectively. So, it really helped in reducing negative thoughts. I’m still trying to do that, and I am doing it.

**R:** I see. Once you started seeing it, it became easier to see it from then on.

**P09:** Yes.

**R:** Then, this might be a similar story, but you mentioned earlier that you expected to recognize when you’re stressed and cope with it. How is that going?

**P09:** About whether I can cope with stress?

**R:** Yes, you said you expected to notice your stress and want to cope with it well. How is that?

**P09:** The reason I get stressed is because I think too much. But I’ve learned a "skill," so to speak, to escape from those thoughts. So, I received a lot of help.

**R:** Do you notice that you are receiving stress? P09, do you notice now, "Ah, I am stressed right now"?

**P09:** Yes.

**R:** Is there an example?

**P09:** That I am receiving stress...

**R:** Even if it’s not a specific example, is there a situation recently where you noticed you were stressed?

**P09:** There was something that happened for the first time during my clinical practice. About 2 or 3 weeks after this mindfulness program ended, I expressed my emotions for the first time during practice. Usually, I’m the type to just say "It's okay, it's okay" and try to let things go. I never really expressed my emotions. But this time, I felt "I’m really struggling in this situation," and I talked about those feelings to the other person. Others might think, "Is that such a big deal?" but I had never done that before—telling someone how I feel. So, I was surprised at myself, experiencing that "I can actually talk about my emotions."

**R:** You seem a bit proud of yourself.

**P09:** It was like the first step of learning to speak my emotions—getting to know my own feelings. Right after that happened, the professor during clinical practice mentioned something about "talking about emotions." So, I thought, "Ah, I’m in this first stage right now."

**R:** I guess you used to express positive emotions a lot. But the emotion you expressed for the first time was probably a "not-so-good" emotion that you hadn't expressed before—like feeling bad or hurt. You noticed that and expressed it directly to the other person. How did you feel after noticing and expressing it?

**P09:** At first, I felt guilty. I wondered, "Is it okay to say this?" But actually, after saying it, the relationship with that person got better. So, I felt, "It’s okay to express it."

**R:** Was that person a friend?

**P09:** Yes, a peer. A peer I went to the same department with for clinical practice.

**R:** But after expressing it, the relationship actually got better.

**P09:** I said things like, "When you said that, it was a bit hard for me. I felt a bit bad at that part." It turns out the friend didn't have that intention at all. They were actually thinking of me and saying it, but I had misunderstood. So, when that friend said, "I said that because I was worried you might find it hard," I understood their perspective. We went to eat something delicious and cleared it up, and we became closer.

**R:** You did so well.

**P09:** Yes.

**R:** You expressed it so well, too. That’s an "I-Message," right? Do you know I-Messages?

**P09:** Yes, "I..."

**R:** The "I-Message" technique.

**P09:** Yes, delivering "me."

**R:** Did you learn the I-Message technique? Or is that just your personality?

**P09:** Well, we learned about I-Messages during psychiatric nursing, but it wasn't like "I must use this." It's just that my personality is such that I can't say negative things to others, so I think I said it very carefully, which might have been good.

**R:** But you really delivered it using the I-Message format—stating the facts and then your feelings. If you state a judgment, it becomes uncomfortable. But because you delivered it well as an I-Message, the other person didn't feel bad but rather felt sorry and could explain their position. Your first attempt was great. Good. Besides that experience, is there any other change in how you perceive and react to stressful events?

**P09:** During clinical practice, there are many moments when my heart feels uncomfortable. For example, if I have to follow a certain nurse, and a new nurse has just joined, the preceptor nurse might scold the new nurse. Standing next to them, I feel uncomfortable and stressed, as if I’m being scolded too. But in those moments, if I go to wash my hands, it helps me switch my mood. Focusing on the senses helps. So, what I used most during practice was washing my hands and using that time to empty my thoughts. Washing hands is the most natural action in that setting, right?

**P09:** It makes me look like I’m following hygiene rules well. So, by focusing on the sensation of washing my hands, I can switch out of that uncomfortable situation. Then I can do it again.

**R:** What do you mean by "doing it again"?

**P09:** I mean going back to where those people are, sitting next to them, and listening again. I feel refreshed and gain the strength to follow them and observe the practice again.

**R:** That worked out well. Nurses wash their hands so often. I was about to say... please continue.

**P09:** I wash my hands very often too. But I never used to focus on the hands while washing. I would just keep thinking [about stress] while washing. So, it was fascinating. Thinking, "My hands are precious," feeling the sensation of the water and the sensation of the hands... thinking like that really made the [bad] thoughts go away and made me feel better. My mood changed.

**R:** It works just as we discussed.

**P09:** Yes, yes. It was fascinating.

**R:** So you used that a lot during practice. Washing away bacteria and washing away the mind—double effect. Anything else come to mind?

**P09:** I think I used those the most: Well-being Cognition, Sensory Meditation... yes, those.

**R:** What is "Well-being Cognition" to you?

**P09:** Like I said before, knowing that doing a certain thing makes me feel good and using that.

**R:** I see. That’s good. Among meditations, you mainly used hand-washing meditation. Did you use any other meditations?

**P09:** The breathing method. Breathing meditation. I did that a lot during MMPT, and I’ve done it a few times since it ended.

**R:** How did you use breathing meditation?

**P09:** Outside, I can't really close my eyes, so I just focused on inhaling and exhaling.

**R:** Was that just in your daily life? Or for a specific event? Hand-washing was clearly a coping mechanism for a stressful situation. Was breathing something you did in your spare time?

**P09:** Well, what comes to mind is that I had an interview after my internship. For the final interview. Before the interview, I did the breathing method. And I found myself teaching it to the person next to me, another interviewee. I told them, "Focus only on inhaling and exhaling, then the anxiety and tension can disappear." So we inhaled and exhaled together while waiting for the interview.

**R:** You used it that way. And usually, did you practice it in your spare time as a "training," like the homework we gave during MMPT?

**P09:** Yes, I tried to do breathing in moments when I felt tense.

**R:** Do you still continue to do it now?

**P09:** It's not like I set a time and say, "I must do breathing meditation today," but when it comes to mind, I do it.

**R:** Right, breathing is something we always do anyway. I carry a stopwatch around because I’m always ready to teach it. Even during counseling, if someone is struggling, I’ll start it and teach them. You can do it while waiting. But you, P09, just do it when you think of it, quietly feeling the breath without a set time. That’s good. Very good.

**R:** Anything else? Any other changes in how you perceive or react to stress?

**P09:** Overall, what was best for me, as I said, was learning to put another thought on top when many thoughts are rising. I learned that the most. And it’s been a huge help in my life. Just learning that one thing made the program worth it. I think about that a lot.

**R:** That’s a great method. Usually, people do meditation when they can't sleep. Meditation leads to relaxation. Body scans are actually meant to keep the mind awake while the body rests, but what can you do? Not being able to sleep is so painful. So many people use body scans for sleep. But hearing you today, P09, I realized that ruminating on "Well-being Cognitions" is another way to fall asleep. I’m getting ideas from you. In counseling, so many people have sleep issues these days.

**R:** I might suggest that to them. But I think there’s a reason for it. (I shouldn't be explaining this during an interview, but...) I think you, P09, are already well-trained in seeing the positive.

**R:** The fact that it works so well for you... of course, negative rumination comes up naturally, but I feel that you’ve already had a lot of practice in your life seeing the positive side. What do you think?

**P09:** My personality is a bit like that. I try to think simply. (This might sound contradictory to what I said before.) While I have a lot of negative thoughts, I also try to think simply and positively. My personality is a lot like my mother's. She’s very positive. She always writes "positive" in the personality section of any form.

**R:** I see. So you already had some "self-training" or influence from your parents. That's why those things could come to mind quickly. When you notice, "Ah, I’m ruminating negatively," you can quickly switch to "I’ll think about the good memories from today, there were plenty of good things." The fact that the transition happens quickly shows you’re already trained. Many people can't do that.

**R:** Some people say, "There’s absolutely nothing good."

**P09:** I think my faith also has an influence. I’ve been a Christian since birth. In Christianity, they always emphasize loving and things like that. So I think that plays a part.

**R:** Yes, that's also a form of training. But this time, a balance seems to have been struck. Seeing the positive is necessary and an asset. But we also have a negative side.

**P09:** That’s true.

**R:** We have positive emotions, but also negative ones. Hearing you today, it seems you’ve started to see, acknowledge, and express the negative side too. How was that?

**P09:** It’s true. It’s fascinating to me too. I was someone who couldn't express negative things well. Even if things were negative, I wouldn't respond and just let them pass. But being able to manage and express those negative thoughts... it’s really fascinating.

**R:** When you say "negative," do you mean negative emotions?

**P09:** Ah, yes.

**R:** For example, what kind of emotions?

**P09:** Anger, fatigue, irritation, struggle, depression, sadness.

**R:** Those are natural aspects of being human. How did you deal with those emotions before?

**P09:** I think I just ignored them when they came up.

**P09:** I thought it was "wrong" of me to have such thoughts. I thought I should smile more at the other person. Even if I was angry, I tried to be kind to the person who was angry at me. It might have helped in that specific moment by not starting a fight, but it didn't help the relationship. I would slowly close my heart without expressing anything, drift away, stop contacting them, and naturally close the door. But by expressing it, I can resolve my negative thoughts about that person, see them in a new light, and clear up misunderstandings.

**R:** Do you notice now when anger, fatigue, irritation, struggle, depression, or sadness arise in your heart?

**P09:** Yes.

**R:** When you notice them, what do you do now?

**P09:** I think, "I’m struggling because of this right now." If it’s caused by someone’s words or actions, I try to talk to them about it and express my feelings. If it’s caused by my own mistake, I try to think, "What was the positive part of this event?" and try to see it as an opportunity for growth.

**R:** You’re practicing mindfulness.

**P09:** Is this mindfulness?

**R:** Yes. It’s fine even if you don't know the name. We learned about meditation, mindfulness, and positive psychology, right? Meditation is about the senses—that’s well-defined. But in interviews, I find people are practicing mindfulness without knowing it. Listening to you, you’ve internalized mindfulness skills in just 6 weeks, which is impressive. Mindfulness is about observing your own state. "I have this heart, this emotion, these thoughts." You said you notice when you have many thoughts or negative rumination—that is mindfulness.

**R:** Mindfulness allows you to see yourself objectively. That’s why people say, "The situation looks objective." You are seeing yourself objectively.

**P09:** Yes.

**R:** So you notice, "I feel bad because of these words, I’m doing this." You are noticing these things, which means you are practicing mindfulness. And through that, you choose your actions.

**P09:** Yes.

**R:** Before, you acted out of habit—acting kind when angry, acting okay when hurt. You were trying to follow Jesus' example, but perhaps by force.

**P09:** Yes. It’s not wrong to say that I was trying to act like a saint when I’m not one. I’ve gained a lot of "self-objectification." It’s really fascinating to me too.

**R:** You might have felt a bit embarrassed saying that. How do you feel?

**P09:** But truly, looking back at myself... seeing myself objectively is hard to do without learning it. Without someone telling you, you might be able to look at your emotions, but...

**R:** Yes.

**P09:** ...it's not easy to stand one step behind and see "I was like this, I have this negative side, I’m thinking these thoughts." Discovering and accepting that "This is also me" makes me feel like I’m growing. My life has become more comfortable and "free" lately.

**R:** The word "free" really resonates with me. It sounds like you’re saying "It’s okay to be like this"—okay to be hurt, okay to be angry. "Free" is a word that touches me too. (I shouldn't talk too much, but...) I’m not a Christian; I’m a Buddhist.

**P09:** Ah, I see.

**R:** But a close friend, a pastor’s wife, had a daughter getting married. I went to the church for the wedding. The senior pastor gave a sermon about the love of God and Jesus. He said that the love of Jesus is not something humans can [perfectly] do; we are just "mimicking" it. We are trying to follow it. I don't know the exact details, but I was deeply moved. To me, as a Buddhist, that love of Jesus didn't seem different from the love of Buddha. It’s pure and flawless. We just "follow" it.

**R:** Like a song, we keep trying and following. We get angry, sad, and frustrated, but we follow anyway. That was my understanding, and I felt a shiver at someone else's wedding. I don't know if I’ve conveyed my intention well to you, P09.

**P09:** Yes, it was conveyed.

**R:** Good. Then, you might have already mentioned this, but after the program, is there anything new you learned about yourself or any change in your thoughts about yourself?

**P09:** First, the new discovery is: "I am a person who gets angry too. I am a person whose mood can turn bad." Before, I really thought of myself as a "good child."

**P09:** But I realized there are many different versions of me inside. I still remember that picture [from the program]—the characters for Sadness, Joy, and so on. I thought I only had "Joy" inside me. I thought I had to be like that. But I found that I also have "Sadness" and "Anger." Discovering that allowed me to express myself in more diverse ways, and it’s okay. My way of life has become more colorful and fun. I’m finding new sides of myself: "Ah, I like doing this." I’m trying to find more things I like.

**R:** You’re trying to find things you like. Have you found any?

**P09:** Yes. First, I found that I like exercising. So I started playing badminton with my sister and brother. And I thought I didn't care much about eating, but it turns out I like it—eating delicious food.

**P09:** Also, I wasn't the type to go out and buy things for myself. For example, I wouldn't just go to a cafe to buy a drink. But now, as a "gift" to myself, I buy a drink. During the clinical practice lunch break, I think, "Let's eat something delicious today," and I go buy it.

**P09:** Yes, just with that, my life has become so much more diverse. Day by day.

**R:** You said life has become "colorful" and "fun." I can see why. You’re giving yourself many fun things.

**P09:** Yes, now I keep looking for what’s fun and noticing "I feel good when I do this," and I try to do those things.

**R:** That is also mindfulness—noticing "I’m like this right now," "I’m happy right now," and savoring the delicious food. If you do those "Well-being Behaviors" for yourself, how does it relate to coping with stress?

**P09:** First of all, doing that makes me feel like a "precious person." I’m someone who deserves this kind of treatment, a precious person. As much as I treat others, I treat myself more. Before, I wouldn't even buy things I liked for myself. But now I do. And because of that...

**R:** Because of that?

**P09:** When I receive stress, my self-esteem has gone up. (I’m not sure of the exact word.) Because I take care of myself first, when I’m stressed, I can just do those [well-being] behaviors. So the time I spend stuck in those [bad] thoughts has become shorter.

**R:** You mean when you're stressed, you think, "I can just eat something delicious later" or "I’m going to play badminton later," and that helps you get out of the stress faster. Your heart has become "wealthy."

**P09:** Yes, truly. Even if I get stressed, like you said, if I think about going to play badminton later...

**P09:** ...the [bad] thoughts disappear and I feel excited and happy. Then I can do the studying I need to do. Before, I would get exhausted from those thoughts and spend my time doing nothing. But now, I think, "I’ll do this [well-being thing] later," and that gives me the strength to do things in the meantime. Like, "I’ll go eat something delicious later," so I can study one more page of English now.

**R:** You’re someone who responds well to "carrots" (rewards)!

**P09:** I didn't know that about myself. I thought I didn't need carrots. But I’m that kind of person.

**R:** "I’ll give you one more candy, try this." Then you do it happily. It must have been hard for you to endure until now.

**P09:** I wonder how I’ve lived my life so far. It’s fascinating.

**R:** Everyone is like that. You’re practicing the "Well-being Behaviors" we learned.

**P09:** Yes.

**R:** The name doesn't matter. Hearing that it’s melted into your daily life makes me want to dance.

**P09:** I want to dance even more!

**R:** Great. This will be the last question. Regarding relationships—professors, friends, family, patients... (or a boyfriend, if you have one)... have you experienced any changes in your relationships?

**P09:** Well, I don't have a boyfriend, so... no. But regarding the relationship with myself, I can look at myself first. I’m doing self-care, and I feel I’ve become more precious. And as my actions have become more natural...

**P09:** In relationships, I think if it’s only one-sided, it can't last long. If I feel like I’m sacrificing, it becomes...

**P09:** ...harder, and I end up having negative thoughts about the person. But by taking care of myself first and listening to my own feelings, my attitude toward others becomes "genuine" rather than "fake." I treat them more "freely."

**P09:** I use the word "free" a lot, but it’s like treating people without any hesitation.

**P09:** So, for professors, I used to be unable to ask questions. I felt I had to look "proper and honest" in front of them—that I had to be that kind of student. But during the group conference, I thought, "I’m curious about this. Should I try asking?" and I asked a lot of questions. Solving those questions made me happy, and I could focus more on what the professor was saying. I became more interested in the subject and liked it more. And...

**P09:** This is really fascinating—since I started taking care of myself first, and because I know what I like, when I think of a friend, I wonder "What would this friend like?" I become interested in what they like. And I want to...

**P09:** ...share the good things. For example, if I like a certain tea, I think about gifting it to a friend. Before, I used to give to the friend before I even ate myself, and I would buy for the friend before buying for myself. But that was very exhausting to sustain. Now, I try it first and think, "This is delicious, I should go eat this with my friend next time."

**P09:** So by caring for myself first, I’ve actually become able to think more about others.

**R:** You mean you care for them "sincerely."

**P09:** Yes, yes.

**R:** You treat them well. That’s good. Even without trying so hard, that heart comes up naturally. The word "free" resonates with me too. I see. Thank you for investing more of your time.

**P09:** Actually, this feels like a time for me to look back at my own heart.

**R:** What do you mean by that?

**P09:** I wondered, "Am I really practicing mindfulness?" Before the interview, I was worried, "What if I’m not doing it the way I was taught?" But while talking, you said "That is mindfulness," and that it’s "seeing yourself objectively." I’m not good at explaining things and I forget words, but as you defined it for me...

**P09:** ...I realized, "Ah, so that’s what it was. What I did was a part of mindfulness." Thinking, "I’m practicing mindfulness right now, I’m looking back at my heart well," this has been a time to define and organize things.

**R:** That’s great. Actually, while trying to make others understand, you often come to understand it yourself.

**R:** That’s why I listen so intently. I want to understand you and help you understand. In that process, the speaker often gains understanding too. Hearing you today, P09, I truly felt that you are practicing the "self-care" you first hoped for. I deeply resonated with that. And I’m amazed and happy that such changes happened in such a short time. I felt, "It is possible." Those are my thoughts.

**R:** Anyway, if you wait a bit, it will be processed. Thank you so much for today. You explained everything in such detail that I thought several times, "This data is so good, I must use this." Thank you so much.

**P09:** I just spoke at length... it’s fascinating that you thought of it that way.

**R:** Anyway, you must be tired. I hope you have a comfortable and happy Friday evening. I’ll be in touch.

**P09:** All right, thank you.

**R:** Goodbye.

**P09:** Thank you.

**Participant: P10
Date: 2024-04-26
Duration: 60 min**
----------------------------------------------------------------------------------------------------------------

**R:** Yes, yes. We’re set up. Have you had a chance to look through the questionnaire?

**P10:** Yes, but I didn't look at it super closely; I just kind of skimmed through it.

**R:** That’s fine. That’s enough. I gave it to you with the intention that you’d just take a glance. If you’ve seen it to that extent, you probably have a general outline in your head. By the way, you’re very beautiful.

**P10:** Me? Oh, no.

**R:** I always saw you as a tiny face [on the screen] during the program, so I only had a vague impression. But seeing you in a large photo during this interview, I think, "Youth is truly a force to be reckoned with." You’re a senior now, right?

**P10:** I’m a junior.

**R:** You were a junior?

**R:** Oh, I see. The program was originally for juniors and seniors, so you joined right as you reached that level?

**P10:** Yes.

**R:** I see. Okay. So, was this your first time doing clinical practice?

**P10:** Yes, that’s right.

**R:** It must have been very tough for your first practice.

**P10:** Yes, it was.

**R:** How much practice did you do?

**P10:** We usually do three 2-week sessions per semester, and I’m currently on my third and final one of the first semester.

**R:** So, is it like: 2 weeks of practice, a little break, then 2 weeks again, another break, and so on?

**P10:** Yes. We do 2 weeks of practice, and during the remaining 2 weeks—you know how other majors have regular theory classes—we have to cram all 4 weeks' worth of missed theory classes into those 2 weeks. Then we go back out for 2 weeks of practice, then 2 weeks of theory, then midterms... right now, it’s a practice week.

**R:** So you’re on your third practice now. You must be exhausted lately.

**P10:** Yes, but I’ve gotten a bit used to it now.

**R:** Since you say it’s your third time, it shows how humans can adapt to survive. [Laughs] Good. As you saw in the questionnaire, the main question is this: How was your experience participating in the MMPT (Mindfulness-based Positive Training) program, and what impact did it have on your life? That’s the core question, and I’ve broken it down into several sub-questions. The first one is: Was there a specific reason or purpose for you to participate in this program?

**P10:** Yes. Psychologically, I tend to have big "highs and lows," so to speak. My mood swings are quite large, and I feel anxiety very easily. I had never tried meditation before, so I thought if I had that experience, it would be mentally helpful. Since things were likely to get even tougher in the future, I applied thinking it would be a good experience. Through mindfulness meditation, I learned various ways to relax, and I was able to find the method that fits me best. By turning that into a habit, I think it brought a lot of stability to my life. In a positive way.

**R:** You summarized that so clearly, as if you had pre-organized your thoughts.

**P10:** But I feel like making it a consistent habit is still a bit difficult.

**R:** Yes, that’s true. That part is a new beginning in itself. But you said you joined because you thought things would get tougher—specifically because of emotional fluctuations and anxiety. I understand that clearly. But could you explain more about what you meant by "it will get tougher"?

**P10:** Well, I have more clinical practice coming up, and starting from junior year, I have to prepare for employment. I felt like I would be placed in many mentally anxious situations. That’s what I meant.

**R:** So, you meant that having to do both practice and job hunting would be harder. What kind of help did you expect to get for those things?

**P10:** I participated in the program hoping to become someone who can cope stoically even when anxiety finds me, and someone who can face these situations with more stability.

**R:** I see. So the main thing was your emotional fluctuations, which seem to be centered around anxiety. Not so much anger or depression going up and down, but rather anxiety rising and falling—and you wanted to regulate that.

**P10:** Yes. And I feel a lot of stress over trivial things. Even if I make a small mistake, I worry, "What if everything goes wrong because of me?" I have a personality that worries a lot about small things. The anxiety coming from that... especially when I have to give a presentation, I get so nervous that my voice cracks like a goat. My friends seem so stoic and good at it, so I used to ask them, "What do you do when you’re nervous before a presentation?" I thought meditation could be one of the methods for that, so I joined.

**R:** Have you actually tried utilizing it?

**P10:** Yes. But since habit formation is hard, while the program was ongoing, I did it with a sense of "semi-forced" obligation, keeping the record charts and all. After that, as I went out for practice and got busy, I became a bit neglectful, I must admit. But even now, I’m still consistently doing "Action Meditation" (Mindful Activity), which suited me well back then.

**R:** What kind of action meditation are you doing?

**P10:** I live alone (off-campus), so when I wake up early in the morning, the sun isn't up yet. At that time, I open the window, look outside, and meditate. After a while, the sun starts to rise, so I watch the sunrise too. Also, since I usually eat very quickly because I’m in a rush, I’m trying to meditate in relation to eating as well.

**R:** How does doing that "Eating Meditation" influence you?

**P10:** Doing those things gives me time to reflect on myself and think about myself. Rather than having "random thoughts" (distractions), I focus on the action itself, so those distracting thoughts seem to fade away.

**R:** You mean distracting thoughts are reduced? Then, when those thoughts are reduced, how does that affect your psychological state, P10?

**P10:** When I have distracting thoughts—for example, a mistake I made during practice—I should just leave it there. But I would bring it home and think, "I messed up again today," "I’m the type of person who won't succeed," or "I'm going to make a mistake again tomorrow." I’d keep thinking like that. But now, by doing action meditation or eating meditation, distracting thoughts are reduced, and I spend less time thinking like that. So I feel like I can separate my work from my life.

**R:** You said your worry and anxiety were your concerns, so this must have an impact on those as well.

**P10:** Yes, exactly. Those worries and anxieties ultimately come from my own thoughts. Since those distracting thoughts decreased, the time spent worrying and being anxious decreased.

**R:** So the "amount" of worry and anxiety decreased. You mentioned that you feel particularly nervous and worried during presentations, and compared to others, you feel you tremble and worry more. You had high expectations that meditation could be a coping method for this. Have you had any experience using it in that context?

**P10:** I did try it once, but it was just some deep breathing. Before a presentation, there isn't much time to do anything else. I did some deep breathing, but maybe because the anxiety was too big or I was busy memorizing my presentation script, it wasn't perfectly effective.

**R:** What kind of effect were you expecting that it failed to reach?

**P10:** I hoped to be less restless and anxious, and to be able to present skillfully and stoically. Maybe because it was my first time, it wasn't as stoic as I had imagined. But I find meaning in the fact that I at least made the attempt.

**R:** Do you intend to try it again?

**P10:** Yes. Later, when I’m very nervous before a job interview, I plan to try it then.

**R:** How do you plan to try it then?

**P10:** At that time, since I’ll have to keep preparing for the interview, I’ll probably try to control my mind through deep breathing. Also, since "Sensory Meditation" (Sensory Awareness) didn't suit me well, I think I’ll go with deep breathing, Yoga Meditation, or just meditating while looking at the scenery.

**R:** You mean while waiting for the interview. I really hope you try it. But as you know, it doesn't work well if you only try to do it suddenly.

**P10:** Exactly.

**R:** So, as you said, if you make it a habit, it might show its true effect when you’re really desperate. Besides action meditation, what else are you utilizing?

**P10:** Action meditation and Yoga meditation suited me well, so I’m doing those two together consistently.

**R:** You’re doing action meditation and Yoga meditation consistently. Besides meditation, we did other things too. Is there anything else you’re utilizing?

**P10:** We received training on "observing oneself from a third-party perspective." I used to do that even before the program, so I’m still doing it consistently. I’ve always had time for self-reflection, so that’s become a well-established habit.

**R:** Could you give an example of how you do that?

**P10:** Like when I’ve fought with a friend, or if I had a tiff with my mom. Say I was supposed to hang out with her, but we fought over something trivial and I came home alone. First, I try to think about it. "Was I too emotional?" Then, looking at it from the outside, "How could I have handled it better?" Since the time I can spend with my mom is limited, I really want those emotional situations to happen less often. So, looking as a third party, I see "I was a bit emotional then." Next time, I shouldn't just be emotional just because I’m comfortable with her; I should think before I speak. That kind of thing.

**R:** It sounds like you’re doing self-reflection. Is there another example?

**P10:** Hmm, another one... when I was out for practice. It wasn't about blood pressure, but IVs. When the nurse was giving an IV, the patient kept moving, so I was holding them. When starting the IV fluid, I should have held the arm while turning it on, but I didn't think that far ahead. I just turned on the fluid, the patient moved, and the needle came out. Eventually, for elderly patients whose veins are hard to find, we finally found one, but the nurse had to come back and redo it. And I had made another mistake the day before as well. So I went home thinking, "Why am I always making mistakes? I’ll probably mess up tomorrow too. Did I hurt the patient?" I kept worrying all the way home. But in that situation, I tried to "remind" (reflect) again. "If I were that nurse, I would have liked the student to do it this way."

**P10:** And "I didn't think about this part." I think, "I will never make this mistake again in the future," and I also try to comfort my worried heart. "It’s my first time, it can happen." I try to do some "mind control." Because if I keep worrying, I can't do anything.

**R:** I see. So it sounds like you observed your own state as a third party, noticing, "I am worrying continuously right now." You saw your state, then tried to look at the situation more objectively, and then switched to an action of "patting yourself on the back" (self-comfort) to cope. Is my understanding correct?

**P10:** Yes, that’s right. Although even if I pat myself on the back, I still worry quite a bit at home... but I try to shake it off this way. Because I just keep thinking about it otherwise.

**R:** But you mean you *notice* that you are thinking about it continuously, right? You really are seeing yourself from a third-party perspective. "Ah, I’m doing that right now." Is there anything else you’re utilizing? It sounds like you’re using self-comfort techniques... is that right? You can say no if it isn't.

**P10:** I think we shared some "quotes" or encouraging phrases back then. Right, during this midterm period, I almost gave up on one subject. The day before the exam, while studying, I thought, "Should I just quit?" But everyone else is probably in the same situation, so I shouldn't give up until the end. So I wrote, "Hang in there just a little more, there’s only one day left," on a sticky note and put it in front of me. Looking at that gave me strength while I studied for this midterm.

**R:** You put it up and looked at it while studying.

**P10:** Yes. Telling myself "I can do it" is good, but having a written phrase in front of me was better because it’s always visible. Since there’s no one around me to tell me "It’s okay, you can do it," it was very helpful to see that quote I put up for myself.

**R:** What kind of feeling does that stir up in you mentally?

**P10:** Even when I want to just drop everything, I think, "I should keep going, there’s only one day left." It gives me the strength to hold on until the end. I feel less anxious, and it feels like someone is cheering for me.

**R:** Was this your first time utilizing that?

**P10:** I’ve been using it occasionally for a while.

**R:** You’ve been writing them down and using them each time.

**P10:** I like famous quotes and phrases, so I look them up a lot. So I wrote those down.

**R:** I mostly use the computer for work, so I stick them on my computer. I change them out occasionally. But you, Seung-yeon, where and how do you use them?

**P10:** I usually write "Today's Quote" at the top of my diary or study planner. For this exam, it was so urgent—just one day before—that I used a "memo pad" (not a sticky note, but a plain one). I just placed it in front of my desk stand. Usually, I write them in my diary or planner.

**R:** Yes.

**P10:** Or you know those pretty postcards they sell? I write on those with a felt-tip pen and keep them.

**R:** That’s nice. Writing beautifully with a felt-tip pen—that could be a good idea for me too. Is there anything else you’re utilizing? You seem to be using a variety of things, which is very interesting to me.

**P10:** Anything else...

**P10:** I don't think there’s anything else.

**R:** Well, feel free to tell me if you think of something else. Then, through this program, if there was a change in yourself, what would it be?

**P10:** I felt that it stabilized my psychological state, which used to be very anxious. I felt more stabilized during meditation. It’s not a perfect habit yet, so when I’m in a huge rush, I still draw a blank. I’ll have to try making it more of a habit to know for sure, but I do feel a sense of stability in anxious situations.

**R:** You experienced that kind of change. Any other changes?

**P10:** I feel like I’ve developed a daily routine. For example, every morning I open the window, breathe in the air, and watch the sunrise. I do that literally every single day. I wouldn't have done that before, but I started it when I began living alone, so it’s completely become a habit. It’s for ventilation and meditation at the same time. It feels like a daily routine. As soon as I wake up: open the window, meditate, take the ice I prepared, make a cup of coffee, and refill the ice tray. That’s my fixed daily routine.

**R:** I can imagine it vividly, and it sounds quite cool. Listening to you, I can picture a young woman starting her day like a scene from a movie. But honestly, that’s not easy. It’s so easy to just rush through the morning. I got a very "relaxed" vibe from your description. You must be maintaining it because there’s something good about it, right? What is so good about it that allows you to keep it up?

**P10:** Doing that makes me feel like I’m taking better care of myself. When the air hits me, it’s always refreshing, which is nice. And since it’s become such a habit...

**P10:** It feels like I can "refresh" myself. Like I’m starting the day anew.

**R:** Before, you wouldn't have started that way. You probably started your day frantically and mindlessly like everyone else, right?

**P10:** Back then, I just woke up and, without any time to think, just packed quickly and thought, "I gotta go fast." And I’d forget things. But now, I check if I’ve packed everything, and I feel more relaxed. "Relaxed" is the biggest feeling. Having that bit of extra time in the morning rather than being so busy.

**R:** I see. What’s the benefit of starting the morning so relaxedly?

**P10:** By starting the morning relaxedly...

**P10:** I think I have fewer distracting thoughts. These days, when I "space out," it feels like I’m truly spacing out rather than having distracting thoughts. I used to have so many random thoughts when spacing out, but now I can just be still. Doing action meditation and observing nature makes me feel like my mind is being emptied, which makes me feel even more relaxed.

**R:** So by starting the morning that way, you begin your day in a stable psychological state. You keep doing it because you find it enjoyable. Some people might find it boring, though.

**P10:** But for me, it’s a routine, so I think, "Now I’ll go do the ice," and start the day happily. It doesn't feel boring at all.

**R:** Yes, I really resonate with that. I thought it would be boring to start the morning that way, but it’s surprisingly not. Right. Usually, we feel we have to turn something on—watch something, listen to something—but it’s not boring even if we don't. Strange, but true. All right. Regarding stress... life is basically a series of stresses, right? Have you experienced any change in how you perceive and react to stressful events?

**P10:** My immediate reaction when facing a stressful situation hasn't really changed. But my perspective when looking back *after* the situation is over has changed a bit. I look back as if from a third-party perspective. "It happened. I didn't need to be *that* stressed back then." I look back like that. So there is that change. But *while* receiving stress, I’m still too immersed in it... then later, I realize, "Oh, right," and become aware.

**R:** Before, were you immersed in it even afterward?

**P10:** I was less immersed than during the actual confrontation, but I still felt like I was carrying it with me. It felt like stress was piling up. But now, when I look back later, I think, "I didn't need to be that stressed," so it doesn't pile up. It feels lighter.

**R:** What allows it to be lighter? It’s a bit of a difficult question.

**P10:** You can see it as a "reflective" feeling. When I face stress, these days I try to "remind" (replay) it once. While replaying, I also comfort myself. "I didn't need to be that angry or emotional." I learn that and think, "I’ll do it this way next time." I think I’m reflecting.

**R:** Yes, that’s right. Even if you don't do it in the heat of the moment, after some time has passed, you look at it more objectively as you said. You don't get stuck in it; you escape it. That’s what you mean. But what do you mean by "comforting yourself"?

**P10:** Stress can come from my own mistakes. For example, stress from a mistake during practice. My peers would say, "Of course, it’s your first time, it happens," but I’m a bit harsh on myself. I’d think, "How could I make this kind of mistake?" and that piles up as stress for me. I have strict standards for myself. But now, thinking about it at home, I say, "Well, it can happen. It’s natural to not know anything since it's the first time." I try to release the stress that way.

**R:** So before, you were more toward "self-blame," but now you’re toward "understanding yourself" by saying "it can happen." What psychological impact does that have?

**P10:** The biggest thing is that the stressful situation doesn't persist. In future situations where I might make a mistake, the stress of "What if I mess up again?" is reduced. It has the biggest impact on separating my work from my life. I used to keep thinking about it at home, before bed, searching things on the internet... I was so stressed. But now, even if my peer says "it's okay," it used to be hard for me to accept that for myself. But now at home, I say "it can happen" and pat myself on the back. I try to forget the situation. Thinking about it won't solve it anyway; I just shouldn't do it next time.

**R:** Your "inner speech" has changed a lot. You used to speak kindly to others—to your friends, you’d say, "It’s your first time, it’s okay, you can do better next time." You had those compassionate words for friends, but you were harsh to yourself. "How could you do that?" But now you’ve started saying those compassionate words to yourself. If you say to yourself, "It’s okay since it’s the first time, just do better next time," how does that affect you?

**P10:** First of all, the feeling of anxiety decreases. Anxiety is reduced, and I feel like I can have a more stoic attitude when facing similar situations in the future.

**R:** And that stops the worrying, the rushing, and the rumination.

**P10:** Yes.

**R:** I see. Will you keep utilizing this in the future?

**P10:** Yes, I think I need to use this consistently.

**R:** Of course. It’s not as hard as one might think.

**P10:** Right.

**R:** What impact did this have on your nursing clinical practice?

**P10:** The impact on the actual practice... it didn't have a huge impact *during* the clinical situations themselves. But for the stress after coming back from practice, or the anxiety before going... meditation was quite helpful then. On the very first day of practice, you’re so nervous. At that time, I did action meditation and deep breathing, saying "I can do this," and I was able to be stoic. When a stressful situation happened, I’d come home and reflect...

**R:** How do you feel after reflecting like that?

**P10:** After reflecting, I feel a sense of pride, like "I did it." And I feel like I’m comforting myself, which gives me a very warm feeling. It’s good.

**R:** I see. So, as a result, even if you weren't using the skills *in the moment* of practice, by using them before and after, it ultimately helped you during the actual practice. Is that a correct understanding?

**P10:** Yes.

**R:** If you hadn't learned these skills beforehand and just went in... how do you think it would have been?

**P10:** If I hadn't known them, I think clinical practice would have been much harder. I would have carried that anxiety for weeks. Before doing something, I would have felt the full weight of the trembling and anxiety. And when you’re anxious, you make more mistakes. I feel like the anxiety would have been much greater.

**R:** I see. Hearing this makes me very happy.

**R:** I received this kind of treatment (therapy) too...

**P10:** Really?

**R:** Yes, and I was so grateful for it. Now I feel good because it seems I’ve contributed a little to you. Was there anything difficult or disappointing about the MMPT program?

**P10:** Nothing was particularly difficult or disappointing. When you taught us how to meditate, the vocabulary level was easy enough to follow even with eyes closed, so it was very comfortable and good. And I liked that we occasionally repeated and practiced what we did before. It felt like a review. So no disappointments; rather, I liked realizing that there are so many different kinds of meditation.

**R:** I gave you a lot of homework. Was that not difficult?

**P10:** It wasn't difficult. But to be honest, I didn't listen to the audio files you gave very much. But I did the ones during the sessions...

**P10:** If I had to pick a difficulty, it was consistently watching/listening to the recordings for the homework. That wasn't easy. But a good point was being able to share opinions and feelings with various people. I thought, "Oh, this kind of meditation might suit this person," and I realized how diverse people are. It was good to share experiences.

**R:** How does knowing that people are "diverse" affect you, Seung-yeon?

**P10:** Knowing there’s a lot of diversity... for example, I thought everyone would find action meditation and Yoga meditation okay. But I still can't forget that one person said Sensory Meditation was better. Because for me, it just made me sleepy. [Laughs] So it was just fascinating, and I think my "tolerance" or "inclusivity" became a bit wider.

**R:** Inclusivity toward what?

**P10:** Before, I think I used to generalize. I thought, "The public will be like this." But I realized many people prefer different things. So instead of making hasty generalizations, I try not to think [assume] first, but listen to people's opinions.

**R:** So you’ve come to allow/accept that everyone can be different, and different from you. That’s a very good experience. Even in meditation, people are so different; imagine how different thoughts and preferences must be. It must have been a chance for that kind of realization.

**P10:** Yes.

**R:** Good. Through this program, or after it, is there anything new you learned about yourself or anything that changed?

**P10:** What I learned about myself after the program is that I was actually doing quite a bit of self-reflection already. Among the methods you introduced, there were several that I was already doing as a habit. So I felt, "I’ve been living quite well in my own way." And lately, I’ve felt that it’s okay to be less anxious and not get so stressed. I think I’m living a bit more stably now.

**R:** What are some things you were "already doing"?

**P10:** For example, the quotes I mentioned earlier, and observing myself from a third-party perspective... those things. There were more, but they’re such habits that I forgot.

**R:** Well-being behaviors?

**P10:** Well-being behaviors.

**R:** Actions you do to make yourself happy.

**P10:** Like what?

**R:** For example, we looked for actions like "I feel comfortable when I take a walk."

**R:** You were already doing those?

**P10:** Yes.

**R:** So you were already doing well-being behaviors. We also did other things—commonly known ones like writing gratitude journals, well-being journals, or practicing gratitude and wishing compassion for others. Are you referring to those?

**P10:** Yes. I didn't write a gratitude journal, but I’ve been doing "Wishing Compassion" (Metta) continuously.

**R:** You were already doing "Wishing Compassion"?

**P10:** Yes. And wasn't there also... what I do for others, and from my perspective... when people said they had never thought about it and didn't know, I realized I *had* thought about it. I realized, "Oh, some people might not have done this."

**R:** So you, Seung-yeon, had tried those things. Then, did you feel a bit disappointed, like "What is this? I’ve already done all of this. Are we just doing this here too?"

**P10:** Not at all. Rather, I could feel more diversity through it, and I thought it was a great program because we could share feelings and opinions with others. I learned for the first time that people might not have done these things. Sharing examples with them was very interesting and good for me.

**R:** Then, through the program, we learned and practiced each skill one by one. How did learning and practicing those through the program affect the things you were already doing?

**P10:** I didn't know that the actions I was doing were "Well-being Behaviors" or that they had these effects. Through the classes, I learned, "Oh, this was a well-being behavior." It felt like I learned the formal theory behind it, which was fascinating. So I liked it more. Knowing that "theoretically, this is a good action for me" made it feel more concrete.

**R:** Then how does that affect your practice?

**P10:** Well, since it’s already a habit, it’s not like I think "I’m doing a well-being behavior now" while doing it. I just do it as usual.

**R:** But you mean you’ve come to understand what you were doing more concretely. You said you’ve been consistently "Wishing Compassion" for others?

**P10:** Yes. For example...

**R:** Well, first we wish compassion for ourselves. "May I be healthy, happy, peaceful, and live a life of growth." Then we imagine others and wish the same for them. You were already doing that?

**P10:** Yes, I was.

**R:** How did you know to do that?

**P10:** I didn't know it was called "Wishing Compassion." For very close friends or family, I always sincerely cheered for them—"I hope you’re always healthy and happy," "I hope you don't get hurt," "I hope everything you do goes well," "It would be great if you pass your exam." I think I just did it naturally.

**R:** You didn't learn it anywhere... then did you also try wishing it for yourself?

**P10:** I think I wished it more for others than for myself. So I made a resolution to wish compassion for myself too. I did it, but I’m still a bit harsher on myself...

**R:** So you realized that you mainly wished it for others, and you resolved to wish it for yourself too. Right. After that, did the experience of wishing compassion for yourself increase?

**P10:** Yes. But among the things I’ve learned, saying "It’s okay, Seung-yeon" is also a form of compassion, I think. I’ve been comforting and caring for myself a lot in that way.

**R:** Right, that is exactly "Self-Compassion." Since you were someone who practiced compassion for others a lot, you had that basic "strength," and you’ve now shifted that direction toward yourself. Is there any impact on your relationships? Through this program, has there been any impact on your relationships with professors, friends, family, or patients?

**P10:** There hasn't been a specific change in my relationships with others, but there has been a change in my relationship with *myself*. In relationships with others, I don't always perceive things clearly in the heat of the moment; I realize and reflect on them later. So my behavior in the moment is similar. But through consistent practice, I think my relationship with myself has changed.

**R:** What kind of change?

**P10:** In the relationship with myself... I’m less harsh. I’ve come to recognize more that "I am also a person who can be like that [make mistakes]."

**R:** How does your heart feel when you recognize that?

**P10:** When I recognize it, I feel less anxious and I become more compassionate toward myself.

**R:** Yes.

**P10:** I’ve escaped a bit from my strict standards to comfort myself. I have fewer distracting thoughts, and through action meditation, I’ve reduced anxiety, stress, and random thoughts. I think all of those have come to me as changes in my relationship [with myself]. More stably.

**R:** I can feel that you worked very hard.

**P10:** Really?

**R:** Of course. Learning is important, but how much you "practiced" matters. As you said, habit formation is key. What you know in your head must reach your body and heart. Seeing how much the things in your head have moved down to your body and heart, it’s clear you practiced and made an effort to form habits. I can feel that clearly, so I’m very proud. I think you’ll continue to utilize this well. I can feel a certain "firmness" in your voice. By continuing to use this, you’ll make it a part of your life. Since this is a skill where you backslide if you stop but move forward if you continue, I hope you use it well. How do you feel after today? You might have wondered if we could talk for an hour with just these questions.

**P10:** Exactly. I did wonder, but now that we’ve talked... it wasn't actually that long. [Laughs]

**R:** Right. Do you know what one participant said at the end? They said with awe, "Wow, this really takes an hour."

**P10:** I see.

**R:** When I first sent the questionnaire, they thought, "Isn't this going to be over in 10 minutes?" They wondered how we could fill an hour. At the end, it just slipped out of their mouth. It was quite funny. Now that we’ve shared these stories, how is your heart? Or what thoughts do you have?

**P10:** I feel like I could look back on everything I’ve done. By reminding myself of what I did, I think, "I should keep doing this consistently in the future."

**R:** It sounds like it was a time for organizing your thoughts and getting motivated. That’s great.

**R:** Good. Anyway, I’m so glad to have made this connection. I wish for you to become a wonderful nurse. All the best.

**P10:** Yes, thank you. Goodbye. I’ll be going now.

**R:** Yes.
