# Supplementary material for: Pilot study on the effect of a Meditation–Mindfulness–Positive Psychology Training program on perceived stress and mental well-being in Korean nursing students: A mixed methods analysis
Source: PLoS One. 2026 Apr 21;21(4):e0345139. doi: 10.1371/journal.pone.0345139 (PMC13098892; doi:10.1371/journal.pone.0345139)
Supplement: Attachment — Submitted filename: PLOSONELETTER.docx [file pone.0345139.s003.docx]

Manuscript ID: PONE-D-25-49792

Title: Pilot study on the effect of a Mindfulness-Meditation-Positive-Psychology-Training program on perceived stress and mental well-being in Korean nursing students: A mixed methods analysis

Dear Editors,

Thank you for your message regarding our submission.

We would like to clarify that the present manuscript (PONE-D-25-49792) is a completely new study and not a resubmission of PONE-D-25-17820. The two manuscripts differ in sample, study design, analysis, and content. Therefore, a rebuttal letter in response to the comments on PONE-D-25-17820 is not applicable in this case.

In compliance with your request, we have uploaded the original IRB approval documents in Korean along with the previously provided English translations.

We hope this resolves the editorial concerns and respectfully submit our manuscript and documents for further consideration.

Sincerely,

Prof. Dong Hee Kim
